# Synthesis, Characterization, In Vitro Anticancer Potentiality, and Antimicrobial Activities of Novel Peptide–Glycyrrhetinic-Acid-Based Derivatives

**DOI:** 10.3390/molecules26154573

**Published:** 2021-07-28

**Authors:** Gaber O. Moustafa, Ahmed Shalaby, Ahmed M. Naglah, Marwa M. Mounier, Heba El-Sayed, Manal M. Anwar, Eman S. Nossier

**Affiliations:** 1 National Research Centre, Peptide Chemistry Department, Chemical Industries Research Division, Cairo 12622, Egypt; ahshalaby@gmail.com; 2 Department of Pharmaceutical Chemistry, Drug Exploration and Development Chair (DEDC), College of Pharmacy, King Saud University, Riyadh 11451, Saudi Arabia; 3 National Research Centre, Pharmacognosy Department, Pharmaceutical and Drug Industries Research Division, 33-El Bohouth St., Giza 12622, Egypt; marwa_m3@yahoo.com; 4 Botany and Microbiology Department, Faculty of Science, Helwan University, Helwan 11111, Egypt; drhebaelsayed39@gmail.com; 5 National Research Centre, Department of Therapeutic Chemistry, Cairo 12622, Egypt; manal.hasan52@live.com; 6 Department of Pharmaceutical Medicinal Chemistry and Drug Design, Faculty of Pharmacy (Girls), Al-Azhar University, Cairo 11754, Egypt; dr.emannossier@gmail.com

**Keywords:** glycyrrhetinic acid, peptide derivatives, anticancer potentiality, apoptotic markers, antimicrobial activity, molecular docking studies

## Abstract

Glycyrrhetinic acid (GA) is one of many interesting pentacyclic triterpenoids showing significant anticancer activity by triggering apoptosis in tumor cell lines. This study deals with the design and synthesis of new glycyrrhetinic acid (GA)–amino acid peptides and peptide ester derivatives. The structures of the new derivatives were established through various spectral and microanalytical data. The novel compounds were screened for their in vitro cytotoxic activity. The evaluation results showed that the new peptides produced promising cytotoxic activity against the human breast MCF-7 cancer cell line while comparing to doxorubicin. On the other hand, only compounds **3**, **5**, and **7** produced potent activity against human colon HCT-116 cancer cell line. The human liver cancer (HepG-2) cell line represented a higher sensitivity to peptide **7** (IC_50_; 3.30 μg/mL), while it appeared insensitive to the rest of the tested peptides. Furthermore, compounds **1**, **3**, and **5** exhibited a promising safety profile against human normal skin fibroblasts cell line BJ-1. In order to investigate the mode of action, compound **5** was selected as a representative example to study its in vitro effect against the apoptotic parameters and Bax/BCL-2/p53/caspase-7/caspase-3/tubulin, and DNA fragmentation to investigate beta (TUBb). Additionally, all the new analogues were subjected to antimicrobial assay against a panel of Gram-positive and Gram-negative bacteria and the yeast candida Albicans. All the tested GA analogues **1**–**8** exhibited more antibacterial effect against Micrococcus Luteus than gentamicin, but they exhibited moderate antimicrobial activity against the tested bacterial and yeast strains. Molecular docking studies were also simulated for compound **5** to give better rationalization and put insight to the features of its structure.

## 1. Introduction

Natural products are estimated as a rich reservoir of multiple compounds providing a wide spectrum of therapeutic potentials. Many natural products were isolated from microbes, plants, and other living organisms to be evaluated as anticancer candidates and explore their different mechanisms of action. These efforts resulted in the discovery and development of a group of promising anticancer therapeutics. In the last three decades, about 25% of all newly approved anticancer drugs were of natural origin [1,2]. Furthermore, many anticancer compounds with unique structural advantages in probing druggable modalities have been reported. Optimization of bioactive natural products into drugs is still a challenging skill, due to the difficulty in large-scale isolation as well as mechanistic understanding and pharmaceutical development. Recent studies showed that due to the rapid advancement of cancer therapy knowledge and innovative technologies, it is now possible to overcome obstacles such as increasing drug efficiency, identifying natural product direct targets, and resolving the complexity of multi-faceted pharmacological effects [3,4].

Pentacyclic triterpenoids (PTs) are the largest group of natural compounds found in plants. Multiple studies have exhibited that there are a considerable number of PTs revealed significant cytotoxic activity against a variety of cancer cells as well as antitumor efficacy in preclinical animal models [5,6,7,8,9]. They induce their anticancer activity via enhancement of cell apoptosis resulting in suppressing tumor growth. PTs trigger apoptosis by various mechanisms, such as interfering with the mitochondrial function of the cancerous cells [10], upregulating the oncogenic markers p53 and caspase-3 genes, suppressing the NF-κB that mediates the activation of Bcl-2 [11] in addition to promoting tumor necrosis factor (TNF)-related apoptosis-inducing ligand (TRAIL) [12,13]. Additionally, it has been revealed that terpenoids suppress the activity of STAT3, which is stimulated by interleukin-6 in DLD-1 colon cancer cells [14].

**Figure 1:**
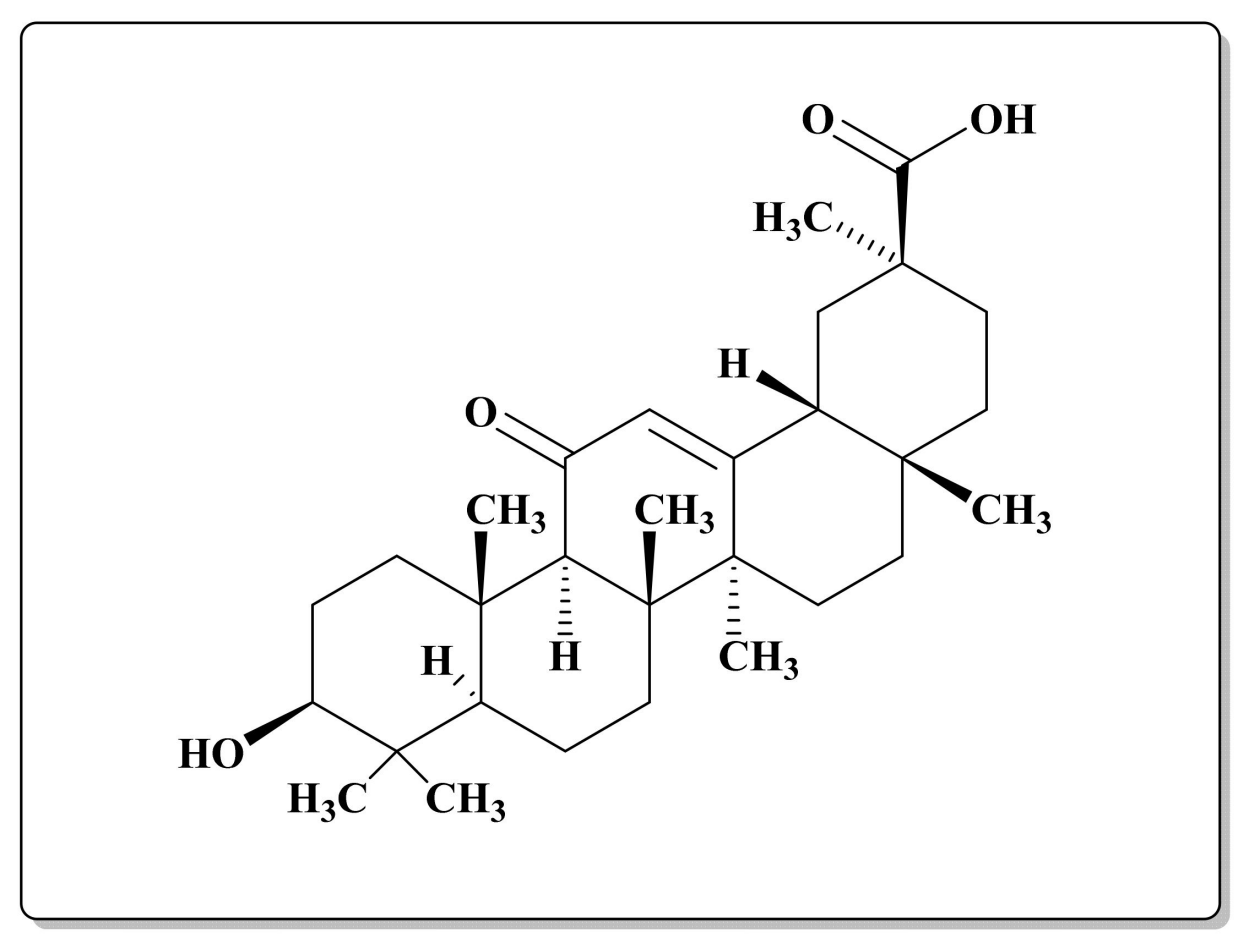
Chemical structure of 18 β-glycyrrhetinic acid (GA).

Licorice root is a medicinal plant. It contains many phytochemicals including more than 300 flavonoids and 20 triterpenoids. The active ingredients, glycyrrhizic acid (GL), glabrin A and B, 18 β-glycyrrhetinic acid (GA, the major metabolite of GL) (Figure 1), isoflavones, and others produce different pharmacological activities. Glycyrrhizin, an abundant bioactive component, is promptly metabolized into 18 β-glycyrrhetinic acid by the gut commensal bacteria [15]. GL and GA are kinds of pentacyclic triterpenoids, having many valuable pharmacological properties, such as antioxidant properties as well as potent anti-inflammatory, antiviral, antitumor, and immune-regulatory properties [15,16,17,18,19,20].

The anticancer activity of GA has been extensively studied against different cancer types, such as breast cancer, ovarian cancer, promyelocytic leukemia, hepatoma, and stomach cancer cells [21]. It has been investigated that GA can produce the cytotoxic effect via various modes of action, such as disruption of actin cytoskeleton, suppression of the p38 MAPK-AP1 signaling axis in addition to induction of Fas- or DNA fragmentation-mediated apoptosis [22,23,24,25]. Fortunately, the studies represented the safety profile of GA, since it produces selective cytotoxicity against tumor cells without any undesirable side effects on normal cells in addition to its potent anticancer effect higher than some clinically available antitumor drugs [21]. However, due to the high hydrophobicity and poor blood serum solubilization of the pentacyclic triterpenoids, GA use as a cytotoxic medicine in the treatment of cancer disease is limited. Up until now, numerous studies were directed to structural modifications to enhance GA bioavailability, selectively, and cytotoxicity [26,27,28,29,30]. Amino acids are the primary substance, which support biological life activities, producing very important roles. They are highly soluble in water having a wide spectrum of pharmacological activities including antitumor activity [31]. It has been reported that conjugation of pentacyclic triterpenoids with various amino acid residues can modify their shortcomings resulting in enhancing their physicochemical characteristics and ADME properties. Therefore, they have been considered as the preferred natural product structural modifiers [32].

Furthermore, it has been reported that different cancer types are mainly caused by various factors including certain types of infections [32]. Various antibiotic cytotoxic drugs including Actinomycin, Adriamycin/Doxorubicin, and some other candidates produce their activity by DNA intercalating or DNA-damaging effects in addition to stimulation of the existing host defense mechanism, which is considered as one of the modes of actions that cause these chemotherapeutics to induce their antimicrobial effects [33].

Based on the afro-mentioned investigations, aiming to modify and improve the bioactivity of GA and in continuation of our strenuous efforts in the field of discovery of new potent anticancer and antimicrobial agents [26,27,28,29,30,34,35,36,37,38,39,40,41,42,43], this work represents a facile structural modification strategy to synthesize novel 18β-glycyrrhetinic amide derivatives via its coupling with different amino acids. The new compounds were evaluated as anticancer candidates against three human cancer cells; colon cancer HCT-116, breast cancer (MCF-7), and liver cancer (HepG-2) cell lines. The promising compound revealing the highest anticancer activity **5** was selected as a representative example for further assessments to find out its mechanistic modes of action such as its impact on various oncogenic parameters: Bcl-2, Bax, p53, caspase-3, caspase-7, tubulin polymerization (TubB), and % of DNA fragmentation. A molecular modelling study was also performed for the same compound to elucidate its binding modes with the amino acid residues of caspase-3 and Bcl-2 to rationalize its promising pharmacological effect. In addition, since various studies investigated the significant antibacterial activity of different GA analogues against different pathogenic bacterial strains [44,45,46,47], it was of interest to study the antimicrobial activity of the newly synthesized GApeptides aiming to reach to new safe agents of potent dual anticancer and microbial efficacy of natural origin, so the new derivatives were examined as antimicrobial agents against a panel of Gram-positive and Gram-negative bacterial and yeast strains.

## 2. Results and Discussion

### 2.1. Chemistry

**Scheme 1:**
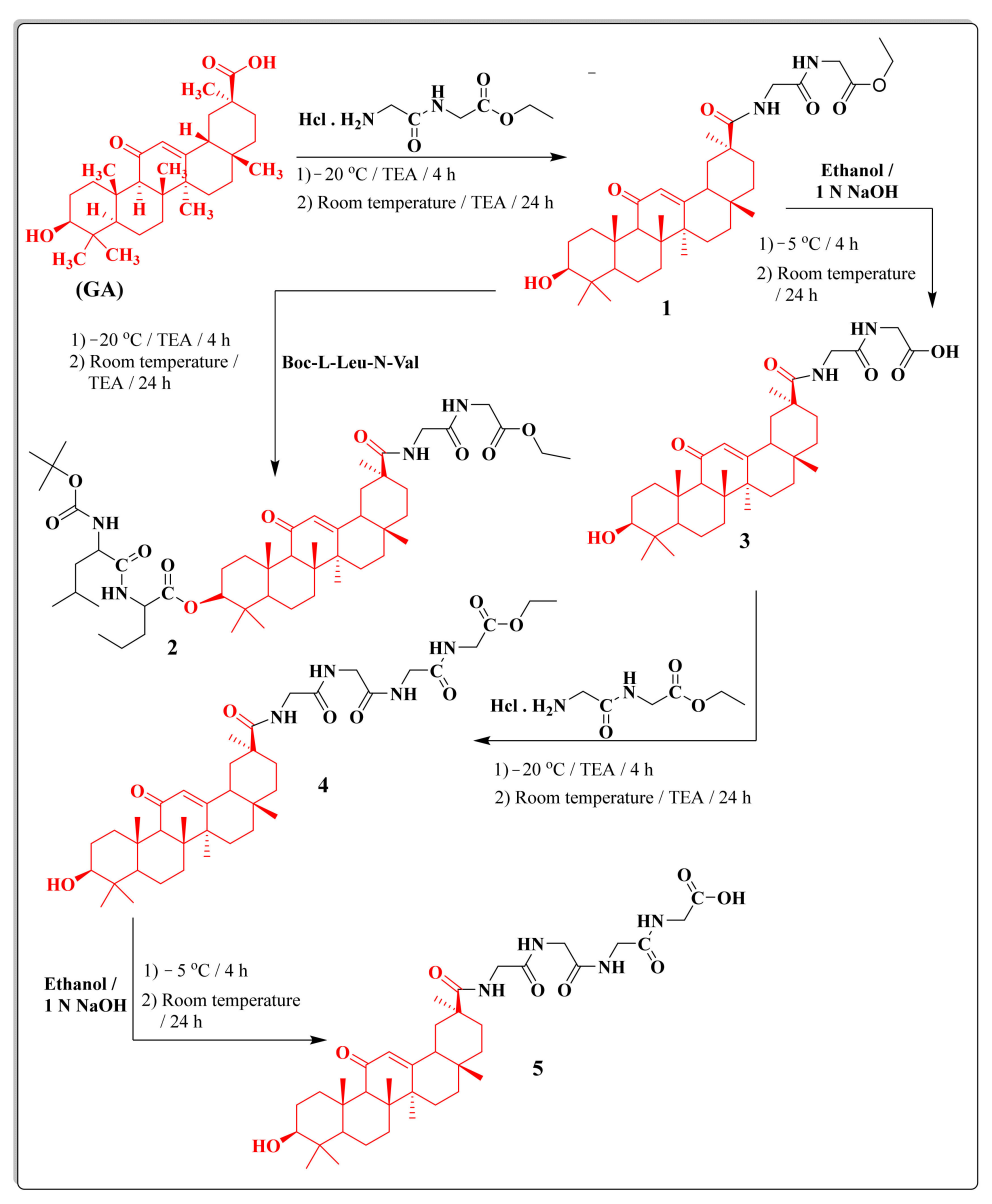
The synthetic routes for 18β-glycyrrhetinic-acid-based peptides **1**–**5**.

One of our goals was increasing the activity of GA by “simple” transformation but retaining the advantages residing in the parent compound GA. Thus, this study aims to conjugate 18β-glycyrrhetinic acid with different amino acids to gain new peptide candidates **1**–**8**. The synthetic approaches of construction of the eight novel peptides **1**–**8** are illustrated in Scheme 1. The acid 18β-glycyrrhetinic acid was coupled with various amino acids utilizing conventional synthetic peptide coupling methods (in solution) [48].

Condensation of 18β-glycyrrhetinic acid with ethyl glycylglycinate hydrochloride afforded the corresponding ester derivative **1**. The reaction of compound **1** with Boc-l-Leu-*N*-Val yielded the derivative **2**, while the hydrolysis with methanolic sodium hydroxide produced the acid derivative **3**. In addition, condensation of acid derivative **3** with ethyl glycylglycinate hydrochloride afforded compound **4**. Additionally, hydrolysis of compound **4** with methanolic sodium hydroxide produced compound **5** (Scheme 1). The structures of the new compounds were confirmed depending upon microanalytical and spectral data. IR spectra of the new compound **1** revealed well-defined bands at 1752 cm^−1^ referring to (C=O ester), 1659, 1535, and 1457 cm^−1^ referring to three (C=O) groups, respectively. Furthermore, ^1^H NMR spectrum of **1** represented the characteristic triplet-quartet signals of the ethyl group at δ 1.40 and 4.33 ppm. ^1^H NMR spectrum of **2** showed characteristic singlet signals at δ 9.04, 9.00, 8.45, and 7.55 ppm due to four CONH amide protons, a quartet signal at δ 1.90 ppm due to CH_2_CH_2_CHofL-Nva. Moreover, ^1^H NMR spectrum of **2** revealed the presence of two triplets at δ 1.80 and 1.50 ppm related to the CH and CH_2_of Leu. IR spectrum of the compound **3** showed strong absorption bands at 3610 and 3509 cm^−1^ referring to OH primary cyclic alcohol and OH acid.

**Scheme 2:**
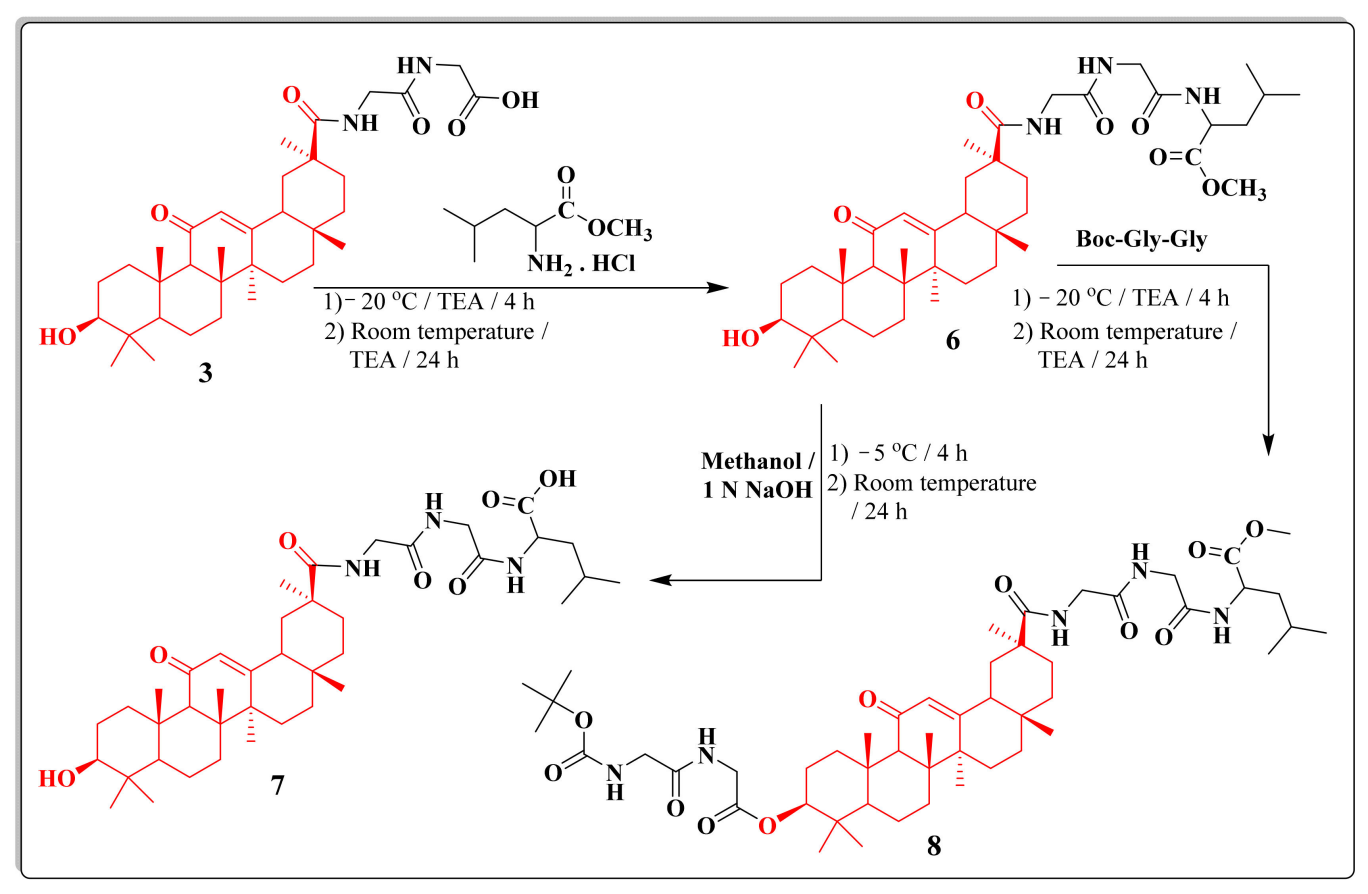
The synthetic routes of the 18β-glycyrrhetinic-acid-based peptides **6**–**8**.

Moreover, condensation of acid derivative **3** with methyl leucinate hydrochloride yielded the methyl ester derivative **6**. The hydrolysis of compound **6** with ethanolic sodium hydroxide gave the acid derivative **7**, while the reaction with Boc-Gly-Gly produced the derivative **8** (Scheme 2). ^1^H NMR spectrum of compound **6** revealed the presence of asinglet signal at δ 3.70 ppm related to the ethoxy group, a triplet signal at δ 1.55 ppm referring to CH of L-Leu, and a doublet signal at δ 1.05 ppm due to two CH_3_ groups of *L*-Leu.^1^H NMR spectrum of compound **7** revealed the presence of a singlet signal at δ 12.60 ppm related to the carboxylic acid group and the disappearance the signal of the ethoxy ester group.

### 2.2. Biological Activity

#### 2.2.1. Anticancer Evaluation

**Figure 2:**
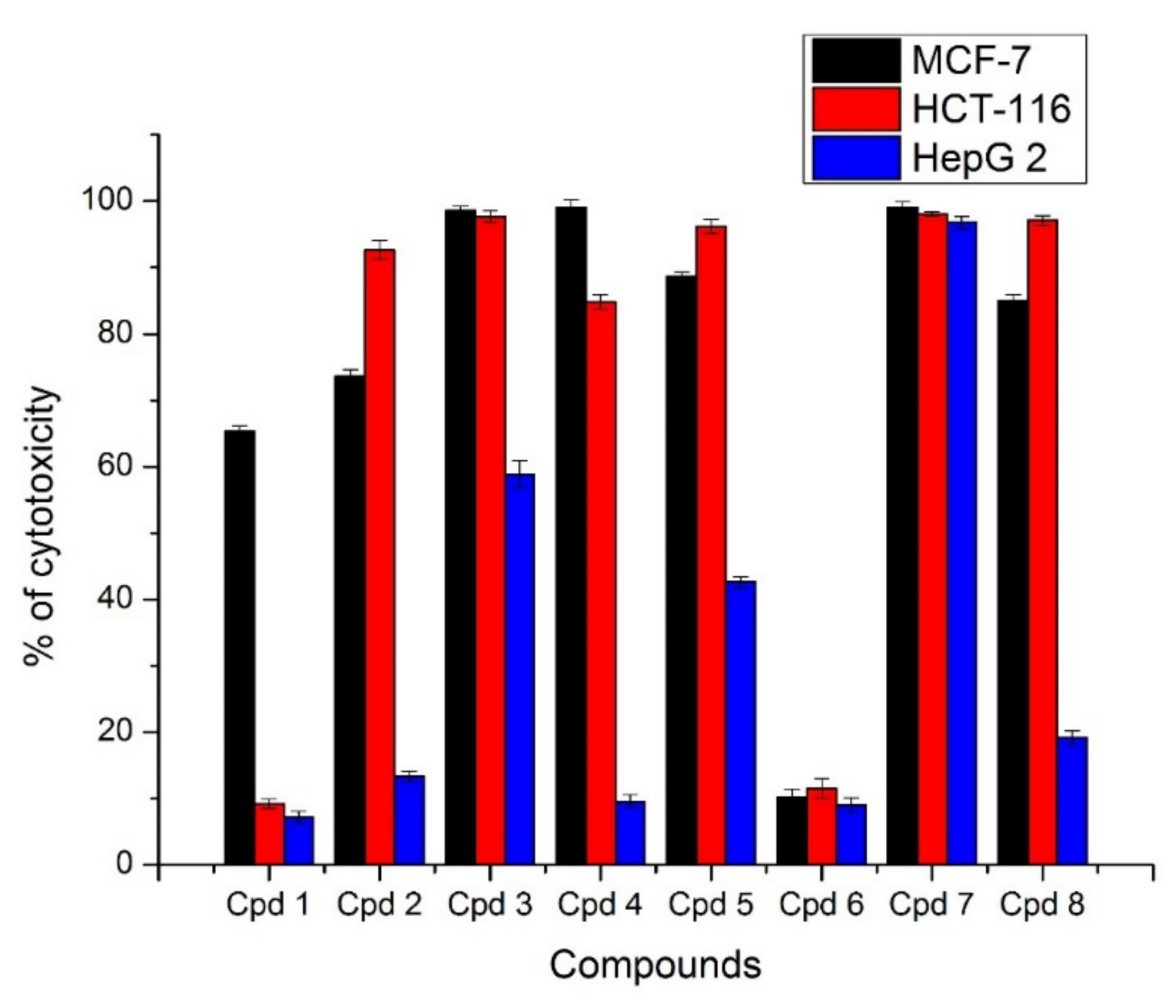
In vitro cytotoxic activity of compounds **1**–**8** at concentration 10 µg/mL against different cancer cell lines. The results are represented as % inhibition ± standard deviation. Each result is the mean of three replicates.

All the GA peptides **1**–**8** were preliminary screened for their cytotoxic activity by measuring there in vitro effects on the viability of different three human cancer cell lines: namely, the breast MCF-7, the colon HCT-116, and the liver HepG-2 cell lines. From the obtained results, all the tested peptides except **1** and **6** revealed remarkable mitochondrial-dependent cytotoxicity by reducing the viability of the breast tumor cells at a starting concentration 10 µg/mL with cytotoxicity percentages of 99.06, 99, 98.6, 88.6, 84.96, and 73.6%, respectively, where compound **1** showed a moderate effect with 65.3% cytotoxicity and compound **6** with a weak cytotoxic effect. Regarding the colon cells HCT-116, compounds **7**, **3**, **8**, **5**, **2**, and **4** showed the most cytotoxic activity with percentages of 98.06, 97.66, 97.10, 96.16, 92.63, and 84.80, respectively, while the compounds **1** and **6** displayed a weak effect. On the other hand, only compound **7** illustrated a potent activity over liver carcinoma cells HepG-2 with a percentage of 96.76 (Figure 2).

The promising compounds that exhibited percentage of inhibition ≥70% were further subjected to MTT assay for investigation of their IC_50_ (median growth inhibitory concentration necessary to inhibit the cell viability by 50%) [34,35,36] and doxorubicin served as a reference. Accordingly, all the newly synthesized GA-based peptides except **1** and **6** were screened for their cytotoxicity against three different human cancer cell lines: MCF-7, HCT-116, and HepG-2 cell lines at different concentrations (10, 5, 2.5, 1.25, and 0.625 µg/mL). The resultant data were expressed as half maximal inhibitory concentration IC_50_ (μg/mL) and summarized in Table 1.

**Figure 3:**
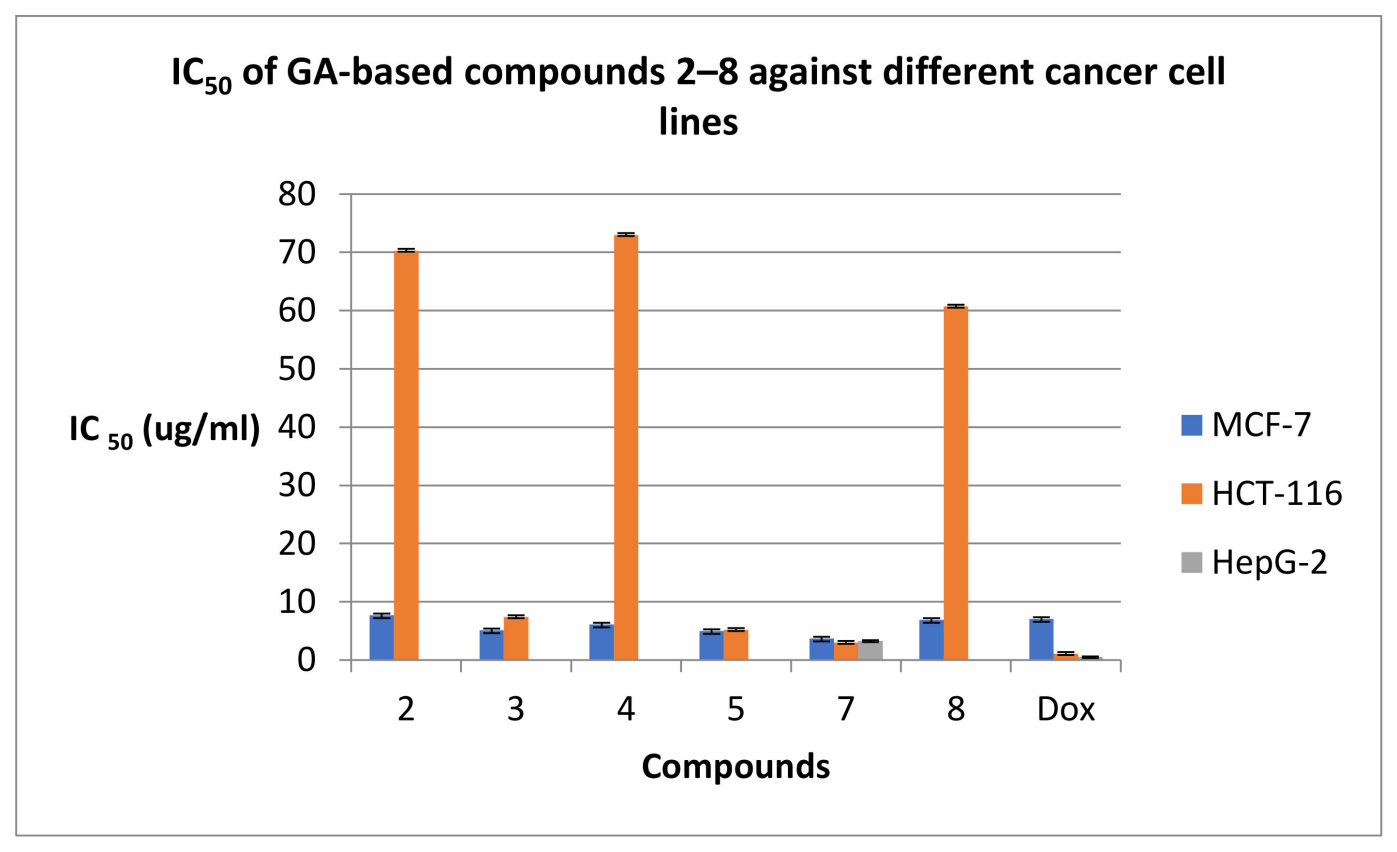
IC_50_ values of GApeptides **2**–**8** against various human cancer cell lines.

Based on Table 1, it could be investigated that the new peptides **2**, **3**, **4**, **5**, **7**, and **8** exhibited more potent activity than the reference drug doxorubicin against the breast cancer cells exhibiting IC_50_ values ranging from 3.70 to 7.70 μg/mL in comparing the IC_50_ values of doxorubicin 7.07 μg/mL. Table 1 represented that the newly synthesized GA-based peptides were mostly selective for human breast MCF-7 growth inhibition. Compounds **3**, **5**, and **7** were more potent inhibitors against MCF-7 cell lines with IC_50_ 5.10, 5, and 3.70 μg/mL, respectively, than the remaining the *O*-alkylated peptides **2**, **4**, and **8** with IC_50_ 7.70, 6.10, and 6.90 μg/mL, respectively. The more significant cytotoxic activity of the peptides **3**, **5**, and **7** than the other ones could be explained due the presence of the free hydroxyl group at C-30, which might act as a H-donor serving for H-binding with various amino residues in the active sites of the target proteins that they act on. With respect to the HCT-116 cell line, the same compounds **3**, **5**, and **7** also produced significant cytotoxic activity of IC_50_ 7.40, 5.2, and 3.0 μg/mL in comparing to remaining compounds. On the other hand, the rest of the compounds exhibited a dramatic drop in the activity on colon cancer cells HCT-116 with an IC_50_ range of 60.70–73.0 μg/mL. Interestingly, only compound **7** exhibited more potent growth inhibition activity against the HepG-2 cell line (IC_50_; 3.30 μg/mL), while the activity was completely lost by the rest of the assessed peptides (Figure 3).

#### 2.2.2. The Effect of the GA Peptides against the Normal Skin Fibroblasts Cell Line BJ-1

**Figure 4:**
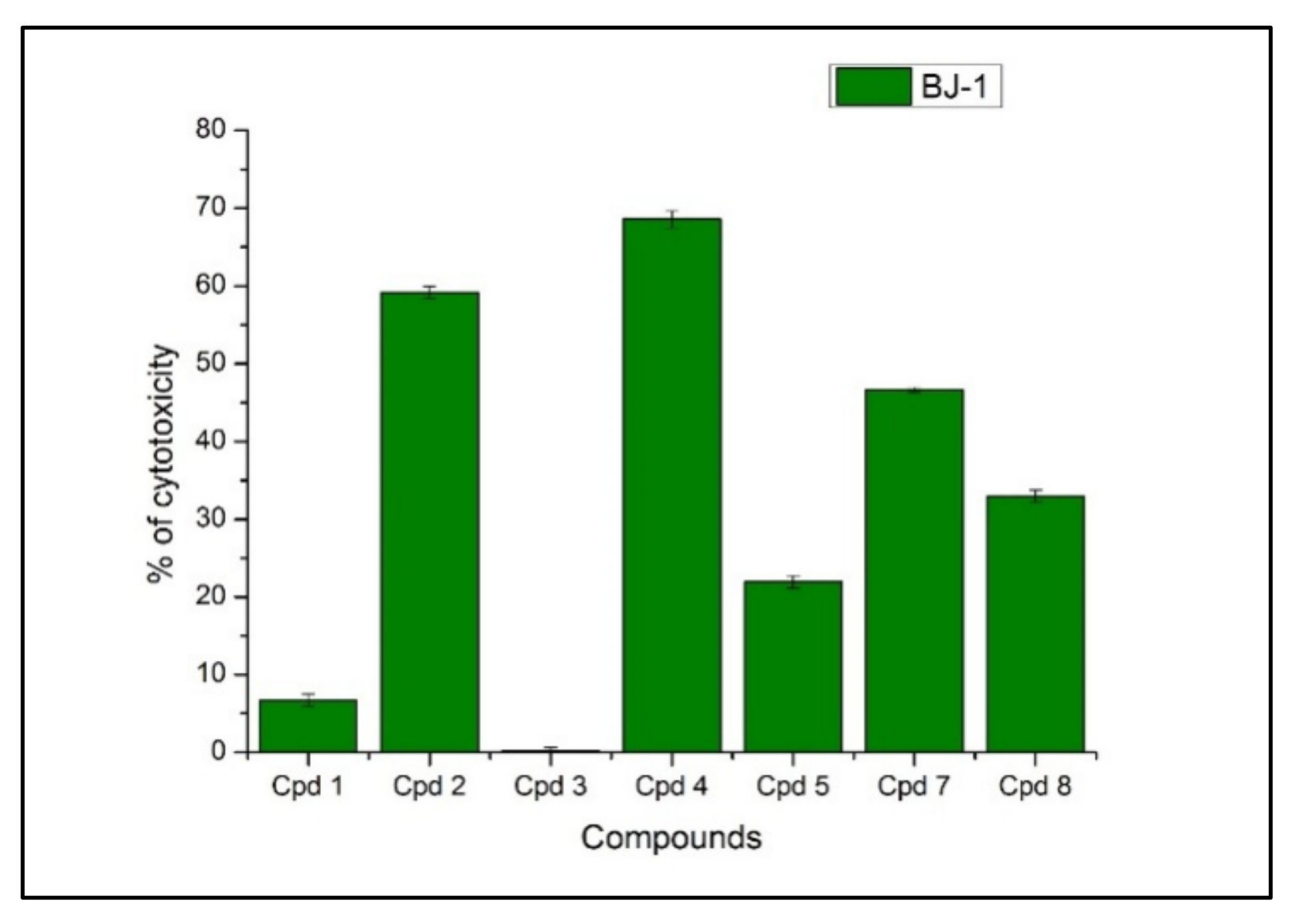
Cytotoxic activity of the newly synthesized GA peptides against the normal skin human cell line BJ-1 at 10 ug/mL.

The frequency and severity of side effects to normal cells at therapeutic levels is one of the most critical factors that distinguish different anticancer drugs from each other. Accordingly, the GA peptides **1**–**8** were furthermore screened for their cytotoxicity at 10 µg/mL against the human normal skin fibroblasts cell line BJ-1 to determine their safety upon normal cells (Figure 4). The compounds **1**, **3**, and **5** exhibited minor cytotoxic effects of 7%, 2%, and 20% on the examined normal cells compared to the other compounds. According to our safety study on BJ-1, compound **5** was chosen from among compounds **1** and **3** due to its remarkable selective anti-breast carcinoma cells in a dose-dependent manner with the least IC_50_ = 5 ug/mL without affecting normal healthy cells, which makes compound **5** an interesting GA peptide for further biological evaluation.

#### 2.2.3. The Effect of the GA Peptide 5 on Bax, Bcl-2, and p53 Levels

**Figure 5:**
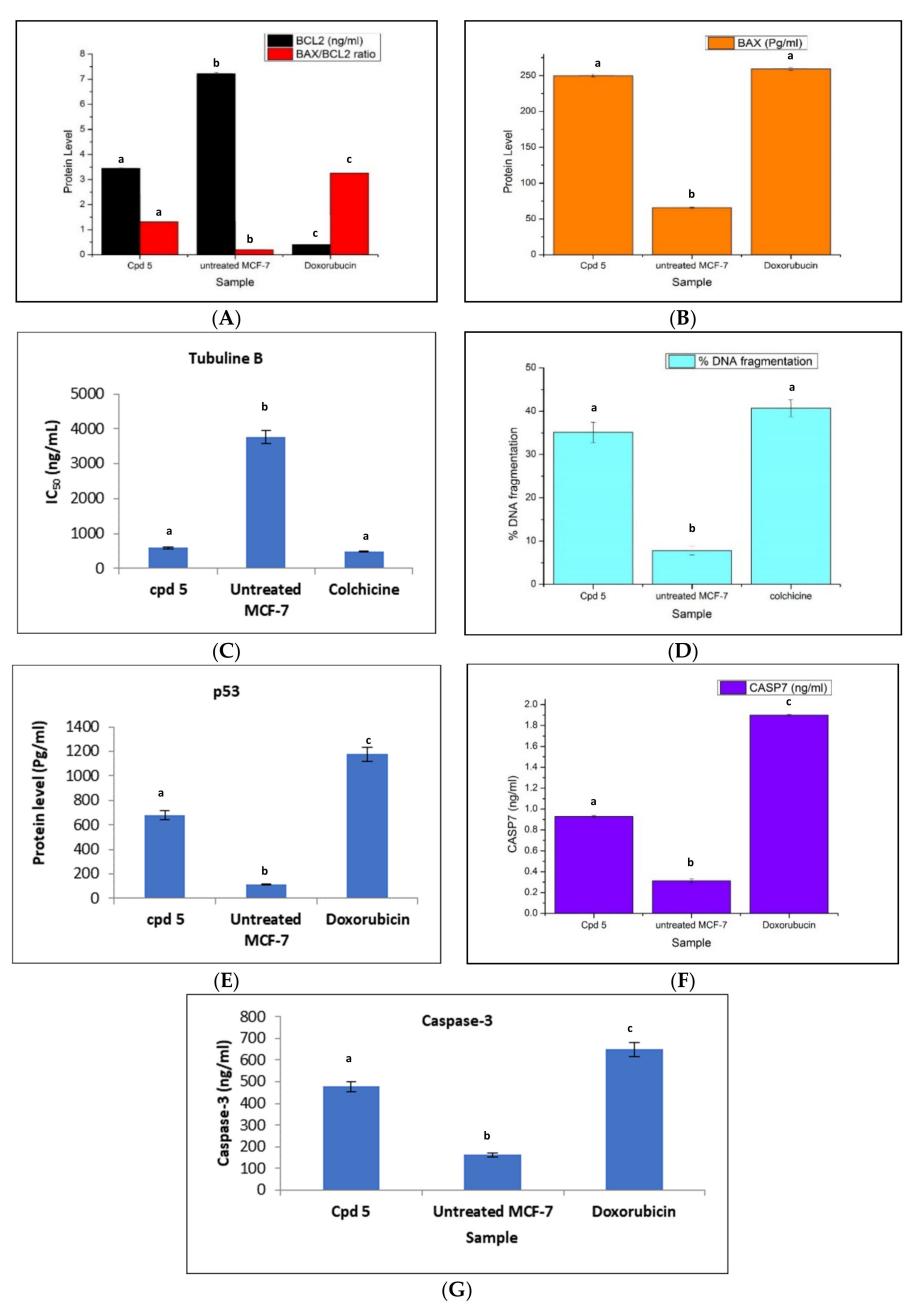
Quantitative assessment (mean ± SD) protein levels of (**A**) Bax/BCL-2 ratio, (**A**) Bcl-2 (ng/mL), (**B**) Bax (Pg/mL), (**C**) IC_50_ (ng/mL) on tubuline B, (**D**) % DNA fragmentation, (**E**) p53 (Pg/mL), (**F**) caspase-7(ng/mL) and (**G**) caspase-3(ng/mL) for compound **5**, each value is the mean of three replicates, SD = standard deviation. Statistical analysis was done using one-way analysis of variance (ANOVA) using the CoStat computer program accompanied by the least significant level (LSD) between groups at *p* < 0.05. Unshared letters (a, b, and c) are significant values between groups for each parameter at *p* < 0.0001.

The extrinsic pathway (death receptor) and the intrinsic pathway (mitochondrial route) are the main two routes that control a cell during apoptosis [48]. The Bcl-2 protein introduces a central role in tumor growth or suppression the intrinsic apoptotic pathway [49]. Conversely, the pro-apoptotic Bax acts essentially to enhance the process of cell apoptosis [50,51,52,53]. Increasing the level of the anti-apoptotic Bcl-2 within the cancerous cell boosts the cell survival through inhibiting apoptosis [50]. Accordingly, a balance situation between these two proteins determines the cell fate [51]. Furthermore, p53 serves as a transcription factor acting as a tumor suppressor protein. This protein is expressed due to various cellular stresses such as DNA disruption [54]. It is usually mutated in human cancers [55]. The p53 pathway mediates either cell cycle arrest or apoptosis controlling the expression of Bcl-2 and Bax genes [55]. Thus, since compound **5** produced the most promising cytotoxic activity against breast cancer cell lines with a safety profile against the normal cells, it was of interest to study its impact on the regulation of the oncogenic markers Bax, Bcl-2, and p53. MCF-7 cancer cells were incubated with compound **5** at its IC_50_ 5 μg/mL for 24 h. The obtained results represented that compound **5** has decreased the level of Bcl-2 from 7.20 ng/mL in the untreated MCF-7 cells to 3.40 ng/mL in the treated ones. Furthermore, compound **5** has boosted the level of Bax protein from 65.50 Pg/mL in untreated MCF-7 cells to 249.9 Pg/mL in the treated cells, which was very close to the Bax level obtained by doxorubicin 259.2 Pg/mL. From the previous results, the screened peptide **5** represented a predictable ability to elevate the therapeutic response in MCF-7 cells that was supported by its disruption in the Bax/Bcl-2 ratio from 0.2 in untreated MCF-7 to 1.3 in one treated with compound **5** (Figure 5). Additionally, compound **5** has raised the level of p53 by approximately six-fold from 111.07 Pg/mL in untreated MCF-7 to 679.8 Pg/mL in the treated cells.

#### 2.2.4. Effect of the Peptide 5 on the Level of Caspase-3, Caspase-7, Tubulin Polymerization (TubB), and % of DNA Fragmentation

Caspases are a family of cysteine proteases that are responsible for launching the programmed cell death (apoptosis). Various reports revealed that some kinds of caspases are sponsors for starting intracellular event cascade, while others are effectors, producing their effects further downstream resulting in cellular breakdown by intrinsic protein breakdown. Cell shrinkage, chromatin condensation, and DNA fragmentation are all apoptosis events, which need the involvement of caspase-7and caspase-3 [48,54,56]. Accordingly, MCF-7 cells were treated with **5** at its IC_50_ concentration of 5 μg/mL for 24 h aiming to detect the change in the level of caspase-3and caspase-7. The result data revealed that compound **5** afforded about a three-fold level elevation from 0.3 ng/mL in the untreated cells to 0.9 ng/mL in the treated MCF-7 cells, but less than the level obtained by doxorubicin (1.9 ng/mL) for caspase−7 and from 162 ng/mL in the untreated cells to 477 ng/mL in the treated MCF-7 cells, but less than the level obtained by doxorubicin (650 ng/mL) forcaspase-3 (Figure 5). Furthermore, compound **5** has also produced a drastic effect on DNA and inhibition of tubulin polymerization in the treated MCF-7 cells. In addition, compound **5** enhanced the percentage of DNA fragmentation of 35%in the treated MCF-7 cells by about 4.5-fold times the percent in the untreated cells of 7.8%and was almost obtained by the reference drug colchicine (40.7%), as shown in (Figure 5). At the same time, compound **5** affected the microtubules mass proteins tubulin B, which displays a central role in cell proliferation through inhibition of the tubulin polymerization of MCF-7with IC_50_, 592.5 ng/mL, whereas the reference drug colchicine represented an IC_50_ value of 487.7ng/mL (Figure 5). In brief, GA peptide **5** represented potent pro-apoptotic activity by the induction of the intrinsic mitochondrial pathway of apoptosis.

#### 2.2.5. Antimicrobial Activity

The newly synthesized peptides **1**–**8** (compound **6** was not tested) was evaluated for their antimicrobial activity of against three pathogenic Gram-positive bacteria (*Staphylococcus aureus ATCC25923*, *Streptococcus pneumoniaRCMB 010010*, and *Micrococcus Luteus)*, three pathogenic Gram-negative bacteria viz. *Escherichia coli ATCC25922*, *Pseudomonas aeruginosa ATCC7853*, and *Proteus vulgaris RCMB 010085*, and the yeast *Candida albicans* using the well diffusion method. Ketoconazole and gentamycin were utilized as references for the antifungal and antibacterial activity, respectively [57]. The obtained data were documented for each derivative as the average diameter of the inhibition zones (IZ) in mm for the bacterial or the yeast growth around the discs (Table 2). Obviously, all the tested GA analogues **1**–**8** exhibited more potent growth inhibition activity against Micrococcus Luteus than gentamicin (IZ ranging from 29 to 30 mm, IZ Gentamicin 24.4 ±0.72 mm). Although Micrococcus Luteus bacteria is a normal commensal of human skin and the upper respiratory tract, it acts as an opportunistic pathogen and has been associated with various diseases including meningitis, septic arthritis, and endocarditis [58]. On the other hand, all the tested compounds exhibited moderate activity against the screened yeast and bacterial strains of IZ range 12–15 mm comparing to ketoconazole of IZ 22.8 ± 0.10 mm and IZ ranging from 12 to 18 mm comparing to the reference drug Gentamicin of IZ ranging from 20 to 27 mm.

### 2.3. Molecular Docking Study on Caspase-3 and Bcl-2

In order to shed the light on the mechanisms by which the promising derivative 5 causes activation of caspase-3 and inhibition of Bcl-2, docking study was utilized using Molecular Operating Environment (MOE-Dock) software version 2014.0901 [59,60,61] to explore the possible interactions between target 5 and the two proteins. The crystal structures of caspase-3 (PDB code: 2J30) [62] and Bcl-2 (PDB code: 4LVT) [63] was downloaded from the RCSB Protein Data Bank and prepared for the docking process.

**Figure 6:**
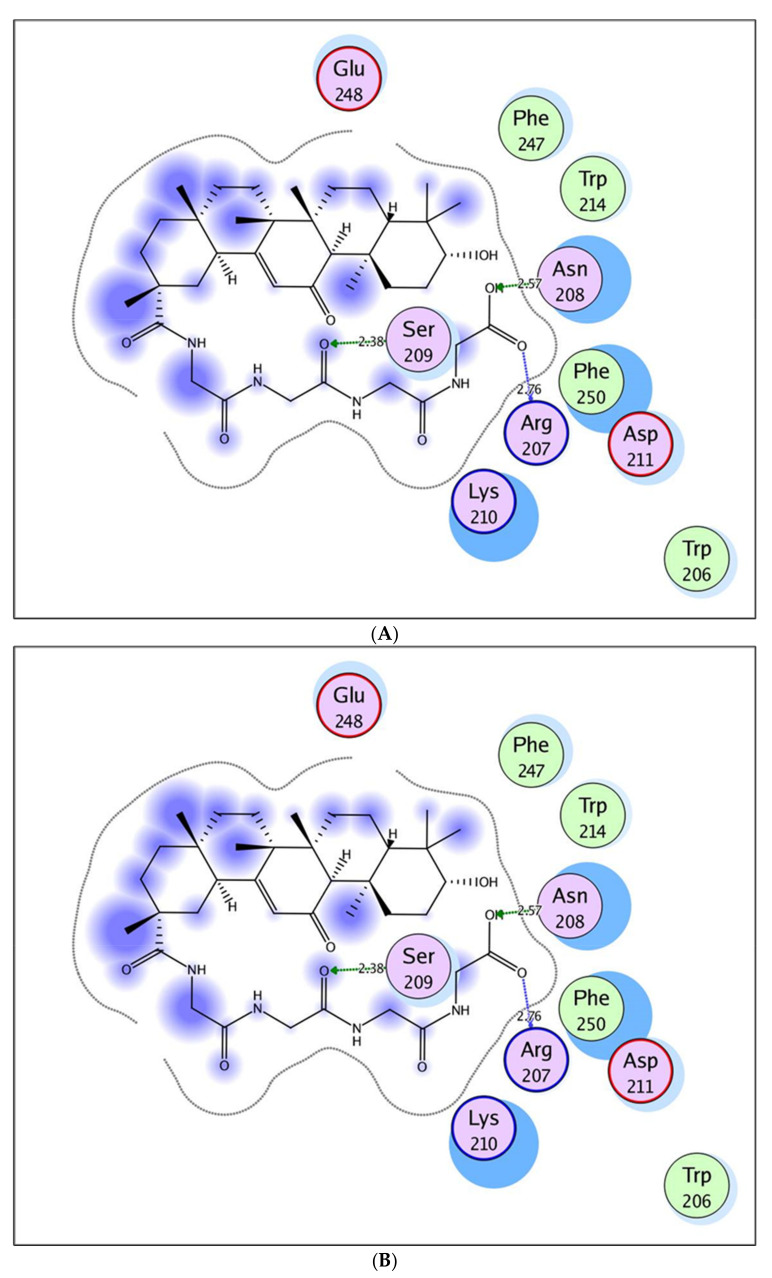
Maps (**A**) and (**B**) explore 2D and 3D binding modes of the promising target 5 within the active site of caspase-3 (PDB: 2J30).

The docking of compound **5** within the ATP-pocket of caspase-3 is depicted in Figure 6. It could be seen that the two oxygens of the terminal carboxylic group interacted with the backbone of Arg207 and the side chain of Asn208 and generated two hydrogen bonds (distance: 2.76 and 2.57 Å, respectively). Additionally, the amide oxygen formed the H-bond acceptor with the side chain of Ser209 (distance: 2.38 Å). The whole compound was enclosed within the active site through hydrophobic interactions with the essential amino acids Trp206, Lys210, Asp211, Trp214, Glu248, and Phe250.

**Figure 7:**
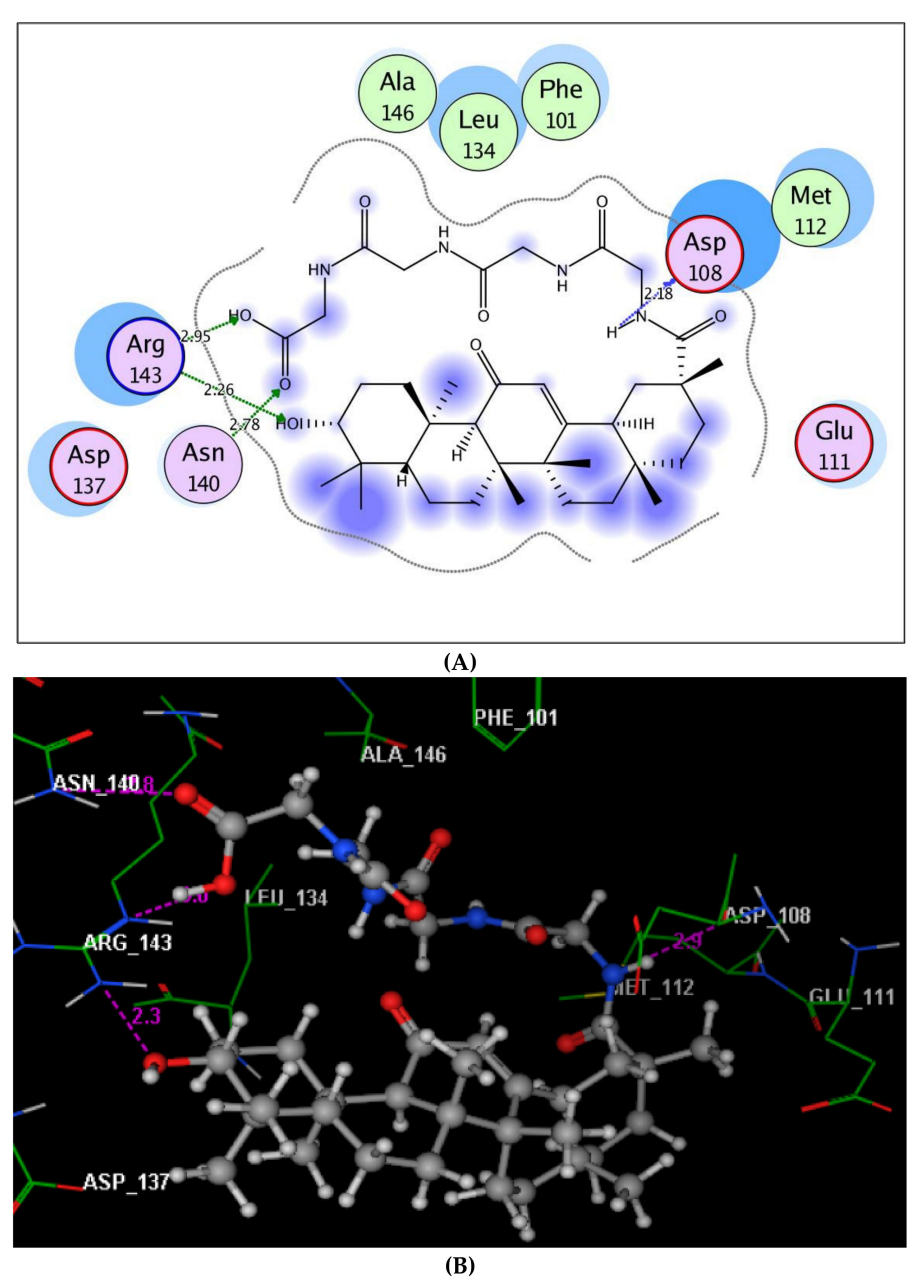
Maps (**A**) and (**B**) explore 2D and 3D binding modes of the promising target 5 within Table 2 (PDB: 4LVT).

Regarding to the Bcl-2 active site (Figure 7), the carboxylic group embedded nicely through two H-bond acceptors between its two oxygens and the sidechains of Asn140 and Arg143 (distance: 2.78 and 2.95 Å, respectively). Additionally, the hydroxyl oxygen linked to the side chain of Arg143through the hydrogen bond acceptor (distance: 2.26 Å). The amide proton exhibited the hydrogen bond donor with the backbone of Asp108 (distance: 2.18 Å).

It could be concluded that the presence of the terminal carboxylic group improved the fitting of compound **5** comfortably into the active sites of caspase-3 and Bcl-2 proteins through H-bond interactions. Moreover, the whole structure of compound **5** was located perfectly via hydrophobic interactions with their surrounding residues.

## 3. Materials and Methods

### 3.1. Chemistry

The chemicals, solvents, and thin layer chromatography employed in this work were taken from international chemical companies: E. Merck (Hohenbrunn, Germany), Fluka (Buchs, Switzerland), and Sigma (Ronkonkoma, NY, USA). The melting points are uncorrected and were estimated through the digital electro-thermal melting point apparatus using opened glass capillary tubes. The elemental micro-analyses were given within good limits of the theoretical values (±0.4%) for carbon, nitrogen, and hydrogen at Micro-Analytical Unit, Cairo University, Cairo, Egypt. The infrared (IR) spectra were recorded as KBr disks at the Micro-Analytical Unit at Cairo University in Egypt through the Fourier transform infrared spectrophotometer (Shimadzu; Model: IR affinity-1S). The mass spectra measurements occurred at the Micro-Analytical Unit at Cairo University in Egypt on a gas chromategraph mass spectrometer (Shimadzu, Kyoto, Japan; Model: QP2010ultra). The^1^H-NMR spectra were operated on JEOL, JöEL500 MHz instruments (Tokyo, Japan) in DMSO-*d_6_*.

#### 3.1.1. Synthesis of Amino Acid Ester Hydrochlorides and Boc-Protected Amino Acids

These derivatives were prepared according to the general method described previously [48].

#### 3.1.2. Synthesis of OH-GA-Gly-Gly-OEt; Ethyl 2-(2-(10-Hydroxy-2,4a,6a,6b,9,9,12a-heptamethyl-13-oxo-1,2,3,4,4a,5,6,6a,6b,7,8,8a,9,10,11,12,12a,12b,13,14b-icosahydropicene-2-carboxamido)acetamido)acetate, (1)

Ethyl chloroformate (ECF) (2.9 mL, 30.1 mmol) was added to a stirred and a cold DCM solution (−20 °C, 50 mL) solution of glycyrrhetinic acid (**A**), (5 g, ~30 mmol) and triethylamine (TEA) (6.6 mL, 60.2 mmol). The reaction mixture was stirred for an additional 30 min, and then, a DCM solution (−20 °C, 50 mL) of free Gly-Gly-ethyl ester (11g; 60.2 mmol) was added. Stirring was maintained for 3 h at −20 °C, then for 24 h at room temperature. The reaction mixture was then washed with water, 1N sodium bicarbonate, 1N potassium hydrogen sulphate, and water and, finally, dried over anhydrous sodium sulphate, the volatile materials were evaporated until dryness, and the obtained oily residue was solidified by trituration with pet. ether (B.P. 40–60 °C). The obtained solid was collected by filtration and recrystallized from EtOH to give compound **1**.

(**1**); yield: 60%; m.p. 160–162 °C.IR (cm^−1^): (KBr): 3650 (OH primary cyclic alcohol), 3408 (NH stretching), 2933 (CH aliphatic), 1752 (C=O ester), 1659, 1535, and 1457 (C=O amide I, amide II, and amid III), 1382, 1334 (CH_2_ aliphatic), 1252, 1195 (CH_3_ aliphatic).^1^H-NMR (500 MHz, δ, ppm, DMSO-*d_6_*): δ: 9.04–8.80 (s, 2H, CON**H**, D_2_O exchangeable), 5.51 (s, 1H, COC**H**=C, GL moiety), 5.00 (s, 1H, OH, GL moiety), 4.61–4.33 (s, 4H, 2CH_2_, and q, 2H, OC**H_2_**CH_3_, Gly), 2.85–2.45 (t, 4H, and 3CH and s, CH, and GL moiety), 1.94–1.72 (16H, t, 6CH_2_, m, CH_2_, d, CH_2_, and GL moiety), 1.44 (t, 3H, OCH_2_C**H_3_**,andGly), 1.05–0.80 ppm(s, 21H, 7CH_3_, and GL moiety). MS (EI, 70 eV): *m*/*z* (%) =613 (M^+^, 36.36%), 612 (60.84%), 610 (44.06%), 548 (82.52%), 428 (86.71%), 135 (90.21%),71** (100%**), and 50 (48.95%). Molecular formula (M.wt.): C_36_H_56_N_2_O_6_ (612.8). Calculated analysis: C, 70.55; H, 9.21; N, 4.57, found: C, 70.50; H, 9.14; N, 4.51 (see Supplementary Materials).

#### 3.1.3. Synthesis of Boc-l-Leu-N-Val-GA-GLy-Gly-Oet; 11-(2-(2-Ethoxy-2-oxoethylamino)-2-oxoethylcarbamoyl)-4,4,6a,6b,8a,11,14b-heptamethyl-14-oxo-1,2,3,4,4a,5,6,6a,6b,7,8,8a,9,10,11,12,12a,14,14a,14b-icosahydropicen-3-yl 2-(2-(tert-Butoxycarbonylamino)-4-methylpentanamido)pentanoate, (2)

Ethyl chloroformate (ECF) (2.9 mL, 30.1 mmol) was added to a stirred and a cold DCM solution (−20 °C, 50 mL) solution of Boc-l-Leu-*N*-Val (~30 mmol) and triethylamine (TEA) (6.6 mL, 60.2 mmol). The reaction mixture was stirred for an additional 30 min, and then, a DCM solution (−20 °C, 50 mL) of compound **1** (60.2 mmol) was added. The work up was continued as followed for compound **1**. The obtained solid was filtered off and recrystallized from EtOH to give the ester **2**.

(**2**); yield: 65%; m.p. 169–171 °C [α]${}_{D}^{25}$:−319.77 (C, 0.02, and DMSO). IR (cm^−1^): (KBr): 3375 (NH stretching), 2934 (CH aliphatic), 1743 (C=O ester), 1655, 1530, and 1458 (C=O amide I, amide II, and amide III), 1383 (CH_2_ aliphatic), and 1200 (CH_3_ aliphatic).^1^H-NMR (500 MHz, δ, ppm, DMSO-*d_6_*): δ: 9.04, 9.00, 8.45, 7.55 (s, 4H, CON**H**, and D_2_O exchangeable), 5.44 (s, 1H, COC**H**=C, and GL moiety), 4.65 (q, 1H, C**H**NH, and L-Leu), 4.52 (t, 1H, C**H**NH, and L-Nva), 4.25–4.14 (s, 6H, and 2CH_2_ and q, OC**H_2_**CH_3_, and Gly), 3.00–2.25 (t, 4H, and 3CH and s, CH, and GL moiety), 1.90 (q, 2H, CH_2_C**H_2_**CH, and L-Nva), 1.80 (t, 2H, CH_2_, and L-Leu), 1.74–1.60 (16H, t, 6CH_2_, m, CH_2_, d, CH_2_, and GL moiety), 1.50 (t, 1H, CH, and L-Leu), 1.45–1.40 (s, 9H, 3 CH_3_, and Boc moiety), 1.37 (sextet, 2H, C**H_2_**CH_3_, and L-Nva), 1.29 (t, 3H, OCH_2_C**H_3_**, and Gly), 0.95–1.15 (s, 21H, 7CH_3_, and GL moiety), and 0.80–0.90 ppm(d, 6H, CH_3_, and L-Leu). MS (EI, 70 eV): *m*/*z* (%) =925 (M^+^, 0.85%), **613 (100%)**, 597 (20.23%), and 50 (1.44%). Molecular formula (M.wt.): C_52_H_84_N_4_O_10_ (925.2). Calculated analysis: C, 67.50; H, 9.15; N, 6.06, found: C, 67.45; H, 9.10; N, 6.04.

#### 3.1.4. Synthesis of OH-GA-Gly-Gly-COOH; 2-(2-(10-Hydroxy-2,4a,6a,6b,9,9,12a-heptamethyl-13-oxo-1,2,3,4,4a,5,6,6a,6b,7,8,8a,9,10,11,12,12a,12b,13,14b-icosahydropicene-2-carboxamido)acetamido)acetic acid; (3)

To a stirred and cold methanolic solution (−5 °C, 20 mL) of ester **1** (2 mmol), sodium hydroxide (1N, 25 mL) was added drop-wisely. The reaction mixture was stirred for 4 h at the same temperature then for 24 h at room temperature. The solvent was distilled off under reduced pressure, and the remaining aqueous solution was cooled and acidified with 1N hydrochloric acid (pH ∼ 3). The obtained solid was filtered off, washed with water, dried, and recrystallized from EtOH to give the acid **3**.

(**3**); yield: 80%; m.p. 207–209 °C. IR (cm^−1^): (KBr): 3610 (OH primary cyclic alcohol), 3509 (OH acid), 3429 (NH stretching), 2943, 2869 (CH aliphatic), 1705 (C=O acid), 1655, 1531, and 1458 (C=O amide I, amide II, and amid III), 1386, 1326 (CH_2_ aliphatic), 1283, 1256, and 1212 (CH_3_ aliphatic).^1^H-NMR (500 MHz, δ, ppm, DMSO-*d_6_*): δ: 12.47 (s, 1H, OH, Gly), 8.95–8.70 (s, 2H, CON**H**, D_2_O exchangeable), 5.40 (s, 1H, COC**H**=C, GL moiety), 5.05 (s, 1H, OH, GL moiety), 4.35–4.20 (s, 4H, 2CH_2_, Gly), 3.05–2.75 (t, 4H, and 3CH and s, CH, and GL moiety), 1.90–1.75 (16H, t, 6CH_2_, m, CH_2_, d, CH_2_, and GL moiety), 1.15–0.90 ppm (s, 21H, 7CH_3_, and GL moiety).MS (EI, 70 eV): *m*/*z* (%) = 586 (M^+^ + 1, 34.21%), 585 (M^+^, 34.21%), 584 (72.63%), 583 (32.63%), **479 (100%)**, 233 (66.32%), 172 (78.95%), and 50 (46.84%). Molecular formula (M.wt.), C_34_H_52_N_2_O_6_ (584.8). Calculated analysis: C, 69.83; H, 8.96; N, 4.79, found: C, 69.81; H, 8.92; N, 4.75.

#### 3.1.5. Synthesis of OH-GA-Gly-Gly- Gly-Gly-OEt; Ethyl 1-(10-Hydroxy-2,4a,6a,6b,9,9,12a-heptamethyl-13-oxo-1,2,3,4,4a,5,6,6a,6b,7,8,8a,9,10,11,12,12a,12b,13,14b-icosahydropicen-2-yl)-1,4,7,10-tetraoxo-2,5,8,11-tetraazatridecan-13-oate; (4)

Ethyl chloroformate (ECF) (2.9 mL, 30.1 mmol) was added to a stirred and a cold DCM solution (−20 °C, 50 mL) solution of acid **3**, (~30 mmol) and triethylamine (TEA) (6.6 mL, 60.2 mmol). The reaction mixture was stirred for additional 30 min, and then, a DCM solution (−20 °C, 50 mL) of free Gly-Gly-ethyl ester (60.2 mmol) was added. The work up was continued as followed for compound **1**. The obtained solid was filtered off and recrystallized from EtOH to give the ester **4**.

(**4**); yield: 82%; m.p. 130–133 °C IR (cm^−1^): (KBr): 3605 (OH primary cyclic alcohol), 3402, 3310 (NH stretching), 2934, 2868 (CH aliphatic), 1752 (C=O ester), 1660, 1535, and 1457 (C=O amide I, amide II, and amide III), 1383, 1334 (CH_2_ aliphatic), 1252, and 1217 (CH_3_ aliphatic).^1^H-NMR (500 MHz, δ, ppm, DMSO-*d_6_*): δ: 9.10–8.95 (s, 4H, CON**H**, D_2_O exchangeable), 5.60 (s, 1H, COC**H**=C, GL moiety), 4.95 (s, 1H, OH, GL moiety), 4.40–4.25 (s, 10H, and 4CH_2_ and q, OC**H_2_**CH_3_, and Gly), 2.90–2.30 (t, 4H, and 3CH and s, CH, and GL moiety), 1.80–1.65 (16H, t, 6CH_2_, m, CH_2_, d, CH_2_, and GL moiety), 1.35 (t, 3H, OCH_2_C**H_3_**, Gly), 0.95–0.80 ppm (s, 21H, 7CH_3_, and GL moiety).MS (EI, 70 eV): *m*/*z* (%) = 727 (M^+^, 0.21%), 614 (43.57%), **613 (100%)**, 363 (0.44%), 281 (0.41%), and 50 (0.22%). Molecular formula (M.wt.), C_40_H_62_N_4_O_8_ (726.9). Calculated analysis: C, 66.09; H, 8.60; N, 7.71, found: C, 66.11; H, 8.57; N, 7.65.

#### 3.1.6. Synthesis of OH-GA-Gly-Gly-Gly-Gly–COOH; 1-(10-Hydroxy-2,4a,6a,6b,9,9,12a-heptamethyl-13-oxo-1,2,3,4,4a,5,6,6a,6b,7,8,8a,9,10,11,12,12a,12b,13,14b-icosahydropicen-2-yl)-1,4,7,10-tetraoxo-2,5,8,11-tetraazatridecan-13-oic Acid; (5)

To a stirred and cold ethanolic solution (−5 °C, 20 mL) of ester **4** (2 mmol), sodium hydroxide (1N, 25 mL) was added drop-wisely. The reaction mixture was stirred for 4 h at the same temperature then for 24 h at room temperature. The work up was continued as followed for compound **1**. The obtained solid was filtered off and recrystallized from EtOH to give the correspondingacid**5**.

(**5**); yield: 90%; m.p. 292–294 °C IR (cm^−1^): (KBr): 3620 (OH primary cyclic alcohol), 3550 (OH acid), 3424, 3330 (NH stretching), 2930, 2862 (CH aliphatic), 1707 (C=O acid), 1650, 1577, 1533, and 1456 (C=O amide I, amide II, amide III, and amide IV), 1385, 1320 (CH_2_ aliphatic), 1256, and 1210 (CH_3_ aliphatic).^1^H-NMR (500 MHz, δ, ppm, and DMSO-*d_6_*): δ: 12.55 (s, 1H, OH, and Gly), 9.00–8.80 (s, 4H, CON**H**, and D_2_O exchangeable), 5.39 (s, 1H, COC**H**=C, and GL moiety), 5.10 (s, 1H, OH, and GL moiety), 4.50–4.30 (s, 8H, 4CH_2_, and Gly), 2.90–2.50 (t, 4H, and 3CH and s, CH, and GL moiety), 1.85–1.67 (16H, t, 6CH_2_, m, CH_2_, d, CH_2_, and GL moiety), 1.05–0.95 ppm(s, 21H, 7CH_3_, and GL moiety).MS (EI, 70 eV): *m*/*z* (%) = 699 (M^+^, 76.61%), 698 (82.26%), 690 (63.71%), 582 (98.39%), **366 (100%)**, 272 (74.19%), 154 (89.52%), and 54 (44.35%). Molecular formula (M.wt.), C_38_H_58_N_4_O_8_ (698.9). Calculated analysis: C, 65.30; H, 8.36; N, 8.02, found: C, 65.27; H, 8.33; N, 8.00.

#### 3.1.7. Synthesis of OH-GLA-GLy-Gly-l-Leu-Ome(Sh-12);Methyl 2-(2-(2-(10-Hydroxy-2,4a,6a,6b,9,9,12a-heptamethyl-13-oxo-1,2,3,4,4a,5,6,6a,6b,7,8,8a,9,10,11,12,12a,12b,13,14b-icosahydropicene-2-carboxamido)acetamido)acetamido)-4-methylpentanoate; (6)

Ethyl chloroformate (ECF) (2.9 mL, 30.1 mmol) was added to a stirred and a cold DCM solution (−20 °C, 50 mL) solution of acid **3** (~30 mmol) and triethylamine (TEA) (6.6 mL, 60.2 mmol). The reaction mixture was stirred for additional 30 min, and then, a DCM solution (−20 °C, 50 mL) of free L-Leu-methyl ester (60.2 mmol) was added. The work up was continued as followed for compound **1**. The obtained solid was filtered off and recrystallized from EtOH to give the ester **6**.

(**6**); yield: 85%; m.p. 170–172 °C [α]${}_{D}^{25}$:+ 82.25 (C, 0.02, ethanol) IR (cm^−1^): (KBr): 3595 (OH primary cyclic alcohol), 3400 (NH stretching), 2944, 2920 (CH aliphatic), 1695 (C=O ester), 1600, 1510, and 1435 (C=O amide I, amide II, and amide III), 1388 (CH_2_ aliphatic), 1236, and 1210 (CH_3_ aliphatic). ^1^H-NMR (500 MHz, δ, ppm, and DMSO-*d_6_*): δ: 9.15–9.00 (s, 3H, CON**H**, and D_2_O exchangeable), 5.52 (s, 1H, COC**H**=C, and GL moiety), 5.12 (s, 1H, OH, and GL moiety), 4.82 (q, 1H, C**H**NH, and L-Leu), 4.44–4.22 (s, 4H, 2CH_2_, Gly), 3.70 (s, 3H, OCH_3_, and L-Leu), 2.85–2.35 (t, 4H, and 3CH and s, CH, and GL moiety), 1.95 (t, 2H, CH_2_, and L-Leu), 1.85–1.65 (16H, t, 6CH_2_, m, CH_2_, d, CH_2_, and GL moiety), 1.55 (t, 1H, CH, and L-Leu), 1.35–1.15 (s, 21H, 7CH_3_, and GL moiety), 1.05–0.85 ppm(d, 6H, CH_3_, and L-Leu). MS (EI, 70 eV): *m*/*z* (%) =713 (M^+^ + 1, 2.66%), 712 (M^+^, 12.00%), **613 (100%)**, 510 (44.50%), 320 (25.40%), 261 (22.49%), and 50 (3.02%). Molecular formula (M.wt.), C_41_H_65_N_3_O_7_ (712.0). Calculated analysis: C, 69.17; H, 9.20; N, 5.90, found: C, 69.14; H, 9.17; N, 5.85.

#### 3.1.8. Synthesis of OH-GA-Gly-Gly-l-Leu-COOH; 2-(2-(2-(10-Hydroxy-2, 4a, 6a, 6b, 9, 9, 12a-heptamethyl-13-oxo-1, 2, 3, 4, 4a, 5, 6, 6a, 6b, 7, 8, 8a, 9, 10, 11, 12, 12a, 12b, 13, 14b-icosahydropicene-2-carboxamido) acetamido) acetamido)-4-methylpentanoic Acid; (**7**)

To a stirred and cold methanolic solution (−5 °C, 20 mL) of ester **6** (2 mmol), sodium hydroxide (1N, 25 mL) was added drop-wisely. The reaction mixture was stirred for 4 h at the same temperature then for 24 h at room temperature. The work up was continued as followed for compound **1**. The obtained solid was filtered off and recrystallized from EtOH to give the corresponding acid **7**.

(**7**); yield: 66%; m.p. 210–212 °C [α]${}_{D}^{25}$:−216.15 (C, 0.02, DMSO) IR (cm^−1^): (KBr): 3625 (OH primary cyclic alcohol), 3535 (OH acid), 3414 (NH stretching), 2956, 2931 (CH aliphatic), 1744 (C=O acid), 1656, 1532, 1458 (C=O amide I, amide II, and amide III), 1384 (CH_2_ aliphatic), 1253, 1207 (CH_3_ aliphatic).^1^H-NMR (500 MHz, δ, ppm, DMSO-*d_6_*): δ: 12.60 (s, 1H, OH, L-Leu), 9.08–8.97 (s, 3H, CON**H**, and D_2_O exchangeable), 5.48 (s, 1H, COC**H**=C, and GL moiety), 5.02 (s, 1H, OH, and GL moiety), 4.75 (q, 1H, C**H**NH, and L-Leu), 4.38–4.19 (s, 4H, 2CH_2_, and Gly), 2.75–2.25 (t, 4H, and 3CH and s, CH, and GL moiety), 1.90 (t, 2H, CH_2_, and L-Leu), 1.77–1.60 (16H, t, 6CH_2_, m, CH_2_, d, CH_2_, and GL moiety), 1.44 (t, 1H, CH, and L-Leu), 1.25–1.05 (s, 21H, 7CH_3_, and GL moiety), 0.95–0.80 ppm(d, 6H, CH_3_, and L-Leu). MS (EI, 70 eV): *m*/*z* (%) = 698 (M^+^+1, 0.35%), 697 (1.15%), **655 (100%)**, 454 (0.96%), 132 (0.69%), and 52 (0.45%). Molecular formula (M.wt.), C_40_H_63_N_3_O_7_ (697.9). Calculated analysis: C, 68.83; H, 9.10; N, 6.02, found: C, 68.80; H, 9.11; N, 6.01.

#### 3.1.9. Synthesis of Boc-Gly-Gly-GA-Gly-Gly-l-Leu-Ome; Methyl 2-(2-(2-(10-(2-(2-(Tert-butoxycarbonylamino)acetamido)acetoxy)-2,4a,6a,6b,9,9,12a-heptamethyl-13-oxo-1,2,3,4,4a,5,6,6a,6b,7,8,8a,9,10,11,12,12a,12b,13,14b-icosahydropicene-2-carboxamido)acetamido)acetamido)-4-methylpentanoate, (**8**)

Ethyl chloroformate (ECF) (2.9 mL, 30.1 mmol) was added to a stirred and a cold DCM solution (−20 °C, 50 mL) solution of ester**6**, (~30 mmol) and triethylamine (TEA) (6.6 mL, 60.2 mmol). The reaction mixture was stirred for additional 30 min, and then, a DCM solution (−20 °C, 50 mL) of free Boc-Gly-Gly (60.2 mmol) was added. The work up was continued as followed for compound **1**. The obtained solid was filtered off and recrystallized from EtOH to give compound **8**.

(**8**); yield: 70%; m.p. 120–118 °C [α]${}_{D}^{25}$:+48.00 (C, 0.02, DMSO).IR (cm^−1^): (KBr): 3329 (NH stretching), 2931 (CH aliphatic), 1658 (C=O ester), 1532, 1452 (C=O amide I and amide II), 1386, 1372 (CH_2_ aliphatic), 1247, and 1212 (CH_3_ aliphatic).^1^H-NMR (500 MHz, δ, ppm, and DMSO-*d_6_*): δ: 9.22, 9.10, 9.00, 8.00, 7.75 (s, 5H, CON**H**, and D_2_O exchangeable), 5.60 (s, 1H, COC**H**=C, and GL moiety), 4.80 (q, 1H, C**H**NH, and L-Leu), 4.40–4.14 (s, 8H, 4CH_2_, and Gly), 3.11–2.50 (t, 4H, and 3CH and s, CH, and GL moiety), 1.99 (t, 2H, CH_2_, and L-Leu), 1.85–1.70 (16H, t, 6CH_2_, m, CH_2_, d, CH_2_, and GL moiety), 1.55–1.45 (s, 9H, 3 CH_3_, and Boc moiety), 1.40 (t, 1H, CH, and L-Leu), 1.30 (t, 3H, OCH_2_C**H_3_**, and L-Leu), 1.15–0.95 (s, 21H, 7CH_3_, and GL moiety), and 0.90–0.75 ppm(d, 6H, CH_3_, and L-Leu). MS (EI, 70 eV): *m*/*z* (%) =926 (M^+^, 3.88%), 924 (63.29%), 875 (47.99%), **265 (100%)**, 135 (68.10%), and 50 (7.97%). Molecular formula (M.wt.), C_50_H_79_N_5_O_11_ (926.2). Calculated analysis: C, 64.84; H, 8.60; N, 7.56, found: C, 64.80; H, 8.63; N, 7.55.

### 3.2. Biological Activities

#### 3.2.1. Cytotoxicity

Human breast MCF-7, colon HCT-116, and liver HepG-2 carcinoma cell line were taken from Vacsera (Giza, Egypt). The culture was retained in RPMI 1640 medium with 1% antibiotic–antimycotic mixture (25 μg/mL amphotericin B, 10,000 μg/mL streptomycin sulfate, and 10,000 U/mL potassium penicillin), 1% *L*-glutamine, and complemented by 10% heat inactivated fetal bovine serum. The culturing and sub culturing were done using doxorubicin as a positive control and DMSO as a negative control. This experiment was carried out according to the reported MTT assay method, and the absorbance was measured at 595 nm [33,34,35].

#### 3.2.2. Estimation of Bcl-2 Level

The samples and standards having Bcl-2 were estimated as previously mentioned [64]. Addition of biotin-conjugated antibody was followed by streptavidin-HRP. Then, the reaction was terminated by adding acid, and the absorbance was measured at 450 nm.

#### 3.2.3. Estimation of Bax Level

The levels of Bax protein were evaluated according to the reported method [65]. Addition of monoclonal antibody specific to Bax captured on the plate was followed by incubation and addition of streptavidin conjugated to horseradish peroxidase. Then, the reaction was terminated by adding acid, and optical density of the produced color was measured at 450 nm.

#### 3.2.4. Estimation of Human p53 Level

Human p53 present in MCF-7 cells was determined; using Human p53 ELISA-Kit (**CS0070** Sigma) read using spectrophotometer at 450 nm against untreated control cells (negative control) and doxorubicin (positive control) applying the standard protocols of the manufacturers [66]. The samples or standard having human p53 bind to antibodies adsorbed to the microwells. Addition of biotin-conjugated was followed by incubation and addition of dispense of unbound biotin-conjugated streptavidin HRP. Then, the reaction was terminated by adding acid, and the absorbance was measured at 450 nm [54].

#### 3.2.5. Calculation of IC_50_Values

The IC_50_ values were estimated for the promising active extracts using the SPSS computer program (SPSS for windows, statistical analysis software package/version 9/1989 SPSS Inc., Chicago, IL, USA) and utilizing probit analysis.

#### 3.2.6. Human CASP-3(Caspase-3) Estimation

To measure caspase-3 activity, a luminescent Caspase-Glo 7 assay was performed. MCF-7 (p53+/+) and (p53-/-) cells were seeded onto black 96-well plates at a density of 3500 cells/well. After 24 h the compounds and a reference (doxorubicin) at IC_50_ concentration were added. After 48 h, a Caspase-Glo 3 Assay (Promega Corporation 2800 Woods Hollow Road Madison, WI 53711–5399 USA)) was performed according to the manufacturer’s instructions. After adding 100 μL of Caspase 3 Glo Reagent, cells were incubated for 2.5 h at room temperature. The luminescence was measured using the fluorescence omega microplate reader (BMG LabTech, Germany) at 490 nm excitation and 520 nm emission [67].

#### 3.2.7. Human CASP-7 (Caspase-7) Estimation

The micro-ELISA plate provided in this kit pre-coated with CASP-7-specific antibody. A biotinylated CASP7 antibody and Avidin–horseradish p eroxidase (HRP) conjugate was added. Aspire the excess components. The substrate solution was added. Wells that contain CASP-7, biotinylated detection antibody, and Avidin-HRP conjugate will appear blue in color. The color turns yellow followed the addition of sulfuric acid solution. The optical density (OD) was measured at a wavelength of 450 nm ± 2 nm [68].

#### 3.2.8. Enzyme-Linked Immunosorbent Assay Kit for Tubulin Beta (TUBb)

The MCF-7 cells were inoculated using DMEM (supplemented with 1% penicillin-streptomycin and 10% FBS) at concentration 1.2−1.8 × 10,000 cells/well. For 18−24 h before the enzyme assay of Tubulin, the tested compounds were added, followed by the addition of Avidin conjugated to horseradish peroxidase (HRP) to each microplate well and incubated. After addition of TMB substrate solution, the wells containing TUBb, enzyme-conjugated Avidin, and biotin-conjugated antibody revealed a change in color. Addition of sulfuric acid solution terminated the enzymatic reaction. The color change was measured spectrophotometrically at a wavelength of 450 ± 10 nm [69].

#### 3.2.9. Estimation of DNA Fragmentation through DPA Assay

Assessment of DNA fragmentation of the cells was done, as described by the reported method [70,71]. Briefly, the cells were lysed for 15 min on ice with 0.5% (*v*/*v*) Triton X-100, 20 mM EDTA, and 5 uM Tris (pH 8.0). Then, the cells were centrifuged to separate intact chromatin from DNA fragments for 20 min, at 27,000 Xg. Measurement of the amount of DNA was done using a diphenylamine reagent, and the optical density was measured at 600 nm.

Statistical analyses for the previous parameters were done using one-way analysis of variance (ANOVA) using the CoStat computer program accompanied by the least significant level (LSD) between groups at *p* < 0.05. Unshared letters (a, b, and c) are significant values between groups for each parameter at *p* < 0.0001.

#### 3.2.10. In Vitro Antimicrobial Activity

Antimicrobial activity of the tested chemical peptides was determined by the well diffusion method for pathogenic bacteria having Gram-positive bacteria (*Staphylococcus aureus ATCC25923, Streptococcus pneumonia*, and *Micrococcus luteus*) and Gram-negative bacteria (*Escherichia coli ATCC25922*, *Pseudomonas aeruginosa ATCC7853*, and *Proteus sp.*) and *Candida albicans* as human pathogen yeast using the well diffusion method [72,73].

### 3.3. Molecular Docking Study

Molecular docking of derivative 5 against caspase-3 and Bcl-2 was utilized using Molecular Operating Environment (MOE-Dock) software version 2014.0901 [59,60,61] to explore the possible interactions between the target 5 and the two proteins. The crystal structures of caspase-3 (PDB code: 2J30) [62] and Bcl-2 (PDB code: 4LVT) [63] was downloaded from the RCSB Protein Data Bank and prepared for the docking process.

## 4. Conclusions

This study deals with synthesis of new glycyrrhetinic acid (GA)-based peptides via conventional synthetic peptide coupling methods (solution phase) of GA with different amino acids to obtain the new peptides 1–8. All the new compounds were evaluated as cytotoxic candidates against MCF-7, HCT-116, and HepG-2 cell lines through MTT assay. Compounds **3**, **5**, and **7** were more cytotoxic against MCF-7 cell lines with IC_50_ 5.10, 5, 3.70 μg/mL, and against HCT-116 cell lines, with IC_50_ 7.40, 5.2, and 3.0 μg/mL, respectively. Regarding the promising safety study on the normal BJ-1 cell lines, compound 5 was selected among compounds **1** and **3** to analyze its effect against various apoptotic markers in human breast MCF-7 cancerous cells. The upregulation of p53, caspase-7, caspase-3, Bax/Bcl-2 ratio, % of DNA fragmentation as well as tubulin polymerization (TubB) have confirmed the five apoptotic pathways. Furthermore, the new peptides were evaluated as antimicrobial agents against various Gram-negative and Gram-positive bacteria and the yeast candida Albicans. All the tested GA analogues **1**–**8** exhibited more antibacterial effect against Micrococcus Luteus than gentamicin, but they exhibited moderate antimicrobial activity against the tested bacterial and yeast strains. The docking study of ligand 5 with caspase-3 and Bcl-2 proteins represented that **5** produced good fitting into the active pockets of the target proteins with the best binding energies confirming the upregulation of caspase-3 and downregulation of Bcl-2 protein levels. Thus, GA-based peptide 5 could be considered as a suitable template in the anticancer field and deserves further improvement and optimization.

## Figures and Tables

**Table 1:** IC_50_ values of the GA-based peptides against different cancer cell lines.

Compd. No.	IC_50_ (µg/mL) ± SD		
	MCF-7	HCT-116	HepG-2
**2**	7.70 ± 1.3	70.30 ± 0.9	---
**3**	5.1 ± 0.7	7.40 ± 0.4	---
**4**	6.10 ± 0.4	73.0 ± 1.4	---
**5**	5.0 ± 0.3	5.2 ± 0.8	---
**7**	3.70 ± 0.2	3.0 ± 1.1	3.30 ± 0.1
**8**	6.90 ± 1.1	60.70 ± 0.6	---
**DOX**	7.07 ± 1	1.09 ± 3	0.54 ± 1

IC_50_: The concentration of the compound that is necessary to inhibit the cell viability by 50%, each value is the mean of three replicates, SD = standard deviation.

**Table 2:** Antimicrobial activity of the newly synthesized targets using the well diffusion assay method.

Pathogenic Microbial Strains		Diameter of Inhibition Zone (mm)								
		1	2	3	4	5	7	8	GM	KC
**Gram-positive bacteria**	*Streptococcus pneumonia RCMB 010010*	15 ± 0.16	12 ± 0.82	17 ± 0.21	11 ± 0.24	16 ± 0.12	-	12 ± 0.22	27.2 ± 0.80	
	*Staphylococcus aureus ATCC25923*	15 ± 0.16	17 ± 0.11	17 ± 0.32	10 ± 0.13	15 ± 0.15	-	13 ± 0.24	25.4 ± 0.18	
	*Micrococcus Luteus*	30 ± 0.21	30 ± 0.13	30 ± 0.25	29 ± 0.13	30 ± 0.15	30 ± 0.11	30 ± 0.17	24.4 ± 0.72	
**Gram-negative bacteria**	*Escherichia coli ATCC25922*	20 ± 0.25	18 ± 0.14	16 ± 0.31	13 ± 0.15	18 ± 0.23	15 ± 0.21	15 ± 0.16	26.3 ± 0.15	
	*Pseudomonas aeruginosa ATCC7853*	18 ± 0.15	15 ± 0.25	15 ± 0.18	13 ± 0.16	17 ± 0.16	13 ± 0.14	14 ± 0.16	24.41 ± 0.18	
	*Proteus vulgaris RCMB 010085*	-	-	17 ± 0.17	12 ± 0.23	10 ± 0.17	12 ± 0.14	-	20.0 ± 0.30	
**Yeast**	*Candida albicans*	-	-	12 ± 0.23	15 ± 0.12	13 ± 0.17	-	-		22.8 ± 0.10

GM: gentamicin; CK: ketoconazole.

## Data Availability

Not applicable.

## Supplementary Materials

The Supplementary materials are available online.

## Sample Availability

Samples of the compounds (**1**–**8**) are available from the authors.
