# Supplementary material for: Synthesis, Characterization, In Vitro Anticancer Potentiality, and Antimicrobial Activities of Novel Peptide–Glycyrrhetinic-Acid-Based Derivatives
Source: Molecules. 2021 Jul 28;26(15):4573. doi: 10.3390/molecules26154573 (PMC8346995; doi:10.3390/molecules26154573)
Supplement: Supplementary file 1 [file molecules-26-04573-s001.zip › molecules-1283026-supplementary.pdf]

# Peak Find - Memory-159

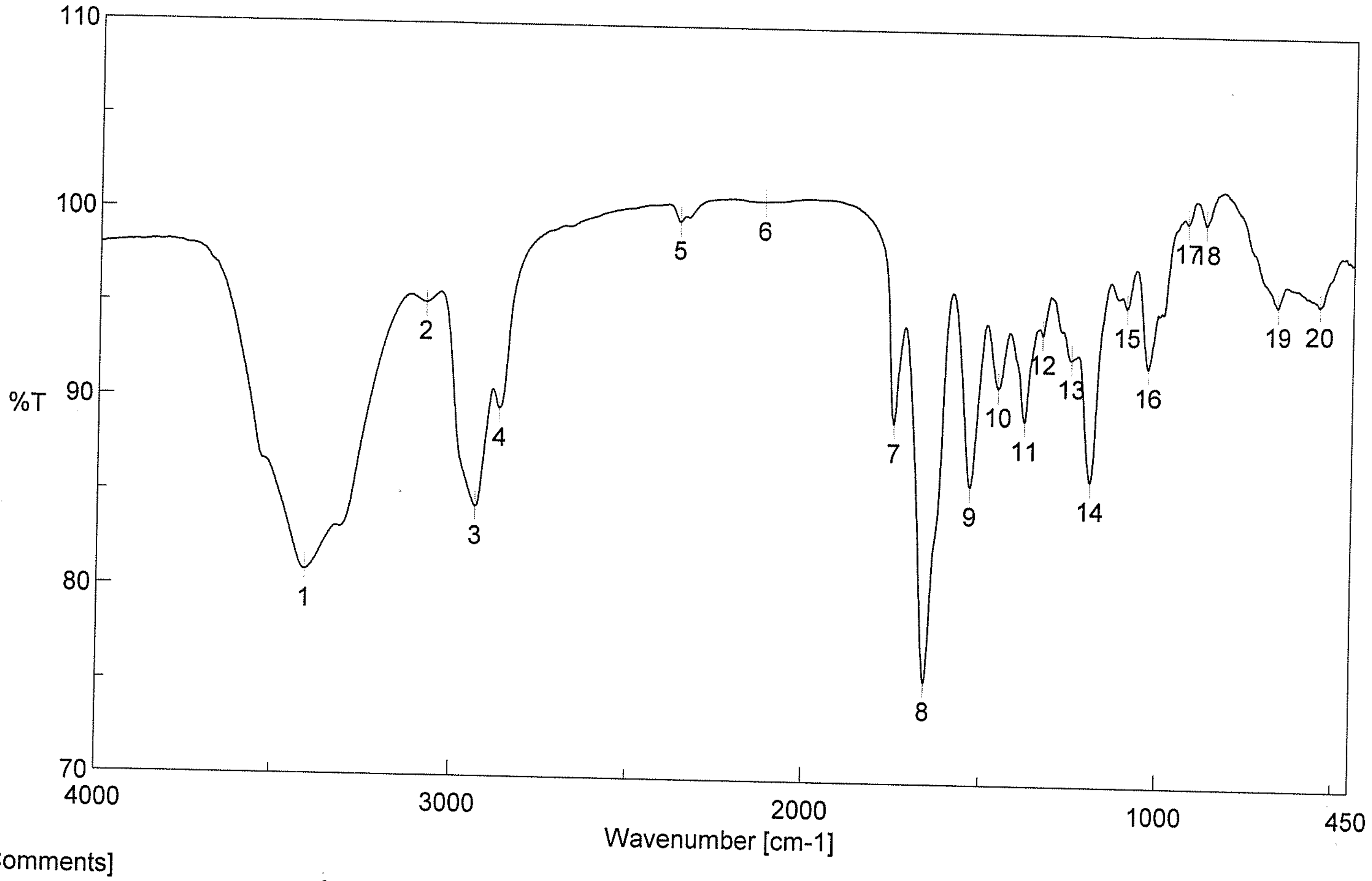


[Comments]

| | |
|---|---|
| Sample name | SH-5 = 1 |
| Comment | 24/1/2017 |
| User | IR |
| Division | IR |
| Company | MAC |

[ Result of Peak Picking ]

| No. | Position | Intensity | No. | Position | Intensity | No. | Position | Intensity |
|---|---|---|---|---|---|---|---|---|
| 1 | 3408.57 | 80.8431 | 2 | 3077.83 | 95.0902 | 3 | 2933.2 | 84.2776 |
| 4 | 2868.59 | 89.4869 | 5 | 2362.37 | 99.5843 | 6 | 2121.31 | 100.718 |
| 7 | 1752.01 | 89.0348 | 8 | 1659.45 | 75.4322 | 9 | 1535.06 | 85.7779 |
| 10 | 1457.92 | 91.052 | 11 | 1382.71 | 89.285 | 12 | 1334.5 | 93.9198 |
| 13 | 1252.54 | 92.6249 | 14 | 1195.65 | 86.168 | 15 | 1095.37 | 95.4245 |
| 16 | 1032.69 | 92.2318 | 17 | 924.7 | 99.9888 | 18 | 872.631 | 99.938 |
| 19 | 667.25 | 95.6271 | 20 | 547.685 | 95.6957 | | | |

# Cairo University
# Micro Analytical Center

## *DI Analysis*
## *Shimadzu Qp-2010 Plus*

Sample Information

Analyzed by : Dr. Mai Younis
Analyzed : 29/01/2017 11:30:49 ص
Sample Name : Sh-5 ش ١
Sample ID :
Customer Name : Dr. Gaber Osman - NRC
Data File : C:\GCMSsolution\Data\Project1\Sh-5.QGD
Org Data File : C:\GCMSsolution\Data\Project1\Sh-5.QGD
Method File : C:\GCMSsolution\Data\Project1\High Temperature Op
Org Method File : C:\GCMSsolution\Data\Project1\High Temperature Op
Report File :
Tuning File : C:\GCMSsolution\System\Tune1_default.qgt
$EndIf$Modified by : Dr. Mai Younis
Modified : 29/01/2017 11:36:57 ص

Method

===== Analytical Line 1 =====
IonSourceTemp :250.00 °C
[MS Table]
--Group 1 - Event 1--
Start Time :0.00min
End Time :10.00min
ACQ Mode :Scan
Event Time :0.50sec
Scan Speed :1428
Start m/z :50.00
End m/z :700.00

Electron Voltage : 70 eV
Ionization Mode : EI

C:\GCMSsolution\Data\Project1\Sh-5.QGD

Chromatogram Sh-5 C:\GCMSsolution\Data\Project1\Sh-5.QGD

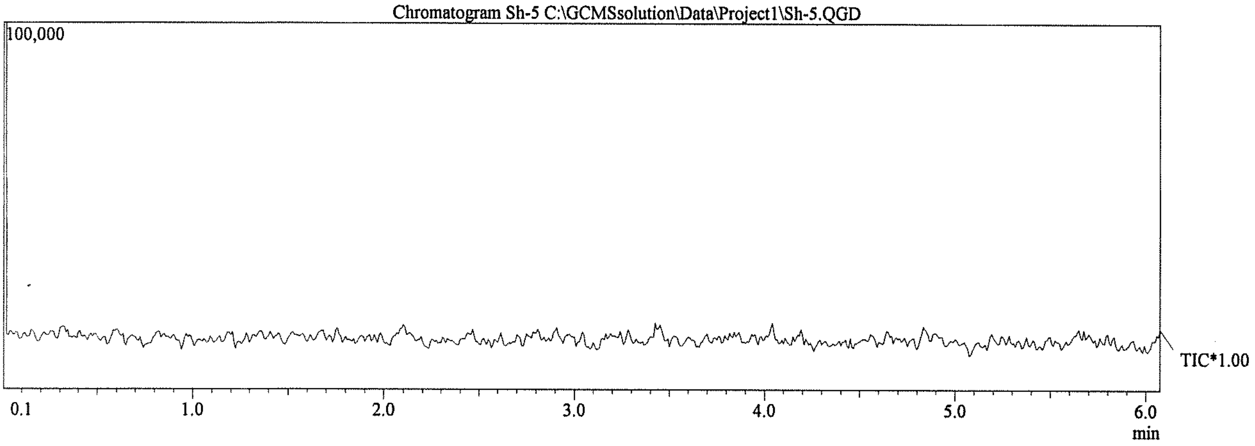


Spectrum

Line#:1 R.Time:3.5(Scan#:419)
MassPeaks:166
RawMode:Single 3.5(419) BasePeak:71(143)
BG Mode:None Group 1 - Event 1

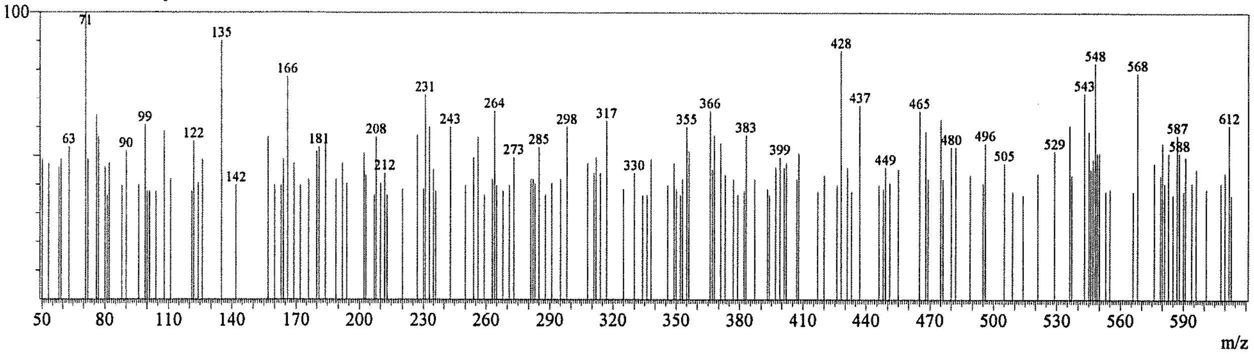


Mass Table
Line#:1 R.Time:3.5(Scan#:419)
MassPeaks:166
RawMode:Single 3.5(419) BasePeak:71(143)
BG Mode:None Group 1 - Event 1

| # | m/z | Abs. In | Rel. Int. | # | m/z | Abs. In | Rel. Int. | # | m/z | Abs. In | Rel. Int. |
|---|---|---|---|---|---|---|---|---|---|---|---|
| 1 | 50.00 | 70 | 48.95 | 4 | 59.00 | 70 | 48.95 | 7 | 72.00 | 70 | 48.95 |
| 2 | 53.00 | 68 | 47.55 | 5 | 63.00 | 76 | 53.15 | 8 | 76.00 | 92 | 64.34 |
| 3 | 58.00 | 66 | 46.15 | 6 | 71.00 | 143 | 100.00 | 9 | 77.00 | 81 | 56.64 |

| # | m/z | Abs. In | Rel. Int. |
|---|---|---|---|
| 10 | 80.00 | 66 | 46.15 |
| 11 | 81.00 | 52 | 36.36 |
| 12 | 82.00 | 68 | 47.55 |
| 13 | 88.00 | 57 | 39.86 |
| 14 | 90.00 | 74 | 51.75 |
| 15 | 96.00 | 57 | 39.86 |
| 16 | 99.00 | 87 | 60.84 |
| 17 | 100.00 | 54 | 37.76 |
| 18 | 101.00 | 54 | 37.76 |
| 19 | 104.00 | 54 | 37.76 |
| 20 | 108.00 | 84 | 58.74 |
| 21 | 111.00 | 60 | 41.96 |
| 22 | 121.00 | 54 | 37.76 |
| 23 | 122.00 | 79 | 55.24 |
| 24 | 124.00 | 58 | 40.56 |
| 25 | 126.00 | 70 | 48.95 |
| 26 | 135.00 | 129 | 90.21 |
| 27 | 142.00 | 57 | 39.86 |
| 28 | 157.00 | 81 | 56.64 |
| 29 | 160.00 | 57 | 39.86 |
| 30 | 163.00 | 57 | 39.86 |
| 31 | 164.00 | 70 | 48.95 |
| 32 | 166.00 | 111 | 77.62 |
| 33 | 169.00 | 68 | 47.55 |
| 34 | 172.00 | 57 | 39.86 |
| 35 | 176.00 | 60 | 41.96 |
| 36 | 180.00 | 74 | 51.75 |
| 37 | 181.00 | 76 | 53.15 |
| 38 | 184.00 | 79 | 55.24 |
| 39 | 189.00 | 60 | 41.96 |
| 40 | 192.00 | 68 | 47.55 |
| 41 | 194.00 | 58 | 40.56 |
| 42 | 202.00 | 73 | 51.05 |
| 43 | 203.00 | 62 | 43.36 |
| 44 | 207.00 | 52 | 36.36 |
| 45 | 208.00 | 81 | 56.64 |
| 46 | 210.00 | 58 | 40.56 |
| 47 | 212.00 | 63 | 44.06 |
| 48 | 213.00 | 52 | 36.36 |
| 49 | 220.00 | 55 | 38.46 |
| 50 | 227.00 | 82 | 57.34 |
| 51 | 230.00 | 55 | 38.46 |
| 52 | 231.00 | 102 | 71.33 |
| 53 | 233.00 | 86 | 60.14 |
| 54 | 235.00 | 65 | 45.45 |
| 55 | 236.00 | 54 | 37.76 |
| 56 | 243.00 | 86 | 60.14 |
| 57 | 250.00 | 57 | 39.86 |
| 58 | 254.00 | 71 | 49.65 |
| 59 | 256.00 | 81 | 56.64 |
| 60 | 259.00 | 52 | 36.36 |
| 61 | 263.00 | 60 | 41.96 |
| 62 | 264.00 | 94 | 65.73 |
| 63 | 265.00 | 57 | 39.86 |
| 64 | 268.00 | 54 | 37.76 |
| 65 | 271.00 | 57 | 39.86 |
| 66 | 273.00 | 71 | 49.65 |
| 67 | 281.00 | 60 | 41.96 |
| 68 | 282.00 | 60 | 41.96 |
| 69 | 283.00 | 58 | 40.56 |
| 70 | 285.00 | 76 | 53.15 |
| 71 | 288.00 | 52 | 36.36 |
| 72 | 291.00 | 58 | 40.56 |
| 73 | 295.00 | 60 | 41.96 |
| 74 | 298.00 | 86 | 60.14 |
| 75 | 308.00 | 68 | 47.55 |
| 76 | 311.00 | 63 | 44.06 |
| 77 | 312.00 | 71 | 49.65 |
| 78 | 314.00 | 63 | 44.06 |
| 79 | 317.00 | 89 | 62.24 |
| 80 | 325.00 | 55 | 38.46 |
| 81 | 330.00 | 63 | 44.06 |
| 82 | 334.00 | 52 | 36.36 |
| 83 | 336.00 | 52 | 36.36 |
| 84 | 338.00 | 70 | 48.95 |
| 85 | 346.00 | 57 | 39.86 |
| 86 | 349.00 | 68 | 47.55 |
| 87 | 350.00 | 55 | 38.46 |
| 88 | 352.00 | 52 | 36.36 |
| 89 | 353.00 | 60 | 41.96 |
| 90 | 355.00 | 86 | 60.14 |
| 91 | 356.00 | 74 | 51.75 |
| 92 | 366.00 | 94 | 65.73 |
| 93 | 367.00 | 65 | 45.45 |
| 94 | 368.00 | 82 | 57.34 |
| 95 | 371.00 | 78 | 54.55 |
| 96 | 373.00 | 62 | 43.36 |
| 97 | 377.00 | 60 | 41.96 |
| 98 | 379.00 | 52 | 36.36 |
| 99 | 382.00 | 54 | 37.76 |
| 100 | 383.00 | 82 | 57.34 |
| 101 | 387.00 | 60 | 41.96 |
| 102 | 393.00 | 55 | 38.46 |
| 103 | 394.00 | 52 | 36.36 |
| 104 | 397.00 | 66 | 46.15 |
| 105 | 399.00 | 71 | 49.65 |
| 106 | 401.00 | 66 | 46.15 |
| 107 | 402.00 | 68 | 47.55 |
| 108 | 407.00 | 60 | 41.96 |
| 109 | 408.00 | 73 | 51.05 |
| 110 | 417.00 | 54 | 37.76 |
| 111 | 420.00 | 62 | 43.36 |
| 112 | 426.00 | 57 | 39.86 |
| 113 | 428.00 | 124 | 86.71 |
| 114 | 431.00 | 66 | 46.15 |
| 115 | 433.00 | 54 | 37.76 |
| 116 | 437.00 | 97 | 67.83 |
| 117 | 446.00 | 57 | 39.86 |
| 118 | 448.00 | 55 | 38.46 |
| 119 | 449.00 | 66 | 46.15 |
| 120 | 451.00 | 58 | 40.56 |
| 121 | 455.00 | 65 | 45.45 |
| 122 | 465.00 | 94 | 65.73 |
| 123 | 468.00 | 84 | 58.74 |
| 124 | 469.00 | 60 | 41.96 |
| 125 | 475.00 | 90 | 62.94 |
| 126 | 476.00 | 60 | 41.96 |
| 127 | 480.00 | 76 | 53.15 |
| 128 | 482.00 | 76 | 53.15 |
| 129 | 489.00 | 62 | 43.36 |
| 130 | 495.00 | 58 | 40.56 |
| 131 | 496.00 | 78 | 54.55 |
| 132 | 505.00 | 68 | 47.55 |
| 133 | 509.00 | 54 | 37.76 |
| 134 | 514.00 | 52 | 36.36 |
| 135 | 521.00 | 63 | 44.06 |
| 136 | 529.00 | 74 | 51.75 |
| 137 | 536.00 | 87 | 60.84 |
| 138 | 537.00 | 62 | 43.36 |
| 139 | 543.00 | 103 | 72.03 |
| 140 | 545.00 | 84 | 58.74 |
| 141 | 546.00 | 65 | 45.45 |
| 142 | 547.00 | 70 | 48.95 |
| 143 | 548.00 | 118 | 82.52 |
| 144 | 549.00 | 73 | 51.05 |
| 145 | 550.00 | 73 | 51.05 |
| 146 | 553.00 | 54 | 37.76 |
| 147 | 555.00 | 55 | 38.46 |
| 148 | 566.00 | 54 | 37.76 |
| 149 | 568.00 | 113 | 79.02 |
| 150 | 576.00 | 68 | 47.55 |
| 151 | 579.00 | 62 | 43.36 |
| 152 | 580.00 | 78 | 54.55 |
| 153 | 581.00 | 58 | 40.56 |
| 154 | 583.00 | 73 | 51.05 |
| 155 | 585.00 | 52 | 36.36 |
| 156 | 587.00 | 82 | 57.34 |
| 157 | 588.00 | 73 | 51.05 |
| 158 | 590.00 | 54 | 37.76 |
| 159 | 591.00 | 71 | 49.65 |
| 160 | 594.00 | 58 | 40.56 |
| 161 | 596.00 | 65 | 45.45 |
| 162 | 601.00 | 55 | 38.46 |
| 163 | 608.00 | 58 | 40.56 |
| 164 | 610.00 | 63 | 44.06 |
| 165 | 612.00 | 87 | 60.84 |
| 166 | 613.00 | 52 | 36.36 |

# Peak Find - Memory-182

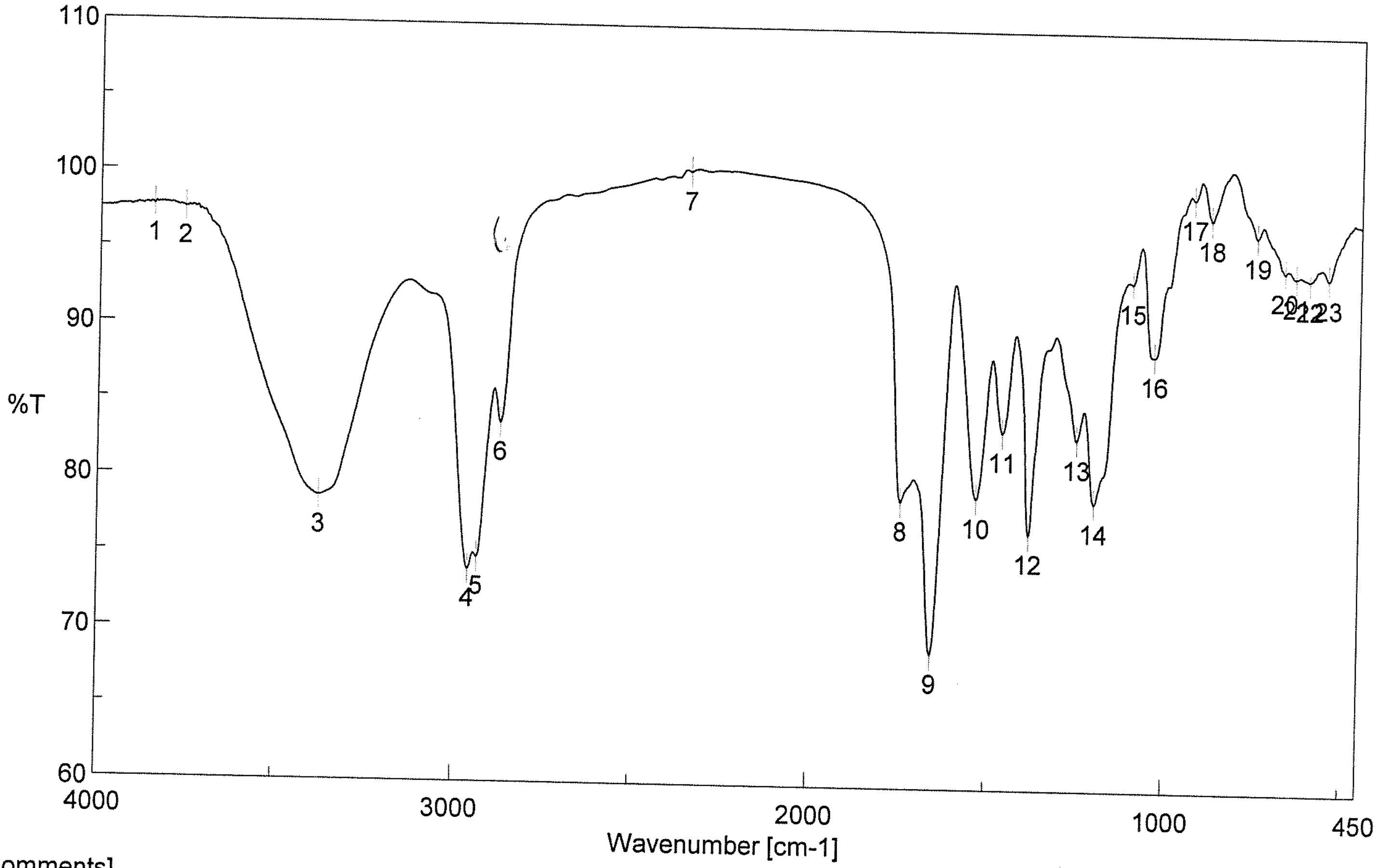


[Comments]
Sample name SH-1 = 2
Comment 24/1/2017
User IR
Division IR
Company MAC

[ Result of Peak Picking ]

| No. | Position | Intensity | No. | Position | Intensity | No. | Position | Intensity |
|---|---|---|---|---|---|---|---|---|
| 1 | 3848.26 | 97.6517 | 2 | 3759.55 | 97.5053 | 3 | 3375.78 | 78.6706 |
| 4 | 2959.23 | 73.9579 | 5 | 2934.16 | 74.7764 | 6 | 2870.52 | 83.5995 |
| 7 | 2340.19 | 100.323 | 8 | 1743.33 | 78.8584 | 9 | 1655.59 | 68.8535 |
| 10 | 1530.24 | 79.1259 | 11 | 1458.89 | 83.4826 | 12 | 1383.68 | 76.8402 |
| 13 | 1250.61 | 83.0517 | 14 | 1200.47 | 78.9007 | 15 | 1096.33 | 93.43 |
| 16 | 1031.73 | 88.635 | 17 | 923.736 | 99.061 | 18 | 874.56 | 97.6974 |
| 19 | 746.317 | 96.6093 | 20 | 668.214 | 94.333 | 21 | 634.466 | 93.993 |
| 22 | 594.932 | 93.8247 | 23 | 542.863 | 93.9123 | | | |

# Cairo University
# Micro Analytical Center

## *DI Analysis*
## *Shimadzu Qp-2010 Plus*

Sample Information

Analyzed by : Dr. Mai Younis
Analyzed : 29/01/2017 10:21:33 ص
Sample Name : Sh-1 = 2
Sample ID :
Customer Name : Dr. Gaber Osman - NRC
Data File : C:\GCMSsolution\Data\Project1\Sh-1.QGD
Org Data File : C:\GCMSsolution\Data\Project1\Sh-1.QGD
Method File : C:\GCMSsolution\Data\Project1\High Temperature Op
Org Method File : C:\GCMSsolution\Data\Project1\High Temperature Op
Report File :
Tuning File : C:\GCMSsolution\System\Tune1_default.qgt
$EndIf$Modified by : Dr. Mai Younis
Modified : 29/01/2017 10:26:55 ص

Method

===== Analytical Line 1 =====
IonSourceTemp :250.00 °C
[MS Table]
--Group 1 - Event 1--
Start Time :0.00min
End Time :10.00min
ACQ Mode :Scan
Event Time :0.50sec
Scan Speed :2500
Start m/z :50.00
End m/z :1090.00

Electron Voltage : 70 eV
Ionization Mode : EI

C:\GCMSsolution\Data\Project1\Sh-1.QGD

Chromatogram Sh-1 C:\GCMSsolution\Data\Project1\Sh-1.QGD

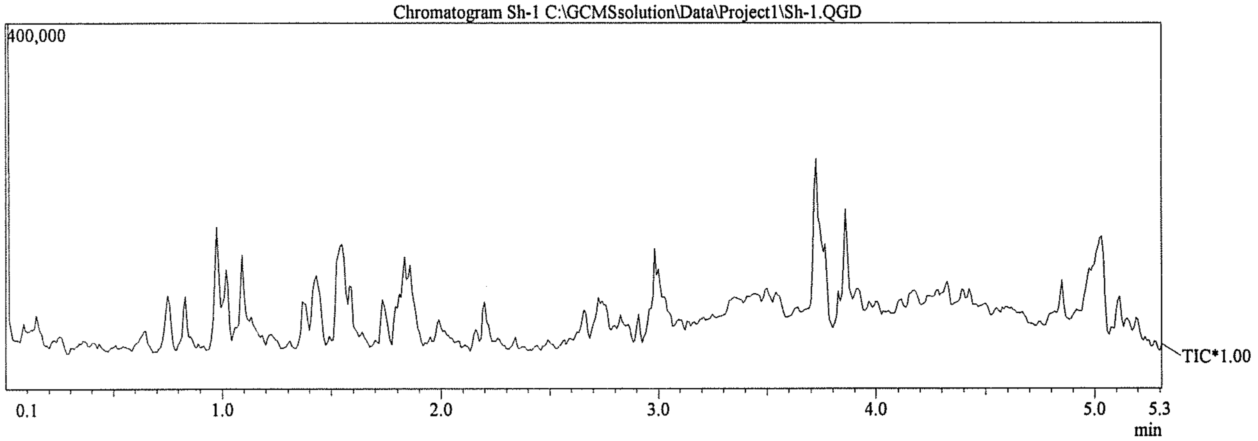


Spectrum

Line#:1 R.Time:4.3(Scan#:512)
MassPeaks:442
RawMode:Single 4.3(512) BasePeak:613(13053)
BG Mode:None Group 1 - Event 1

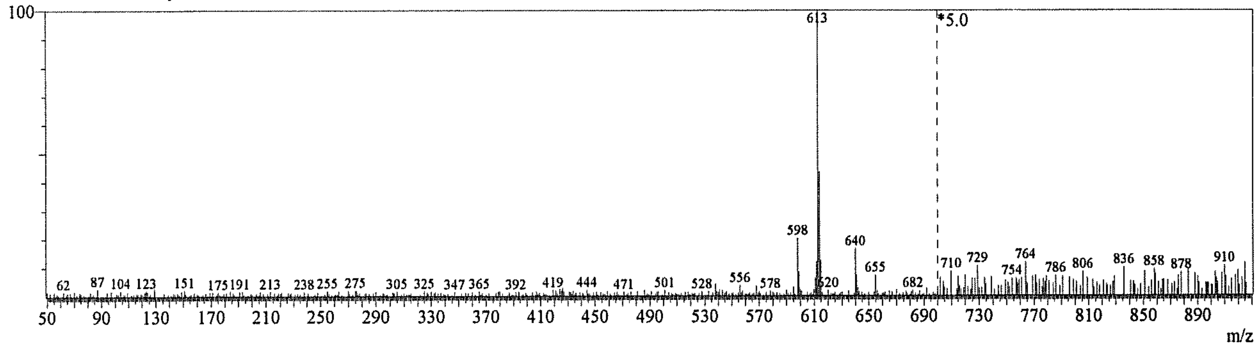


Mass Table
Line#:1 R.Time:4.3(Scan#:512)
MassPeaks:442
RawMode:Single 4.3(512) BasePeak:613(13053)
BG Mode:None Group 1 - Event 1

| # | m/z | Abs. In | Rel. Int. | # | m/z | Abs. In | Rel. Int. | # | m/z | Abs. In | Rel. Int. |
|---|---|---|---|---|---|---|---|---|---|---|---|
| 1 | 50.00 | 188 | 1.44 | 4 | 57.00 | 102 | 0.78 | 7 | 64.00 | 92 | 0.70 |
| 2 | 52.00 | 140 | 1.07 | 5 | 58.00 | 52 | 0.40 | 8 | 65.00 | 158 | 1.21 |
| 3 | 55.00 | 169 | 1.29 | 6 | 62.00 | 209 | 1.60 | 9 | 67.00 | 161 | 1.23 |

| # | m/z | Abs. Int | Rel. Int. | # | m/z | Abs. Int | Rel. Int. | # | m/z | Abs. Int | Rel. Int. |
|---|---|---|---|---|---|---|---|---|---|---|---|
| 10 | 70.00 | 212 | 1.62 | 79 | 206.00 | 201 | 1.54 | 148 | 355.00 | 148 | 1.13 |
| 11 | 72.00 | 98 | 0.75 | 80 | 208.00 | 108 | 0.83 | 149 | 357.00 | 126 | 0.97 |
| 12 | 74.00 | 167 | 1.28 | 81 | 211.00 | 124 | 0.95 | 150 | 360.00 | 162 | 1.24 |
| 13 | 75.00 | 140 | 1.07 | 82 | 213.00 | 204 | 1.56 | 151 | 362.00 | 68 | 0.52 |
| 14 | 77.00 | 94 | 0.72 | 83 | 216.00 | 132 | 1.01 | 152 | 365.00 | 206 | 1.58 |
| 15 | 80.00 | 62 | 0.47 | 84 | 218.00 | 68 | 0.52 | 153 | 366.00 | 71 | 0.54 |
| 16 | 81.00 | 62 | 0.47 | 85 | 219.00 | 151 | 1.16 | 154 | 367.00 | 126 | 0.97 |
| 17 | 82.00 | 191 | 1.46 | 86 | 220.00 | 100 | 0.77 | 155 | 370.00 | 95 | 0.73 |
| 18 | 83.00 | 57 | 0.44 | 87 | 221.00 | 166 | 1.27 | 156 | 372.00 | 111 | 0.85 |
| 19 | 84.00 | 106 | 0.81 | 88 | 226.00 | 164 | 1.26 | 157 | 377.00 | 135 | 1.03 |
| 20 | 87.00 | 354 | 2.71 | 89 | 227.00 | 81 | 0.62 | 158 | 379.00 | 207 | 1.59 |
| 21 | 94.00 | 186 | 1.42 | 90 | 228.00 | 143 | 1.10 | 159 | 380.00 | 185 | 1.42 |
| 22 | 97.00 | 214 | 1.64 | 91 | 231.00 | 94 | 0.72 | 160 | 382.00 | 121 | 0.93 |
| 23 | 101.00 | 129 | 0.99 | 92 | 233.00 | 106 | 0.81 | 161 | 387.00 | 153 | 1.17 |
| 24 | 102.00 | 97 | 0.74 | 93 | 235.00 | 92 | 0.70 | 162 | 389.00 | 54 | 0.41 |
| 25 | 104.00 | 263 | 2.01 | 94 | 238.00 | 193 | 1.48 | 163 | 390.00 | 58 | 0.44 |
| 26 | 106.00 | 106 | 0.81 | 95 | 241.00 | 126 | 0.97 | 164 | 392.00 | 182 | 1.39 |
| 27 | 107.00 | 98 | 0.75 | 96 | 246.00 | 89 | 0.68 | 165 | 394.00 | 182 | 1.39 |
| 28 | 109.00 | 186 | 1.42 | 97 | 248.00 | 159 | 1.22 | 166 | 397.00 | 73 | 0.56 |
| 29 | 112.00 | 84 | 0.64 | 98 | 253.00 | 71 | 0.54 | 167 | 399.00 | 146 | 1.12 |
| 30 | 114.00 | 82 | 0.63 | 99 | 255.00 | 241 | 1.85 | 168 | 400.00 | 54 | 0.41 |
| 31 | 116.00 | 121 | 0.93 | 100 | 256.00 | 116 | 0.89 | 169 | 404.00 | 162 | 1.24 |
| 32 | 117.00 | 118 | 0.90 | 101 | 258.00 | 78 | 0.60 | 170 | 406.00 | 55 | 0.42 |
| 33 | 119.00 | 122 | 0.93 | 102 | 260.00 | 57 | 0.44 | 171 | 407.00 | 204 | 1.56 |
| 34 | 120.00 | 103 | 0.79 | 103 | 261.00 | 95 | 0.73 | 172 | 408.00 | 79 | 0.61 |
| 35 | 121.00 | 74 | 0.57 | 104 | 262.00 | 81 | 0.62 | 173 | 409.00 | 135 | 1.03 |
| 36 | 122.00 | 175 | 1.34 | 105 | 263.00 | 222 | 1.70 | 174 | 412.00 | 145 | 1.11 |
| 37 | 123.00 | 262 | 2.01 | 106 | 265.00 | 57 | 0.44 | 175 | 413.00 | 71 | 0.54 |
| 38 | 124.00 | 220 | 1.69 | 107 | 270.00 | 262 | 2.01 | 176 | 414.00 | 71 | 0.54 |
| 39 | 125.00 | 62 | 0.47 | 108 | 271.00 | 105 | 0.80 | 177 | 419.00 | 271 | 2.08 |
| 40 | 126.00 | 114 | 0.87 | 109 | 273.00 | 78 | 0.60 | 178 | 421.00 | 231 | 1.77 |
| 41 | 129.00 | 361 | 2.77 | 110 | 275.00 | 263 | 2.01 | 179 | 422.00 | 114 | 0.87 |
| 42 | 136.00 | 146 | 1.12 | 111 | 276.00 | 230 | 1.76 | 180 | 424.00 | 316 | 2.42 |
| 43 | 139.00 | 68 | 0.52 | 112 | 277.00 | 94 | 0.72 | 181 | 425.00 | 239 | 1.83 |
| 44 | 141.00 | 74 | 0.57 | 113 | 278.00 | 134 | 1.03 | 182 | 426.00 | 350 | 2.68 |
| 45 | 143.00 | 129 | 0.99 | 114 | 282.00 | 118 | 0.90 | 183 | 427.00 | 202 | 1.55 |
| 46 | 144.00 | 97 | 0.74 | 115 | 283.00 | 153 | 1.17 | 184 | 431.00 | 193 | 1.48 |
| 47 | 146.00 | 130 | 1.00 | 116 | 285.00 | 116 | 0.89 | 185 | 432.00 | 170 | 1.30 |
| 48 | 147.00 | 79 | 0.61 | 117 | 288.00 | 111 | 0.85 | 186 | 433.00 | 102 | 0.78 |
| 49 | 149.00 | 209 | 1.60 | 118 | 290.00 | 222 | 1.70 | 187 | 434.00 | 198 | 1.52 |
| 50 | 151.00 | 273 | 2.09 | 119 | 293.00 | 54 | 0.41 | 188 | 435.00 | 68 | 0.52 |
| 51 | 152.00 | 106 | 0.81 | 120 | 295.00 | 148 | 1.13 | 189 | 436.00 | 129 | 0.99 |
| 52 | 155.00 | 57 | 0.44 | 121 | 296.00 | 121 | 0.93 | 190 | 439.00 | 146 | 1.12 |
| 53 | 156.00 | 108 | 0.83 | 122 | 297.00 | 55 | 0.42 | 191 | 441.00 | 122 | 0.93 |
| 54 | 158.00 | 145 | 1.11 | 123 | 298.00 | 74 | 0.57 | 192 | 444.00 | 282 | 2.16 |
| 55 | 160.00 | 95 | 0.73 | 124 | 302.00 | 175 | 1.34 | 193 | 445.00 | 119 | 0.91 |
| 56 | 161.00 | 71 | 0.54 | 125 | 303.00 | 142 | 1.09 | 194 | 446.00 | 103 | 0.79 |
| 57 | 164.00 | 90 | 0.69 | 126 | 305.00 | 206 | 1.58 | 195 | 449.00 | 146 | 1.12 |
| 58 | 166.00 | 145 | 1.11 | 127 | 308.00 | 66 | 0.51 | 196 | 451.00 | 180 | 1.38 |
| 59 | 169.00 | 158 | 1.21 | 128 | 310.00 | 119 | 0.91 | 197 | 452.00 | 73 | 0.56 |
| 60 | 171.00 | 169 | 1.29 | 129 | 312.00 | 70 | 0.54 | 198 | 454.00 | 167 | 1.28 |
| 61 | 174.00 | 68 | 0.52 | 130 | 313.00 | 76 | 0.58 | 199 | 456.00 | 98 | 0.75 |
| 62 | 175.00 | 175 | 1.34 | 131 | 314.00 | 52 | 0.40 | 200 | 457.00 | 105 | 0.80 |
| 63 | 176.00 | 89 | 0.68 | 132 | 315.00 | 130 | 1.00 | 201 | 459.00 | 201 | 1.54 |
| 64 | 178.00 | 70 | 0.54 | 133 | 322.00 | 81 | 0.62 | 202 | 461.00 | 90 | 0.69 |
| 65 | 179.00 | 114 | 0.87 | 134 | 325.00 | 217 | 1.66 | 203 | 464.00 | 150 | 1.15 |
| 66 | 181.00 | 164 | 1.26 | 135 | 327.00 | 153 | 1.17 | 204 | 466.00 | 175 | 1.34 |
| 67 | 184.00 | 204 | 1.56 | 136 | 328.00 | 89 | 0.68 | 205 | 467.00 | 70 | 0.54 |
| 68 | 186.00 | 146 | 1.12 | 137 | 330.00 | 201 | 1.54 | 206 | 471.00 | 183 | 1.40 |
| 69 | 187.00 | 79 | 0.61 | 138 | 332.00 | 127 | 0.97 | 207 | 473.00 | 74 | 0.57 |
| 70 | 191.00 | 206 | 1.58 | 139 | 333.00 | 169 | 1.29 | 208 | 474.00 | 124 | 0.95 |
| 71 | 193.00 | 201 | 1.54 | 140 | 335.00 | 113 | 0.87 | 209 | 476.00 | 210 | 1.61 |
| 72 | 194.00 | 52 | 0.40 | 141 | 337.00 | 148 | 1.13 | 210 | 481.00 | 223 | 1.71 |
| 73 | 197.00 | 68 | 0.52 | 142 | 339.00 | 81 | 0.62 | 211 | 486.00 | 242 | 1.85 |
| 74 | 199.00 | 118 | 0.90 | 143 | 340.00 | 74 | 0.57 | 212 | 488.00 | 97 | 0.74 |
| 75 | 200.00 | 105 | 0.80 | 144 | 342.00 | 105 | 0.80 | 213 | 489.00 | 82 | 0.63 |
| 76 | 203.00 | 110 | 0.84 | 145 | 345.00 | 60 | 0.46 | 214 | 491.00 | 199 | 1.52 |
| 77 | 204.00 | 62 | 0.47 | 146 | 347.00 | 170 | 1.30 | 215 | 494.00 | 106 | 0.81 |
| 78 | 205.00 | 55 | 0.42 | 147 | 352.00 | 110 | 0.84 | 216 | 495.00 | 190 | 1.46 |

| # | m/z | Abs. In | Rel. Int. |
|---|---|---|---|
| 217 | 496.00 | 206 | 1.58 |
| 218 | 499.00 | 62 | 0.47 |
| 219 | 501.00 | 242 | 1.85 |
| 220 | 504.00 | 164 | 1.26 |
| 221 | 506.00 | 121 | 0.93 |
| 222 | 507.00 | 71 | 0.54 |
| 223 | 508.00 | 55 | 0.42 |
| 224 | 509.00 | 121 | 0.93 |
| 225 | 510.00 | 90 | 0.69 |
| 226 | 511.00 | 81 | 0.62 |
| 227 | 513.00 | 98 | 0.75 |
| 228 | 516.00 | 178 | 1.36 |
| 229 | 518.00 | 166 | 1.27 |
| 230 | 519.00 | 79 | 0.61 |
| 231 | 520.00 | 52 | 0.40 |
| 232 | 521.00 | 124 | 0.95 |
| 233 | 522.00 | 82 | 0.63 |
| 234 | 523.00 | 135 | 1.03 |
| 235 | 526.00 | 98 | 0.75 |
| 236 | 528.00 | 190 | 1.46 |
| 237 | 533.00 | 185 | 1.42 |
| 238 | 536.00 | 138 | 1.06 |
| 239 | 538.00 | 546 | 4.18 |
| 240 | 539.00 | 210 | 1.61 |
| 241 | 540.00 | 154 | 1.18 |
| 242 | 541.00 | 282 | 2.16 |
| 243 | 543.00 | 234 | 1.79 |
| 244 | 545.00 | 127 | 0.97 |
| 245 | 546.00 | 194 | 1.49 |
| 246 | 548.00 | 79 | 0.61 |
| 247 | 550.00 | 106 | 0.81 |
| 248 | 551.00 | 119 | 0.91 |
| 249 | 553.00 | 57 | 0.44 |
| 250 | 555.00 | 132 | 1.01 |
| 251 | 556.00 | 463 | 3.55 |
| 252 | 557.00 | 151 | 1.16 |
| 253 | 558.00 | 225 | 1.72 |
| 254 | 560.00 | 78 | 0.60 |
| 255 | 561.00 | 87 | 0.67 |
| 256 | 562.00 | 79 | 0.61 |
| 257 | 563.00 | 116 | 0.89 |
| 258 | 565.00 | 89 | 0.68 |
| 259 | 566.00 | 113 | 0.87 |
| 260 | 568.00 | 449 | 3.44 |
| 261 | 569.00 | 119 | 0.91 |
| 262 | 570.00 | 177 | 1.36 |
| 263 | 571.00 | 103 | 0.79 |
| 264 | 575.00 | 142 | 1.09 |
| 265 | 578.00 | 198 | 1.52 |
| 266 | 580.00 | 174 | 1.33 |
| 267 | 581.00 | 106 | 0.81 |
| 268 | 583.00 | 140 | 1.07 |
| 269 | 585.00 | 103 | 0.79 |
| 270 | 588.00 | 71 | 0.54 |
| 271 | 590.00 | 244 | 1.87 |
| 272 | 591.00 | 124 | 0.95 |
| 273 | 593.00 | 114 | 0.87 |
| 274 | 595.00 | 410 | 3.14 |
| 275 | 596.00 | 76 | 0.58 |
| 276 | 597.95 | 2640 | 20.23 |
| 277 | 598.90 | 1106 | 8.47 |
| 278 | 599.90 | 297 | 2.28 |
| 279 | 600.90 | 207 | 1.59 |
| 280 | 604.90 | 148 | 1.13 |
| 281 | 606.90 | 74 | 0.57 |
| 282 | 607.90 | 110 | 0.84 |
| 283 | 608.90 | 58 | 0.44 |
| 284 | 609.90 | 279 | 2.14 |
| 285 | 610.95 | 804 | 6.16 |
| 286 | 612.05 | 1560 | 11.95 |
| 287 | 612.95 | 13053 | 100.00 |
| 288 | 613.95 | 5674 | 43.47 |
| 289 | 615.00 | 1606 | 12.30 |
| 290 | 616.00 | 142 | 1.09 |
| 291 | 620.00 | 233 | 1.79 |
| 292 | 622.00 | 95 | 0.73 |
| 293 | 623.00 | 81 | 0.62 |
| 294 | 624.00 | 76 | 0.58 |
| 295 | 625.00 | 150 | 1.15 |
| 296 | 628.00 | 79 | 0.61 |
| 297 | 629.00 | 113 | 0.87 |
| 298 | 630.00 | 166 | 1.27 |
| 299 | 635.00 | 218 | 1.67 |
| 300 | 639.00 | 102 | 0.78 |
| 301 | 640.00 | 2143 | 16.42 |
| 302 | 640.90 | 930 | 7.12 |
| 303 | 641.90 | 351 | 2.69 |
| 304 | 642.90 | 161 | 1.23 |
| 305 | 644.90 | 158 | 1.21 |
| 306 | 646.90 | 81 | 0.62 |
| 307 | 649.90 | 135 | 1.03 |
| 308 | 653.90 | 145 | 1.11 |
| 309 | 654.95 | 937 | 7.18 |
| 310 | 655.90 | 218 | 1.67 |
| 311 | 656.90 | 90 | 0.69 |
| 312 | 659.90 | 81 | 0.62 |
| 313 | 660.90 | 113 | 0.87 |
| 314 | 661.90 | 150 | 1.15 |
| 315 | 664.90 | 202 | 1.55 |
| 316 | 666.90 | 164 | 1.26 |
| 317 | 669.90 | 265 | 2.03 |
| 318 | 671.90 | 87 | 0.67 |
| 319 | 674.90 | 114 | 0.87 |
| 320 | 676.90 | 193 | 1.48 |
| 321 | 677.90 | 73 | 0.56 |
| 322 | 679.90 | 74 | 0.57 |
| 323 | 680.90 | 169 | 1.29 |
| 324 | 681.90 | 231 | 1.77 |
| 325 | 683.90 | 102 | 0.78 |
| 326 | 685.90 | 54 | 0.41 |
| 327 | 686.90 | 202 | 1.55 |
| 328 | 688.90 | 65 | 0.50 |
| 329 | 689.90 | 71 | 0.54 |
| 330 | 691.90 | 334 | 2.56 |
| 331 | 693.90 | 55 | 0.42 |
| 332 | 696.90 | 110 | 0.84 |
| 333 | 697.90 | 70 | 0.54 |
| 334 | 699.90 | 89 | 0.68 |
| 335 | 701.90 | 164 | 1.26 |
| 336 | 703.90 | 129 | 0.99 |
| 337 | 704.90 | 73 | 0.56 |
| 338 | 706.90 | 58 | 0.44 |
| 339 | 709.90 | 223 | 1.71 |
| 340 | 713.90 | 58 | 0.44 |
| 341 | 714.90 | 177 | 1.36 |
| 342 | 715.90 | 86 | 0.66 |
| 343 | 716.90 | 57 | 0.44 |
| 344 | 718.90 | 74 | 0.57 |
| 345 | 719.90 | 188 | 1.44 |
| 346 | 721.90 | 76 | 0.58 |
| 347 | 723.90 | 55 | 0.42 |
| 348 | 724.90 | 156 | 1.20 |
| 349 | 726.90 | 154 | 1.18 |
| 350 | 728.90 | 273 | 2.09 |
| 351 | 729.90 | 71 | 0.54 |
| 352 | 731.90 | 74 | 0.57 |
| 353 | 733.90 | 161 | 1.23 |
| 354 | 734.90 | 105 | 0.80 |
| 355 | 738.90 | 170 | 1.30 |
| 356 | 740.90 | 54 | 0.41 |
| 357 | 743.90 | 92 | 0.70 |
| 358 | 745.90 | 87 | 0.67 |
| 359 | 748.90 | 138 | 1.06 |
| 360 | 750.90 | 118 | 0.90 |
| 361 | 751.90 | 76 | 0.58 |
| 362 | 753.90 | 159 | 1.22 |
| 363 | 756.90 | 154 | 1.18 |
| 364 | 757.90 | 113 | 0.87 |
| 365 | 758.90 | 140 | 1.07 |
| 366 | 760.90 | 161 | 1.23 |
| 367 | 763.90 | 305 | 2.34 |
| 368 | 764.90 | 114 | 0.87 |
| 369 | 768.90 | 175 | 1.34 |
| 370 | 770.90 | 182 | 1.39 |
| 371 | 771.90 | 110 | 0.84 |
| 372 | 773.90 | 145 | 1.11 |
| 373 | 775.90 | 79 | 0.61 |
| 374 | 776.90 | 151 | 1.16 |
| 375 | 777.90 | 130 | 1.00 |
| 376 | 778.90 | 175 | 1.34 |
| 377 | 780.90 | 129 | 0.99 |
| 378 | 783.90 | 114 | 0.87 |
| 379 | 785.90 | 183 | 1.40 |
| 380 | 788.90 | 87 | 0.67 |
| 381 | 790.90 | 172 | 1.32 |
| 382 | 795.90 | 166 | 1.27 |
| 383 | 798.90 | 145 | 1.11 |
| 384 | 800.90 | 126 | 0.97 |
| 385 | 803.90 | 84 | 0.64 |
| 386 | 805.90 | 217 | 1.66 |
| 387 | 808.90 | 161 | 1.23 |
| 388 | 812.90 | 143 | 1.10 |
| 389 | 813.90 | 71 | 0.54 |
| 390 | 815.90 | 113 | 0.87 |
| 391 | 817.90 | 90 | 0.69 |
| 392 | 820.90 | 135 | 1.03 |
| 393 | 822.90 | 105 | 0.80 |
| 394 | 823.90 | 92 | 0.70 |
| 395 | 825.90 | 95 | 0.73 |
| 396 | 827.90 | 122 | 0.93 |
| 397 | 828.90 | 174 | 1.33 |
| 398 | 835.90 | 258 | 1.98 |
| 399 | 840.90 | 129 | 0.99 |
| 400 | 842.90 | 130 | 1.00 |
| 401 | 843.90 | 98 | 0.75 |
| 402 | 845.90 | 57 | 0.44 |
| 403 | 847.90 | 103 | 0.79 |
| 404 | 850.90 | 222 | 1.70 |
| 405 | 853.90 | 73 | 0.56 |
| 406 | 855.90 | 132 | 1.01 |
| 407 | 857.90 | 239 | 1.83 |
| 408 | 858.90 | 196 | 1.50 |
| 409 | 860.90 | 122 | 0.93 |
| 410 | 862.90 | 57 | 0.44 |
| 411 | 863.90 | 142 | 1.09 |
| 412 | 864.90 | 143 | 1.10 |
| 413 | 867.90 | 137 | 1.05 |
| 414 | 870.90 | 106 | 0.81 |
| 415 | 872.90 | 121 | 0.93 |
| 416 | 874.90 | 55 | 0.42 |
| 417 | 875.90 | 178 | 1.36 |
| 418 | 877.90 | 212 | 1.62 |
| 419 | 882.90 | 234 | 1.79 |
| 420 | 887.90 | 198 | 1.52 |
| 421 | 889.90 | 172 | 1.32 |
| 422 | 890.90 | 118 | 0.90 |
| 423 | 892.90 | 55 | 0.42 |

| # | m/z | Abs. In | Rel. Int. | # | m/z | Abs. In | Rel. Int. | # | m/z | Abs. In | Rel. Int. |
|---|---|---|---|---|---|---|---|---|---|---|---|
| 424 | 895.90 | 118 | 0.90 | 431 | 904.90 | 121 | 0.93 | 438 | 919.90 | 228 | 1.75 |
| 425 | 896.90 | 110 | 0.84 | 432 | 907.90 | 201 | 1.54 | 439 | 920.90 | 95 | 0.73 |
| 426 | 897.90 | 116 | 0.89 | 433 | 909.90 | 273 | 2.09 | 440 | 922.90 | 161 | 1.23 |
| 427 | 899.90 | 100 | 0.77 | 434 | 910.90 | 177 | 1.36 | 441 | 924.90 | 300 | 2.30 |
| 428 | 900.90 | 87 | 0.67 | 435 | 912.90 | 79 | 0.61 | 442 | 925.90 | 111 | 0.85 |
| 429 | 902.90 | 215 | 1.65 | 436 | 914.90 | 153 | 1.17 | | | | |
| 430 | 903.90 | 158 | 1.21 | 437 | 917.90 | 185 | 1.42 | | | | |

# Peak Find - Memory-162

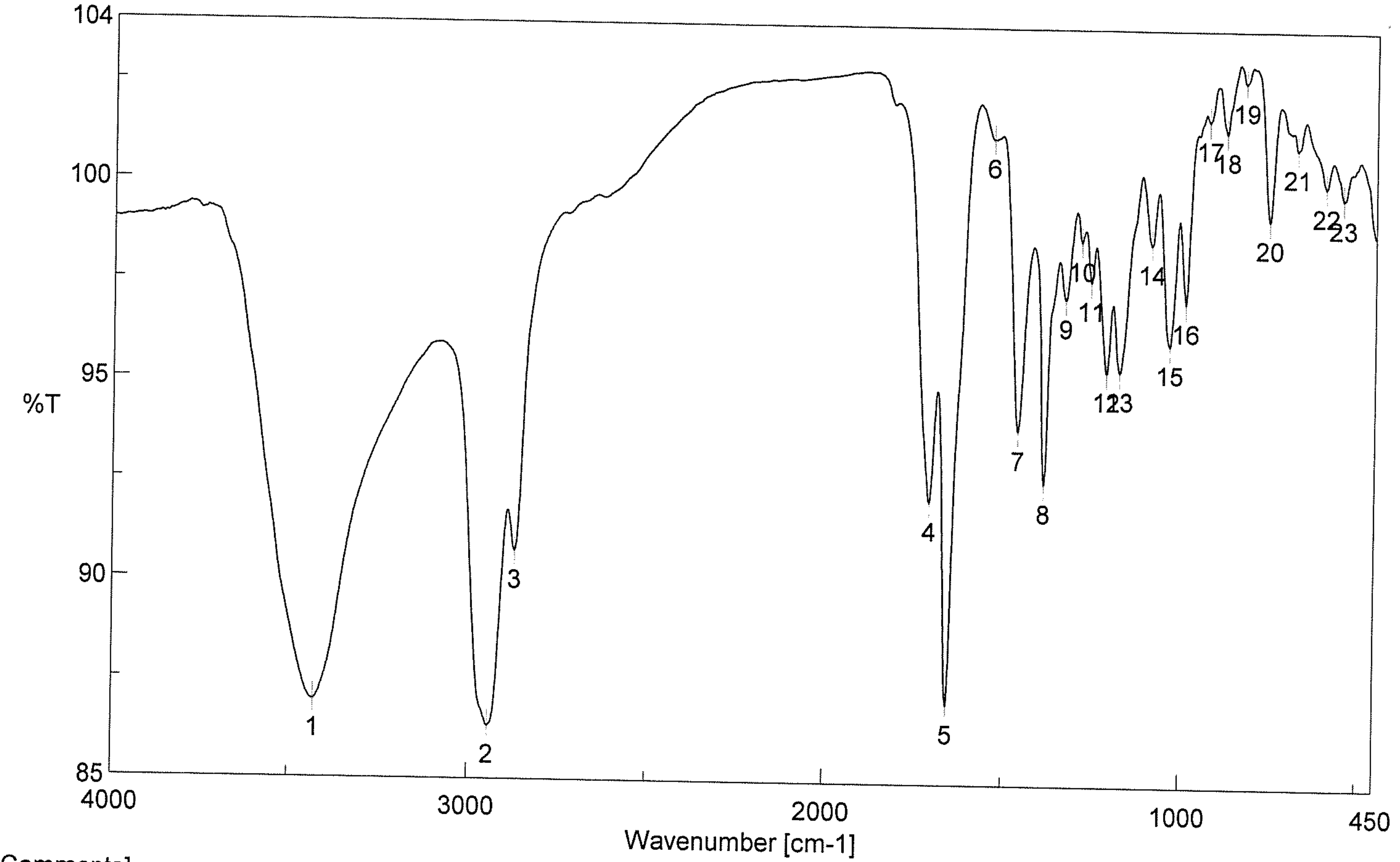


[Comments]
Sample name SH-6 = 3
Comment 24/1/2017
User IR
Division IR
Company MAC

[ Result of Peak Picking ]

| No. | Position | Intensity | No. | Position | Intensity | No. | Position | Intensity |
|---|---|---|---|---|---|---|---|---|
| 1 | 3429.78 | 86.9548 | 2 | 2943.8 | 86.3375 | 3 | 2869.56 | 90.7131 |
| 4 | 1705.73 | 92.0589 | 5 | 1655.59 | 87.0058 | 6 | 1531.2 | 101.182 |
| 7 | 1458.89 | 93.8509 | 8 | 1386.57 | 92.5409 | 9 | 1326.79 | 97.1883 |
| 10 | 1283.39 | 98.637 | 11 | 1256.4 | 97.6327 | 12 | 1212.04 | 95.3551 |
| 13 | 1174.44 | 95.3671 | 14 | 1085.73 | 98.5757 | 15 | 1033.66 | 96.0503 |
| 16 | 989.304 | 97.1092 | 17 | 923.736 | 101.707 | 18 | 874.56 | 101.419 |
| 19 | 821.527 | 102.686 | 20 | 752.102 | 99.2278 | 21 | 676.892 | 101.012 |
| 22 | 592.039 | 100.055 | 23 | 544.792 | 99.7612 | | | |

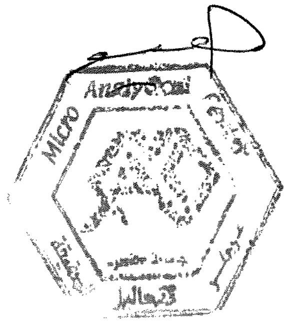

# Cairo University
# Micro Analytical Center

## *DI Analysis*
## *Shimadzu Qp-2010 Plus*

Sample Information

| | |
|---|---|
| Analyzed by | : Dr. Mai Younis |
| Analyzed | : 29/01/2017 11:41:43 ص |
| Sample Name | : Sh-6 |
| Sample ID | : |
| Customer Name | : Dr. Gaber Osman - NRC |
| Data File | : C:\GCMSsolution\Data\Project1\Sh-6.QGD |
| Org Data File | : C:\GCMSsolution\Data\Project1\Sh-6.QGD |
| Method File | : C:\GCMSsolution\Data\Project1\High Temperature Op |
| Org Method File | : C:\GCMSsolution\Data\Project1\High Temperature Op |
| Report File | : |
| Tuning File | : C:\GCMSsolution\System\Tune1_default.qgt |
| $EndIf$Modified by | : Dr. Mai Younis |
| Modified | : 29/01/2017 11:46:41 ص |

Method

===== Analytical Line 1 =====

| | |
|---|---|
| IonSourceTemp | :250.00 °C |
| [MS Table] | |
| --Group 1 - Event 1-- | |
| Start Time | :0.00min |
| End Time | :10.00min |
| ACQ Mode | :Scan |
| Event Time | :0.50sec |
| Scan Speed | :1428 |
| Start m/z | :50.00 |
| End m/z | :700.00 |
| Electron Voltage | : 70 eV |
| Ionization Mode | : EI |

C:\GCMSsolution\Data\Project1\Sh-6.QGD

Chromatogram Sh-6 C:\GCMSsolution\Data\Project1\Sh-6.QGD

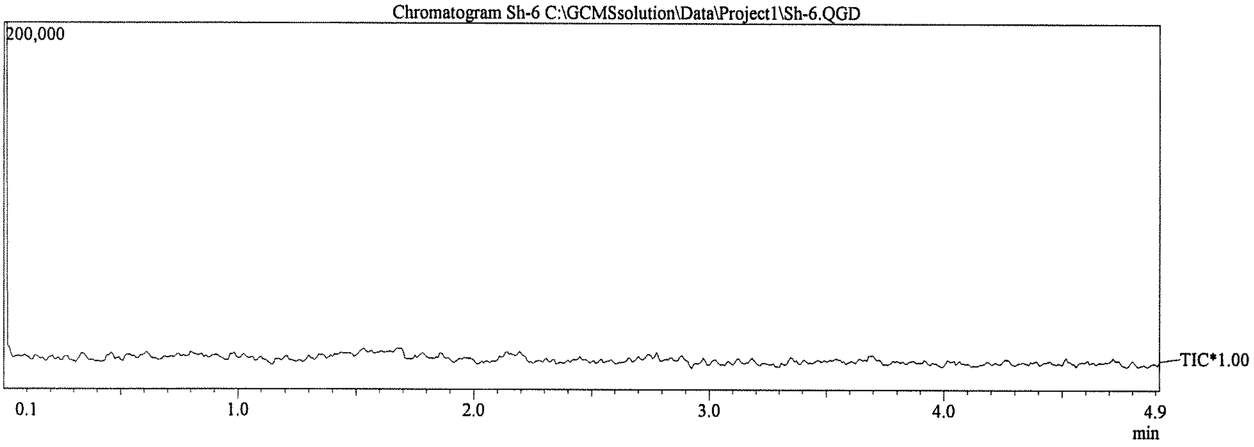


Spectrum

Line#:1 R.Time:2.8(Scan#:332)
MassPeaks:195
RawMode:Single 2.8(332) BasePeak:479(190)
BG Mode:None Group 1 - Event 1

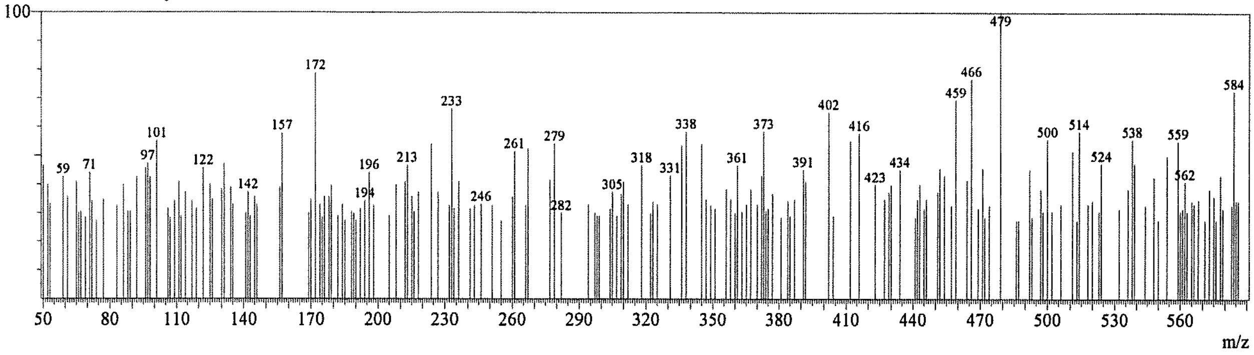


Mass Table
Line#:1 R.Time:2.8(Scan#:332)
MassPeaks:195
RawMode:Single 2.8(332) BasePeak:479(190)
BG Mode:None Group 1 - Event 1

| # | m/z | Abs. In | Rel. Int. | # | m/z | Abs. In | Rel. Int. | # | m/z | Abs. In | Rel. Int. |
|---|---|---|---|---|---|---|---|---|---|---|---|
| 1 | 50.00 | 89 | 46.84 | 4 | 59.00 | 81 | 42.63 | 7 | 66.00 | 57 | 30.00 |
| 2 | 52.00 | 76 | 40.00 | 5 | 61.00 | 68 | 35.79 | 8 | 67.00 | 58 | 30.53 |
| 3 | 53.00 | 63 | 33.16 | 6 | 65.00 | 78 | 41.05 | 9 | 69.00 | 54 | 28.42 |

| # | m/z | Abs. In | Rel. Int. |
|---|---|---|---|
| 10 | 71.00 | 84 | 44.21 |
| 11 | 72.00 | 65 | 34.21 |
| 12 | 74.00 | 52 | 27.37 |
| 13 | 77.00 | 66 | 34.74 |
| 14 | 83.00 | 62 | 32.63 |
| 15 | 86.00 | 76 | 40.00 |
| 16 | 88.00 | 58 | 30.53 |
| 17 | 89.00 | 58 | 30.53 |
| 18 | 92.00 | 81 | 42.63 |
| 19 | 96.00 | 87 | 45.79 |
| 20 | 97.00 | 90 | 47.37 |
| 21 | 98.00 | 81 | 42.63 |
| 22 | 101.00 | 105 | 55.26 |
| 23 | 106.00 | 60 | 31.58 |
| 24 | 107.00 | 54 | 28.42 |
| 25 | 109.00 | 65 | 34.21 |
| 26 | 111.00 | 78 | 41.05 |
| 27 | 112.00 | 55 | 28.95 |
| 28 | 114.00 | 71 | 37.37 |
| 29 | 117.00 | 65 | 34.21 |
| 30 | 119.00 | 60 | 31.58 |
| 31 | 122.00 | 87 | 45.79 |
| 32 | 125.00 | 76 | 40.00 |
| 33 | 126.00 | 66 | 34.74 |
| 34 | 130.00 | 73 | 38.42 |
| 35 | 131.00 | 90 | 47.37 |
| 36 | 134.00 | 74 | 38.95 |
| 37 | 135.00 | 63 | 33.16 |
| 38 | 141.00 | 57 | 30.00 |
| 39 | 142.00 | 71 | 37.37 |
| 40 | 143.00 | 55 | 28.95 |
| 41 | 145.00 | 68 | 35.79 |
| 42 | 146.00 | 63 | 33.16 |
| 43 | 156.00 | 74 | 38.95 |
| 44 | 157.00 | 110 | 57.89 |
| 45 | 169.00 | 57 | 30.00 |
| 46 | 170.00 | 66 | 34.74 |
| 47 | 172.00 | 150 | 78.95 |
| 48 | 174.00 | 63 | 33.16 |
| 49 | 175.00 | 54 | 28.42 |
| 50 | 176.00 | 68 | 35.79 |
| 51 | 178.00 | 68 | 35.79 |
| 52 | 179.00 | 76 | 40.00 |
| 53 | 182.00 | 55 | 28.95 |
| 54 | 184.00 | 63 | 33.16 |
| 55 | 185.00 | 52 | 27.37 |
| 56 | 188.00 | 58 | 30.53 |
| 57 | 189.00 | 57 | 30.00 |
| 58 | 190.00 | 52 | 27.37 |
| 59 | 192.00 | 60 | 31.58 |
| 60 | 194.00 | 65 | 34.21 |
| 61 | 196.00 | 84 | 44.21 |
| 62 | 198.00 | 62 | 32.63 |
| 63 | 205.00 | 55 | 28.95 |
| 64 | 208.00 | 76 | 40.00 |
| 65 | 212.00 | 78 | 41.05 |
| 66 | 213.00 | 89 | 46.84 |
| 67 | 215.00 | 68 | 35.79 |
| 68 | 216.00 | 58 | 30.53 |
| 69 | 218.00 | 71 | 37.37 |
| 70 | 224.00 | 103 | 54.21 |
| 71 | 227.00 | 71 | 37.37 |

| # | m/z | Abs. In | Rel. Int. |
|---|---|---|---|
| 72 | 232.00 | 62 | 32.63 |
| 73 | 233.00 | 126 | 66.32 |
| 74 | 234.00 | 60 | 31.58 |
| 75 | 236.00 | 78 | 41.05 |
| 76 | 241.00 | 60 | 31.58 |
| 77 | 243.00 | 62 | 32.63 |
| 78 | 246.00 | 63 | 33.16 |
| 79 | 251.00 | 62 | 32.63 |
| 80 | 255.00 | 52 | 27.37 |
| 81 | 260.00 | 68 | 35.79 |
| 82 | 261.00 | 98 | 51.58 |
| 83 | 266.00 | 62 | 32.63 |
| 84 | 267.00 | 100 | 52.63 |
| 85 | 277.00 | 79 | 41.58 |
| 86 | 279.00 | 103 | 54.21 |
| 87 | 282.00 | 57 | 30.00 |
| 88 | 294.00 | 63 | 33.16 |
| 89 | 297.00 | 57 | 30.00 |
| 90 | 298.00 | 55 | 28.95 |
| 91 | 299.00 | 55 | 28.95 |
| 92 | 304.00 | 60 | 31.58 |
| 93 | 305.00 | 71 | 37.37 |
| 94 | 307.00 | 55 | 28.95 |
| 95 | 309.00 | 70 | 36.84 |
| 96 | 310.00 | 78 | 41.05 |
| 97 | 312.00 | 63 | 33.16 |
| 98 | 318.00 | 89 | 46.84 |
| 99 | 322.00 | 57 | 30.00 |
| 100 | 323.00 | 65 | 34.21 |
| 101 | 325.00 | 63 | 33.16 |
| 102 | 331.00 | 82 | 43.16 |
| 103 | 336.00 | 102 | 53.68 |
| 104 | 338.00 | 111 | 58.42 |
| 105 | 345.00 | 103 | 54.21 |
| 106 | 347.00 | 66 | 34.74 |
| 107 | 349.00 | 62 | 32.63 |
| 108 | 351.00 | 60 | 31.58 |
| 109 | 356.00 | 73 | 38.42 |
| 110 | 358.00 | 66 | 34.74 |
| 111 | 360.00 | 57 | 30.00 |
| 112 | 361.00 | 89 | 46.84 |
| 113 | 363.00 | 58 | 30.53 |
| 114 | 365.00 | 63 | 33.16 |
| 115 | 367.00 | 73 | 38.42 |
| 116 | 370.00 | 63 | 33.16 |
| 117 | 372.00 | 82 | 43.16 |
| 118 | 373.00 | 111 | 58.42 |
| 119 | 374.00 | 58 | 30.53 |
| 120 | 375.00 | 60 | 31.58 |
| 121 | 377.00 | 70 | 36.84 |
| 122 | 381.00 | 54 | 28.42 |
| 123 | 384.00 | 65 | 34.21 |
| 124 | 385.00 | 55 | 28.95 |
| 125 | 387.00 | 66 | 34.74 |
| 126 | 391.00 | 86 | 45.26 |
| 127 | 392.00 | 78 | 41.05 |
| 128 | 402.00 | 124 | 65.26 |
| 129 | 404.00 | 55 | 28.95 |
| 130 | 412.00 | 105 | 55.26 |
| 131 | 416.00 | 110 | 57.89 |
| 132 | 423.00 | 76 | 40.00 |
| 133 | 427.00 | 66 | 34.74 |

| # | m/z | Abs. In | Rel. Int. |
|---|---|---|---|
| 134 | 429.00 | 71 | 37.37 |
| 135 | 430.00 | 76 | 40.00 |
| 136 | 434.00 | 86 | 45.26 |
| 137 | 441.00 | 54 | 28.42 |
| 138 | 442.00 | 66 | 34.74 |
| 139 | 443.00 | 76 | 40.00 |
| 140 | 445.00 | 60 | 31.58 |
| 141 | 446.00 | 66 | 34.74 |
| 142 | 451.00 | 71 | 37.37 |
| 143 | 452.00 | 87 | 45.79 |
| 144 | 454.00 | 82 | 43.16 |
| 145 | 457.00 | 62 | 32.63 |
| 146 | 459.00 | 132 | 69.47 |
| 147 | 464.00 | 79 | 41.58 |
| 148 | 466.00 | 146 | 76.84 |
| 149 | 469.00 | 60 | 31.58 |
| 150 | 471.00 | 87 | 45.79 |
| 151 | 472.00 | 54 | 28.42 |
| 152 | 474.00 | 62 | 32.63 |
| 153 | 479.00 | 190 | 100.00 |
| 154 | 486.00 | 52 | 27.37 |
| 155 | 487.00 | 52 | 27.37 |
| 156 | 492.00 | 86 | 45.26 |
| 157 | 493.00 | 54 | 28.42 |
| 158 | 497.00 | 73 | 38.42 |
| 159 | 498.00 | 58 | 30.53 |
| 160 | 500.00 | 106 | 55.79 |
| 161 | 502.00 | 58 | 30.53 |
| 162 | 506.00 | 63 | 33.16 |
| 163 | 511.00 | 98 | 51.58 |
| 164 | 513.00 | 52 | 27.37 |
| 165 | 514.00 | 111 | 58.42 |
| 166 | 518.00 | 63 | 33.16 |
| 167 | 520.00 | 65 | 34.21 |
| 168 | 523.00 | 58 | 30.53 |
| 169 | 524.00 | 90 | 47.37 |
| 170 | 532.00 | 60 | 31.58 |
| 171 | 536.00 | 73 | 38.42 |
| 172 | 538.00 | 106 | 55.79 |
| 173 | 539.00 | 90 | 47.37 |
| 174 | 544.00 | 62 | 32.63 |
| 175 | 548.00 | 81 | 42.63 |
| 176 | 550.00 | 52 | 27.37 |
| 177 | 554.00 | 95 | 50.00 |
| 178 | 559.00 | 105 | 55.26 |
| 179 | 560.00 | 58 | 30.53 |
| 180 | 561.00 | 60 | 31.58 |
| 181 | 562.00 | 78 | 41.05 |
| 182 | 563.00 | 58 | 30.53 |
| 183 | 565.00 | 65 | 34.21 |
| 184 | 566.00 | 63 | 33.16 |
| 185 | 568.00 | 66 | 34.74 |
| 186 | 571.00 | 52 | 27.37 |
| 187 | 573.00 | 73 | 38.42 |
| 188 | 575.00 | 68 | 35.79 |
| 189 | 576.00 | 52 | 27.37 |
| 190 | 578.00 | 82 | 43.16 |
| 191 | 579.00 | 60 | 31.58 |
| 192 | 583.00 | 62 | 32.63 |
| 193 | 584.00 | 138 | 72.63 |
| 194 | 585.00 | 65 | 34.21 |
| 195 | 586.00 | 65 | 34.21 |

# Peak Find - Memory-193

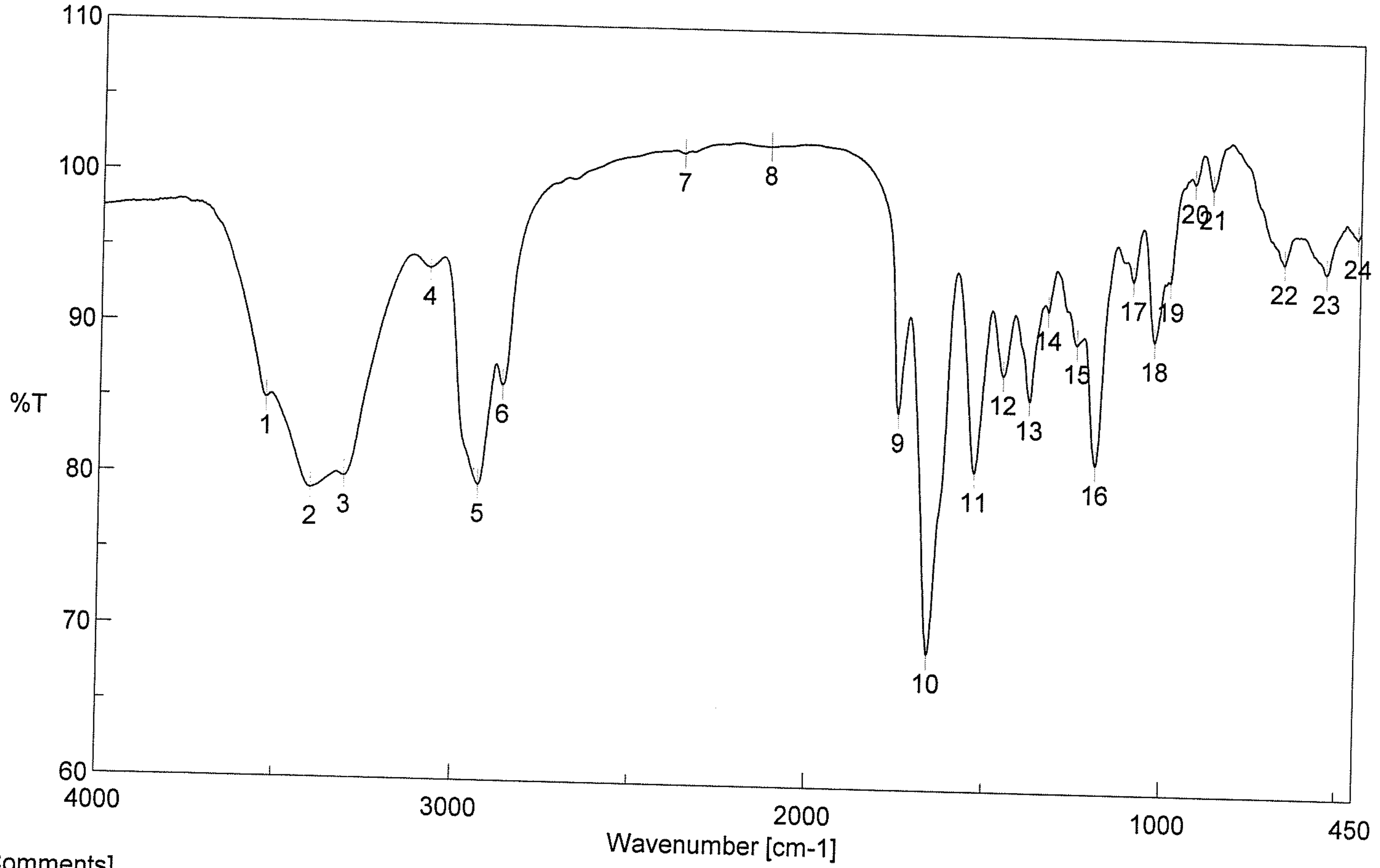


[Comments]

| | |
|---|---|
| Sample name | SH-11 = 4 |
| Comment | 24/1/2017 |
| User | IR |
| Division | IR |
| Company | MAC |

[ Result of Peak Picking ]

| No. | Position | Intensity | No. | Position | Intensity | No. | Position | Intensity |
|---|---|---|---|---|---|---|---|---|
| 1 | 3531.02 | 84.968 | 2 | 3402.78 | 79.0906 | 3 | 3310.21 | 79.8951 |
| 4 | 3075.9 | 93.779 | 5 | 2934.16 | 79.5012 | 6 | 2868.59 | 86.0632 |
| 7 | 2365.26 | 101.715 | 8 | 2121.31 | 102.281 | 9 | 1752.01 | 84.7788 |
| 10 | 1660.41 | 69 | 11 | 1535.06 | 81.006 | 12 | 1457.92 | 87.4395 |
| 13 | 1383.68 | 85.8063 | 14 | 1334.5 | 91.8111 | 15 | 1252.54 | 89.6315 |
| 16 | 1196.61 | 81.6607 | 17 | 1095.37 | 93.9249 | 18 | 1032.69 | 89.9079 |
| 19 | 990.268 | 93.8942 | 20 | 923.736 | 100.48 | 21 | 872.631 | 100.112 |
| 22 | 667.25 | 95.2613 | 23 | 545.756 | 94.7026 | 24 | 458.975 | 97.0863 |

# Cairo University
# Micro Analytical Center

## *DI Analysis*
## *Shimadzu Qp-2010 Plus*

Sample Information

| | |
|---|---|
| Analyzed by | : Dr. Mai Younis |
| Analyzed | : 29/01/2017 12:13:57 م |
| Sample Name | : Sh-11 = 4 |
| Sample ID | : |
| Customer Name | : Dr. Gaber Osman - NRC |
| Data File | : C:\GCMSsolution\Data\Project1\Sh-11.QGD |
| Org Data File | : C:\GCMSsolution\Data\Project1\Sh-11.QGD |
| Method File | : C:\GCMSsolution\Data\Project1\High Temperature Op |
| Org Method File | : C:\GCMSsolution\Data\Project1\High Temperature Op |
| Report File | : |
| Tuning File | : C:\GCMSsolution\System\Tune1_default.qgt |
| $EndIf$Modified by | : Dr. Mai Younis |
| Modified | : 29/01/2017 12:21:46 م |

Method

===== Analytical Line 1 =====

| | |
|---|---|
| IonSourceTemp | :250.00 °C |
| [MS Table] | |
| --Group 1 - Event 1-- | |
| Start Time | :0.00min |
| End Time | :10.00min |
| ACQ Mode | :Scan |
| Event Time | :0.50sec |
| Scan Speed | :1666 |
| Start m/z | :50.00 |
| End m/z | :750.00 |
| Electron Voltage | : 70 eV |
| Ionization Mode | : EI |

C:\GCMSsolution\Data\Project1\Sh-11.QGD

Chromatogram Sh-11 C:\GCMSsolution\Data\Project1\Sh-11.QGD

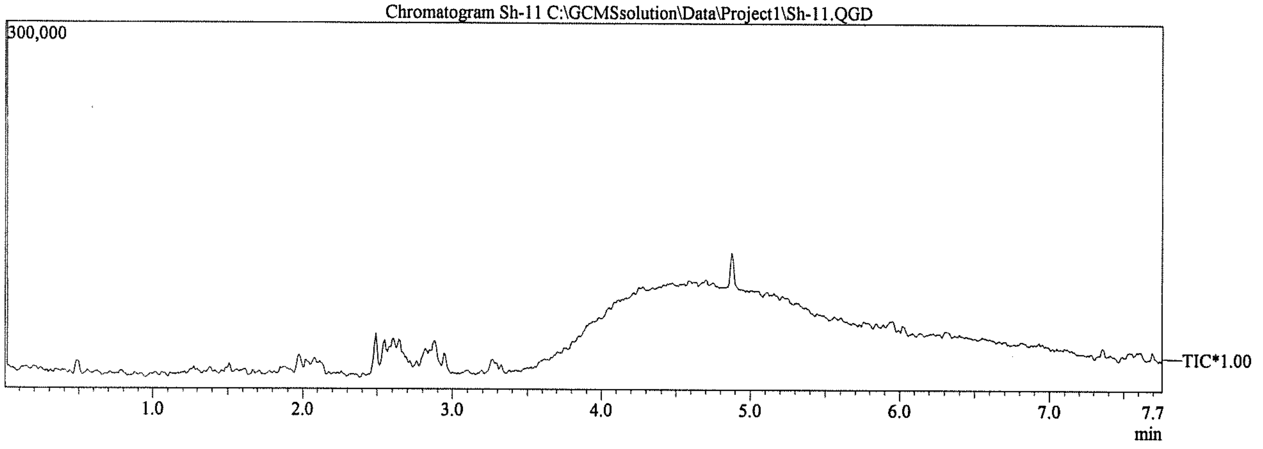


Spectrum

Line#:1 R.Time:4.2(Scan#:505)
MassPeaks:220
RawMode:Single 4.2(505) BasePeak:613(28959)
BG Mode:None Group 1 - Event 1

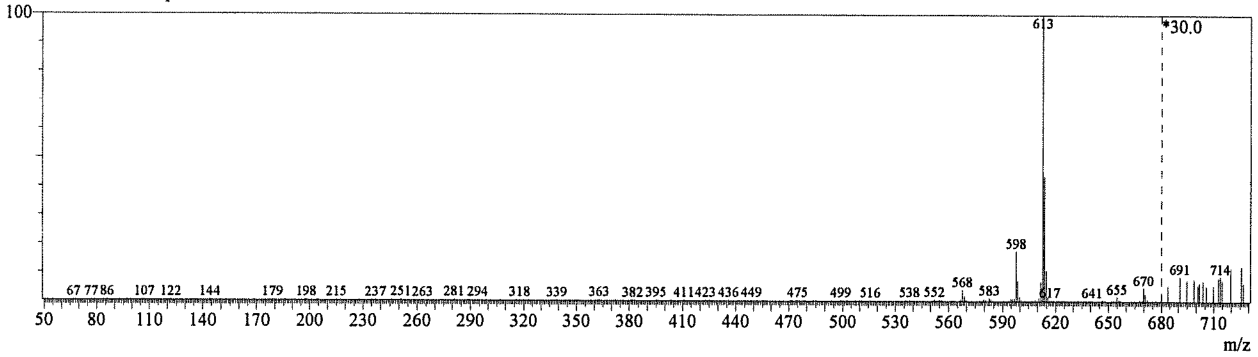


Mass Table
Line#:1 R.Time:4.2(Scan#:505)
MassPeaks:220
RawMode:Single 4.2(505) BasePeak:613(28959)
BG Mode:None Group 1 - Event 1

| # | m/z | Abs. In | Rel. Int. | # | m/z | Abs. In | Rel. Int. | # | m/z | Abs. In | Rel. Int. |
|---|---|---|---|---|---|---|---|---|---|---|---|
| 1 | 50.00 | 63 | 0.22 | 4 | 67.00 | 81 | 0.28 | 7 | 79.00 | 60 | 0.21 |
| 2 | 52.00 | 62 | 0.21 | 5 | 68.00 | 70 | 0.24 | 8 | 84.00 | 55 | 0.19 |
| 3 | 55.00 | 52 | 0.18 | 6 | 77.00 | 81 | 0.28 | 9 | 86.00 | 84 | 0.29 |

| # | m/z | Abs. In | Rel. Int. |
|---|---|---|---|
| 10 | 93.00 | 73 | 0.25 |
| 11 | 96.00 | 52 | 0.18 |
| 12 | 97.00 | 52 | 0.18 |
| 13 | 98.00 | 58 | 0.20 |
| 14 | 99.00 | 81 | 0.28 |
| 15 | 105.00 | 54 | 0.19 |
| 16 | 106.00 | 54 | 0.19 |
| 17 | 107.00 | 94 | 0.32 |
| 18 | 119.00 | 68 | 0.23 |
| 19 | 120.00 | 74 | 0.26 |
| 20 | 122.00 | 84 | 0.29 |
| 21 | 128.00 | 58 | 0.20 |
| 22 | 133.00 | 97 | 0.33 |
| 23 | 144.00 | 86 | 0.30 |
| 24 | 150.00 | 63 | 0.22 |
| 25 | 153.00 | 57 | 0.20 |
| 26 | 155.00 | 65 | 0.22 |
| 27 | 157.00 | 60 | 0.21 |
| 28 | 162.00 | 60 | 0.21 |
| 29 | 169.00 | 66 | 0.23 |
| 30 | 175.00 | 55 | 0.19 |
| 31 | 177.00 | 70 | 0.24 |
| 32 | 179.00 | 86 | 0.30 |
| 33 | 180.00 | 60 | 0.21 |
| 34 | 186.00 | 94 | 0.32 |
| 35 | 197.00 | 95 | 0.33 |
| 36 | 198.00 | 110 | 0.38 |
| 37 | 201.00 | 78 | 0.27 |
| 38 | 204.00 | 60 | 0.21 |
| 39 | 205.00 | 54 | 0.19 |
| 40 | 215.00 | 87 | 0.30 |
| 41 | 217.00 | 73 | 0.25 |
| 42 | 224.00 | 68 | 0.23 |
| 43 | 226.00 | 55 | 0.19 |
| 44 | 227.00 | 68 | 0.23 |
| 45 | 228.00 | 57 | 0.20 |
| 46 | 235.00 | 52 | 0.18 |
| 47 | 237.00 | 73 | 0.25 |
| 48 | 246.00 | 52 | 0.18 |
| 49 | 251.00 | 127 | 0.44 |
| 50 | 253.00 | 74 | 0.26 |
| 51 | 255.00 | 65 | 0.22 |
| 52 | 258.00 | 60 | 0.21 |
| 53 | 261.00 | 68 | 0.23 |
| 54 | 263.00 | 76 | 0.26 |
| 55 | 266.00 | 71 | 0.25 |
| 56 | 269.00 | 68 | 0.23 |
| 57 | 274.00 | 62 | 0.21 |
| 58 | 276.00 | 57 | 0.20 |
| 59 | 281.00 | 118 | 0.41 |
| 60 | 289.00 | 87 | 0.30 |
| 61 | 294.00 | 84 | 0.29 |
| 62 | 299.00 | 60 | 0.21 |
| 63 | 307.00 | 54 | 0.19 |
| 64 | 316.00 | 73 | 0.25 |
| 65 | 318.00 | 82 | 0.28 |
| 66 | 320.00 | 57 | 0.20 |
| 67 | 323.00 | 90 | 0.31 |
| 68 | 329.00 | 58 | 0.20 |
| 69 | 336.00 | 52 | 0.18 |
| 70 | 337.00 | 62 | 0.21 |
| 71 | 339.00 | 74 | 0.26 |
| 72 | 344.00 | 65 | 0.22 |
| 73 | 346.00 | 54 | 0.19 |
| 74 | 349.00 | 63 | 0.22 |
| 75 | 350.00 | 86 | 0.30 |
| 76 | 351.00 | 52 | 0.18 |
| 77 | 358.00 | 82 | 0.28 |
| 78 | 361.00 | 57 | 0.20 |
| 79 | 362.00 | 106 | 0.37 |
| 80 | 363.00 | 127 | 0.44 |
| 81 | 364.00 | 79 | 0.27 |
| 82 | 372.00 | 84 | 0.29 |
| 83 | 381.00 | 57 | 0.20 |
| 84 | 382.00 | 62 | 0.21 |
| 85 | 383.00 | 55 | 0.19 |
| 86 | 395.00 | 103 | 0.36 |
| 87 | 396.00 | 71 | 0.25 |
| 88 | 400.00 | 60 | 0.21 |
| 89 | 403.00 | 66 | 0.23 |
| 90 | 404.00 | 74 | 0.26 |
| 91 | 411.00 | 100 | 0.35 |
| 92 | 414.00 | 58 | 0.20 |
| 93 | 423.00 | 100 | 0.35 |
| 94 | 425.00 | 70 | 0.24 |
| 95 | 427.00 | 60 | 0.21 |
| 96 | 431.00 | 65 | 0.22 |
| 97 | 433.00 | 78 | 0.27 |
| 98 | 436.00 | 111 | 0.38 |
| 99 | 445.00 | 68 | 0.23 |
| 100 | 449.00 | 94 | 0.32 |
| 101 | 452.00 | 65 | 0.22 |
| 102 | 455.00 | 58 | 0.20 |
| 103 | 456.00 | 52 | 0.18 |
| 104 | 457.00 | 78 | 0.27 |
| 105 | 471.00 | 65 | 0.22 |
| 106 | 472.00 | 70 | 0.24 |
| 107 | 474.00 | 71 | 0.25 |
| 108 | 475.00 | 100 | 0.35 |
| 109 | 476.00 | 70 | 0.24 |
| 110 | 479.00 | 70 | 0.24 |
| 111 | 484.00 | 57 | 0.20 |
| 112 | 485.00 | 92 | 0.32 |
| 113 | 494.00 | 66 | 0.23 |
| 114 | 499.00 | 90 | 0.31 |
| 115 | 500.00 | 62 | 0.21 |
| 116 | 502.00 | 63 | 0.22 |
| 117 | 503.00 | 70 | 0.24 |
| 118 | 507.00 | 54 | 0.19 |
| 119 | 510.00 | 87 | 0.30 |
| 120 | 511.00 | 84 | 0.29 |
| 121 | 516.00 | 89 | 0.31 |
| 122 | 521.00 | 58 | 0.20 |
| 123 | 527.00 | 87 | 0.30 |
| 124 | 531.00 | 73 | 0.25 |
| 125 | 536.00 | 55 | 0.19 |
| 126 | 538.00 | 124 | 0.43 |
| 127 | 540.00 | 57 | 0.20 |
| 128 | 546.00 | 60 | 0.21 |
| 129 | 551.00 | 79 | 0.27 |
| 130 | 552.00 | 178 | 0.61 |
| 131 | 553.00 | 65 | 0.22 |
| 132 | 554.00 | 102 | 0.35 |
| 133 | 555.00 | 122 | 0.42 |
| 134 | 556.00 | 132 | 0.46 |
| 135 | 557.00 | 63 | 0.22 |
| 136 | 561.00 | 70 | 0.24 |
| 137 | 562.00 | 57 | 0.20 |
| 138 | 563.00 | 68 | 0.23 |
| 139 | 566.00 | 60 | 0.21 |
| 140 | 566.70 | 172 | 0.59 |
| 141 | 567.70 | 1162 | 4.01 |
| 142 | 568.70 | 489 | 1.69 |
| 143 | 569.70 | 146 | 0.50 |
| 144 | 570.70 | 74 | 0.26 |
| 145 | 577.70 | 54 | 0.19 |
| 146 | 578.70 | 65 | 0.22 |
| 147 | 579.70 | 238 | 0.82 |
| 148 | 580.70 | 145 | 0.50 |
| 149 | 582.70 | 330 | 1.14 |
| 150 | 583.70 | 177 | 0.61 |
| 151 | 584.70 | 74 | 0.26 |
| 152 | 590.70 | 79 | 0.27 |
| 153 | 593.70 | 78 | 0.27 |
| 154 | 594.70 | 258 | 0.89 |
| 155 | 595.70 | 183 | 0.63 |
| 156 | 596.70 | 278 | 0.96 |
| 157 | 597.75 | 5081 | 17.55 |
| 158 | 598.70 | 2095 | 7.23 |
| 159 | 599.70 | 489 | 1.69 |
| 160 | 600.70 | 126 | 0.44 |
| 161 | 602.70 | 78 | 0.27 |
| 162 | 606.70 | 55 | 0.19 |
| 163 | 608.70 | 119 | 0.41 |
| 164 | 610.70 | 351 | 1.21 |
| 165 | 611.85 | 1998 | 6.90 |
| 166 | 612.80 | 28959 | 100.00 |
| 167 | 613.80 | 12618 | 43.57 |
| 168 | 614.80 | 3124 | 10.79 |
| 169 | 615.75 | 523 | 1.81 |
| 170 | 616.80 | 118 | 0.41 |
| 171 | 617.80 | 62 | 0.21 |
| 172 | 618.80 | 86 | 0.30 |
| 173 | 620.80 | 89 | 0.31 |
| 174 | 621.80 | 90 | 0.31 |
| 175 | 622.80 | 52 | 0.18 |
| 176 | 623.80 | 111 | 0.38 |
| 177 | 624.80 | 102 | 0.35 |
| 178 | 625.80 | 52 | 0.18 |
| 179 | 628.80 | 60 | 0.21 |
| 180 | 630.80 | 57 | 0.20 |
| 181 | 632.80 | 87 | 0.30 |
| 182 | 635.80 | 73 | 0.25 |
| 183 | 636.80 | 65 | 0.22 |
| 184 | 637.80 | 68 | 0.23 |
| 185 | 639.80 | 55 | 0.19 |
| 186 | 640.80 | 89 | 0.31 |
| 187 | 645.80 | 57 | 0.20 |
| 188 | 646.80 | 150 | 0.52 |
| 189 | 647.80 | 87 | 0.30 |
| 190 | 651.80 | 86 | 0.30 |
| 191 | 652.80 | 68 | 0.23 |
| 192 | 653.80 | 95 | 0.33 |
| 193 | 654.80 | 455 | 1.57 |
| 194 | 655.80 | 210 | 0.73 |
| 195 | 656.80 | 55 | 0.19 |
| 196 | 662.80 | 63 | 0.22 |
| 197 | 664.80 | 66 | 0.23 |
| 198 | 667.80 | 132 | 0.46 |
| 199 | 668.90 | 103 | 0.36 |
| 200 | 669.90 | 1370 | 4.73 |
| 201 | 670.80 | 700 | 2.42 |
| 202 | 671.80 | 210 | 0.73 |
| 203 | 674.80 | 60 | 0.21 |
| 204 | 676.80 | 73 | 0.25 |
| 205 | 679.80 | 121 | 0.42 |
| 206 | 683.80 | 52 | 0.18 |
| 207 | 690.80 | 84 | 0.29 |
| 208 | 694.80 | 71 | 0.25 |
| 209 | 698.80 | 74 | 0.26 |
| 210 | 700.80 | 55 | 0.19 |
| 211 | 701.80 | 63 | 0.22 |
| 212 | 703.80 | 68 | 0.23 |
| 213 | 705.80 | 52 | 0.18 |
| 214 | 709.80 | 52 | 0.18 |
| 215 | 712.80 | 79 | 0.27 |
| 216 | 713.80 | 84 | 0.29 |

| # | m/z | Abs. In | Rel. Int. | # | m/z | Abs. In | Rel. Int. |
|---|---|---|---|---|---|---|---|
| 217 | 714.80 | 73 | 0.25 | 219 | 725.80 | 118 | 0.41 |
| 218 | 719.80 | 114 | 0.39 | 220 | 726.80 | 62 | 0.21 |

# Peak Find - Memory-152

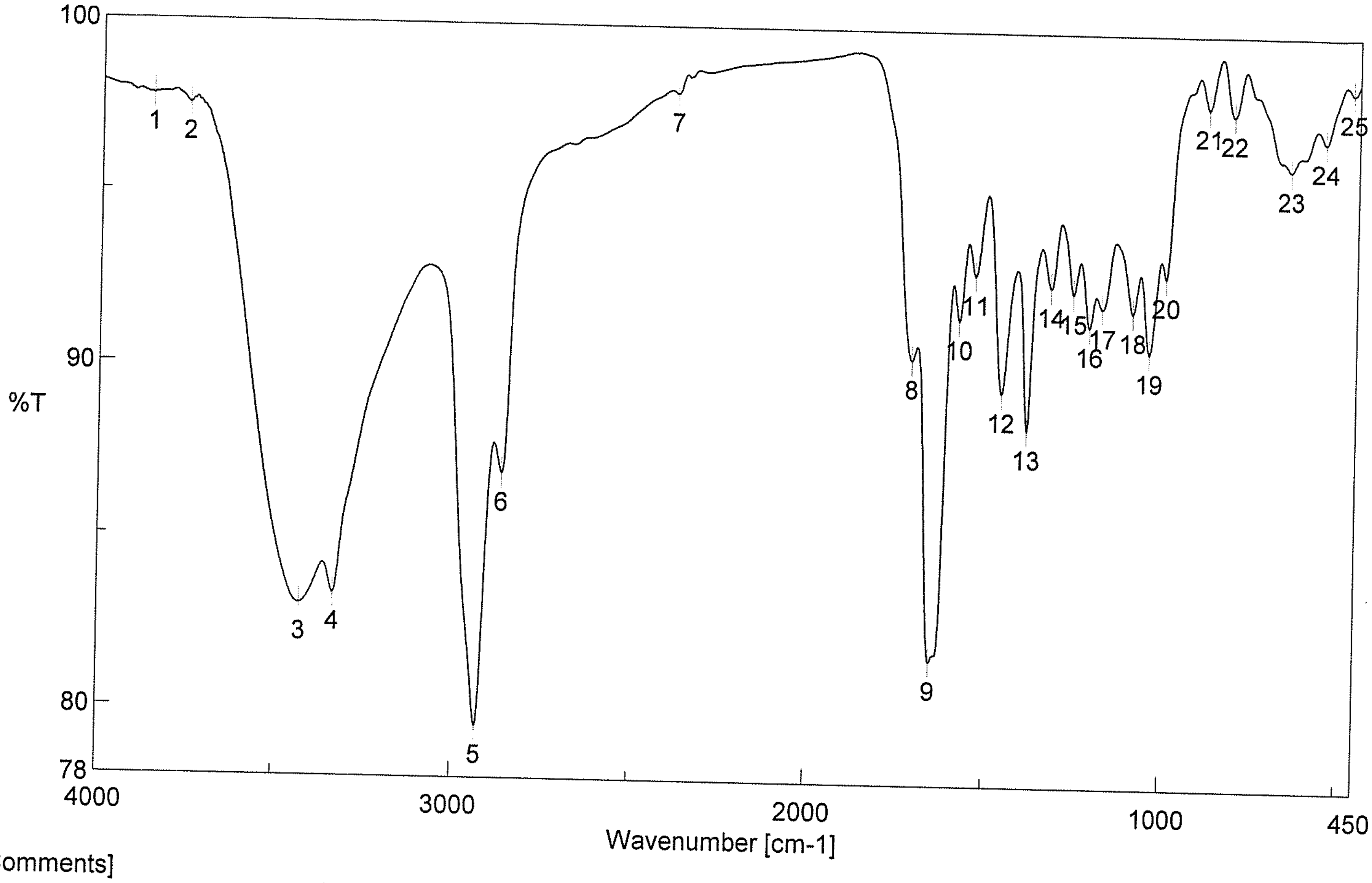


[Comments]

| | |
|---|---|
| Sample name | SH-9 = 5 |
| Comment | 24/1/2017 |
| User | IR |
| Division | IR |
| Company | MAC |

[ Result of Peak Picking ]

| No. | Position | Intensity | No. | Position | Intensity | No. | Position | Intensity |
|---|---|---|---|---|---|---|---|---|
| 1 | 3855.01 | 97.7664 | 2 | 3750.87 | 97.5152 | ✓3 | 3424.96 | 83.0301 |
| 4 | 3330.46 | 83.3404 | 5 | 2930.31 | 79.5166 | 6 | 2862.81 | 86.8743 |
| 7 | 2377.8 | 98.0177 | 8 | 1707.66 | 90.3724 | ✓9 | 1650.77 | 81.6265 |
| 10 | 1577.49 | 91.5566 | 11 | 1533.13 | 92.8651 | 12 | 1456.96 | 89.4517 |
| 13 | 1385.6 | 88.3861 | 14 | 1320.04 | 92.5549 | 15 | 1256.4 | 92.3831 |
| 16 | 1210.11 | 91.4192 | 17 | 1174.44 | 91.9701 | 18 | 1087.66 | 91.855 |
| 19 | 1039.44 | 90.6812 | 20 | 994.125 | 92.9028 | 21 | 879.381 | 97.8637 |
| 22 | 807.063 | 97.6593 | 23 | 642.179 | 96.0794 | 24 | 545.756 | 96.9008 |
| 25 | 468.617 | 98.3735 | | | | | | |

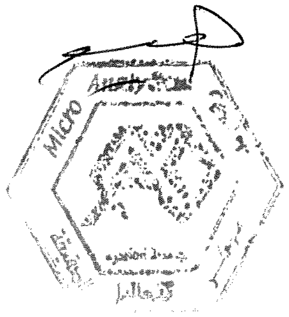

# Cairo University
# Micro Analytical Center

## *DI Analysis*
## *Shimadzu Qp-2010 Plus*

Sample Information

| | |
|---|---|
| Analyzed by | : Dr. Mai Younis |
| Analyzed | : 29/01/2017 11:53:23 ص |
| Sample Name | : Sh-9 |
| Sample ID | : |
| Customer Name | : Dr. Gaber Osman - NRC |
| Data File | : C:\GCMSsolution\Data\Project1\Sh-9.QGD |
| Org Data File | : C:\GCMSsolution\Data\Project1\Sh-9.QGD |
| Method File | : C:\GCMSsolution\Data\Project1\High Temperature Op |
| Org Method File | : C:\GCMSsolution\Data\Project1\High Temperature Op |
| Report File | : |
| Tuning File | : C:\GCMSsolution\System\Tune1_default.qgt |
| $EndIf$Modified by | : Dr. Mai Younis |
| Modified | : 29/01/2017 11:59:38 ص |

Method

==== Analytical Line 1 ====

| | |
|---|---|
| IonSourceTemp | :250.00 °C |
| [MS Table] | |
| --Group 1 - Event 1-- | |
| Start Time | :0.00min |
| End Time | :10.00min |
| ACQ Mode | :Scan |
| Event Time | :0.50sec |
| Scan Speed | :1666 |
| Start m/z | :50.00 |
| End m/z | :750.00 |
| Electron Voltage | : 70 eV |
| Ionization Mode | : EI |

C:\GCMSsolution\Data\Project1\Sh-9.QGD

Chromatogram Sh-9 C:\GCMSsolution\Data\Project1\Sh-9.QGD

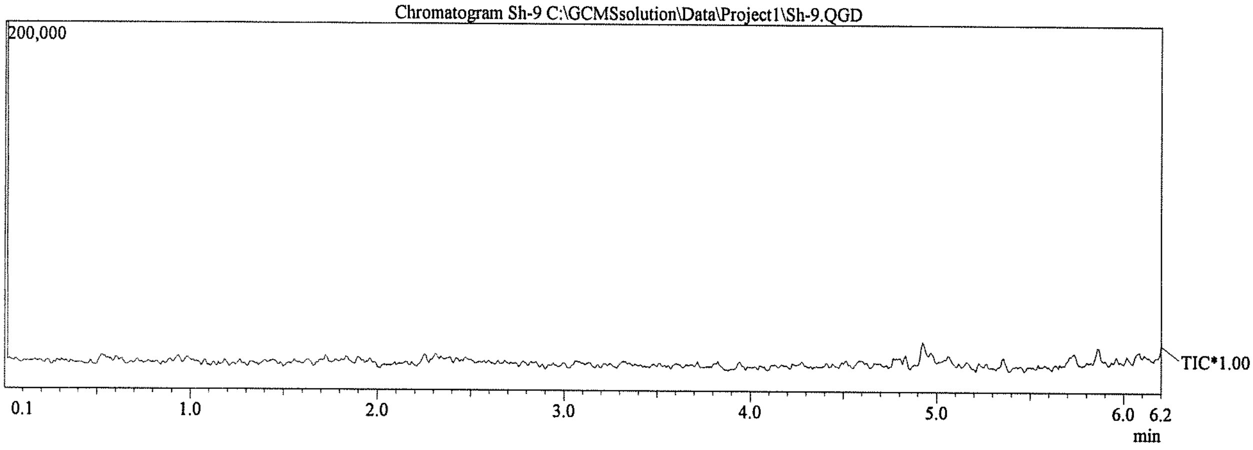


Spectrum

Line#:1 R.Time:4.3(Scan#:522)
MassPeaks:185
RawMode:Single 4.3(522) BasePeak:366(124)
BG Mode:None Group 1 - Event 1

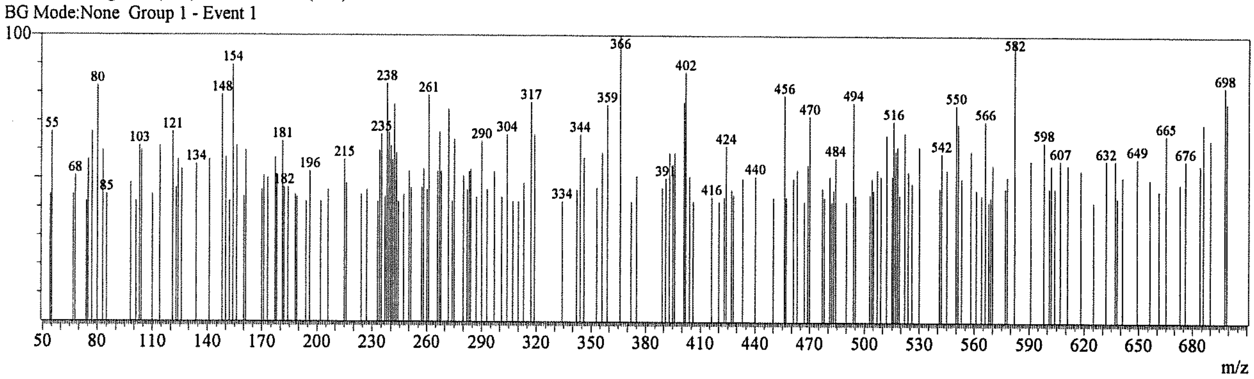


Mass Table
Line#:1 R.Time:4.3(Scan#:522)
MassPeaks:185
RawMode:Single 4.3(522) BasePeak:366(124)
BG Mode:None Group 1 - Event 1

| # | m/z | Abs. In | Rel. Int. | # | m/z | Abs. In | Rel. Int. | # | m/z | Abs. In | Rel. Int. |
|---|---|---|---|---|---|---|---|---|---|---|---|
| 1 | 54.00 | 55 | 44.35 | 4 | 68.00 | 63 | 50.81 | 7 | 77.00 | 82 | 66.13 |
| 2 | 55.00 | 82 | 66.13 | 5 | 74.00 | 52 | 41.94 | 8 | 80.00 | 102 | 82.26 |
| 3 | 67.00 | 55 | 44.35 | 6 | 75.00 | 70 | 56.45 | 9 | 83.00 | 74 | 59.68 |

| # | m/z | Abs. In | Rel. Int. |
|---|---|---|---|
| 10 | 85.00 | 55 | 44.35 |
| 11 | 98.00 | 60 | 48.39 |
| 12 | 101.00 | 52 | 41.94 |
| 13 | 103.00 | 76 | 61.29 |
| 14 | 104.00 | 74 | 59.68 |
| 15 | 110.00 | 55 | 44.35 |
| 16 | 114.00 | 76 | 61.29 |
| 17 | 121.00 | 82 | 66.13 |
| 18 | 123.00 | 58 | 46.77 |
| 19 | 124.00 | 70 | 56.45 |
| 20 | 126.00 | 66 | 53.23 |
| 21 | 134.00 | 68 | 54.84 |
| 22 | 141.00 | 70 | 56.45 |
| 23 | 148.00 | 98 | 79.03 |
| 24 | 150.00 | 71 | 57.26 |
| 25 | 152.00 | 52 | 41.94 |
| 26 | 154.00 | 111 | 89.52 |
| 27 | 156.00 | 76 | 61.29 |
| 28 | 160.00 | 54 | 43.55 |
| 29 | 161.00 | 74 | 59.68 |
| 30 | 170.00 | 57 | 45.97 |
| 31 | 171.00 | 63 | 50.81 |
| 32 | 173.00 | 62 | 50.00 |
| 33 | 177.00 | 71 | 57.26 |
| 34 | 178.00 | 63 | 50.81 |
| 35 | 181.00 | 78 | 62.90 |
| 36 | 182.00 | 58 | 46.77 |
| 37 | 184.00 | 58 | 46.77 |
| 38 | 188.00 | 55 | 44.35 |
| 39 | 189.00 | 54 | 43.55 |
| 40 | 194.00 | 52 | 41.94 |
| 41 | 196.00 | 65 | 52.42 |
| 42 | 202.00 | 52 | 41.94 |
| 43 | 206.00 | 57 | 45.97 |
| 44 | 215.00 | 70 | 56.45 |
| 45 | 216.00 | 60 | 48.39 |
| 46 | 224.00 | 55 | 44.35 |
| 47 | 227.00 | 57 | 45.97 |
| 48 | 233.00 | 52 | 41.94 |
| 49 | 234.00 | 74 | 59.68 |
| 50 | 235.00 | 81 | 65.32 |
| 51 | 237.00 | 54 | 43.55 |
| 52 | 238.00 | 103 | 83.06 |
| 53 | 239.00 | 82 | 66.13 |
| 54 | 240.00 | 76 | 61.29 |
| 55 | 241.00 | 70 | 56.45 |
| 56 | 242.00 | 94 | 75.81 |
| 57 | 243.00 | 73 | 58.87 |
| 58 | 244.00 | 52 | 41.94 |
| 59 | 247.00 | 55 | 44.35 |
| 60 | 250.00 | 65 | 52.42 |
| 61 | 251.00 | 58 | 46.77 |
| 62 | 257.00 | 58 | 46.77 |
| 63 | 258.00 | 66 | 53.23 |
| 64 | 260.00 | 57 | 45.97 |
| 65 | 261.00 | 98 | 79.03 |
| 66 | 266.00 | 65 | 52.42 |
| 67 | 267.00 | 82 | 66.13 |
| 68 | 268.00 | 65 | 52.42 |
| 69 | 272.00 | 92 | 74.19 |
| 70 | 274.00 | 52 | 41.94 |
| 71 | 275.00 | 79 | 63.71 |
| 72 | 280.00 | 63 | 50.81 |
| 73 | 282.00 | 57 | 45.97 |
| 74 | 283.00 | 65 | 52.42 |
| 75 | 284.00 | 66 | 53.23 |
| 76 | 287.00 | 54 | 43.55 |
| 77 | 290.00 | 78 | 62.90 |
| 78 | 293.00 | 57 | 45.97 |
| 79 | 297.00 | 65 | 52.42 |
| 80 | 301.00 | 54 | 43.55 |
| 81 | 304.00 | 81 | 65.32 |
| 82 | 307.00 | 52 | 41.94 |
| 83 | 310.00 | 52 | 41.94 |
| 84 | 313.00 | 60 | 48.39 |
| 85 | 317.00 | 95 | 76.61 |
| 86 | 319.00 | 81 | 65.32 |
| 87 | 334.00 | 52 | 41.94 |
| 88 | 342.00 | 57 | 45.97 |
| 89 | 344.00 | 81 | 65.32 |
| 90 | 346.00 | 71 | 57.26 |
| 91 | 353.00 | 58 | 46.77 |
| 92 | 356.00 | 73 | 58.87 |
| 93 | 359.00 | 94 | 75.81 |
| 94 | 366.00 | 124 | 100.00 |
| 95 | 372.00 | 52 | 41.94 |
| 96 | 375.00 | 63 | 50.81 |
| 97 | 389.00 | 58 | 46.77 |
| 98 | 391.00 | 62 | 50.00 |
| 99 | 393.00 | 73 | 58.87 |
| 100 | 395.00 | 68 | 54.84 |
| 101 | 396.00 | 73 | 58.87 |
| 102 | 401.00 | 95 | 76.61 |
| 103 | 402.00 | 108 | 87.10 |
| 104 | 404.00 | 63 | 50.81 |
| 105 | 406.00 | 52 | 41.94 |
| 106 | 416.00 | 54 | 43.55 |
| 107 | 420.00 | 52 | 41.94 |
| 108 | 423.00 | 54 | 43.55 |
| 109 | 424.00 | 76 | 61.29 |
| 110 | 427.00 | 57 | 45.97 |
| 111 | 428.00 | 55 | 44.35 |
| 112 | 433.00 | 62 | 50.00 |
| 113 | 440.00 | 63 | 50.81 |
| 114 | 450.00 | 54 | 43.55 |
| 115 | 456.00 | 98 | 79.03 |
| 116 | 457.00 | 54 | 43.55 |
| 117 | 461.00 | 62 | 50.00 |
| 118 | 463.00 | 66 | 53.23 |
| 119 | 467.00 | 52 | 41.94 |
| 120 | 469.00 | 68 | 54.84 |
| 121 | 470.00 | 89 | 71.77 |
| 122 | 477.00 | 58 | 46.77 |
| 123 | 478.00 | 54 | 43.55 |
| 124 | 481.00 | 63 | 50.81 |
| 125 | 482.00 | 52 | 41.94 |
| 126 | 483.00 | 57 | 45.97 |
| 127 | 484.00 | 71 | 57.26 |
| 128 | 490.00 | 52 | 41.94 |
| 129 | 494.00 | 95 | 76.61 |
| 130 | 495.00 | 55 | 44.35 |
| 131 | 503.00 | 55 | 44.35 |
| 132 | 504.00 | 62 | 50.00 |
| 133 | 505.00 | 57 | 45.97 |
| 134 | 507.00 | 66 | 53.23 |
| 135 | 509.00 | 63 | 50.81 |
| 136 | 512.00 | 81 | 65.32 |
| 137 | 515.00 | 63 | 50.81 |
| 138 | 516.00 | 87 | 70.16 |
| 139 | 517.00 | 74 | 59.68 |
| 140 | 518.00 | 76 | 61.29 |
| 141 | 519.00 | 55 | 44.35 |
| 142 | 522.00 | 82 | 66.13 |
| 143 | 524.00 | 65 | 52.42 |
| 144 | 526.00 | 60 | 48.39 |
| 145 | 530.00 | 76 | 61.29 |
| 146 | 541.00 | 58 | 46.77 |
| 147 | 542.00 | 73 | 58.87 |
| 148 | 545.00 | 66 | 53.23 |
| 149 | 550.00 | 94 | 75.81 |
| 150 | 551.00 | 86 | 69.35 |
| 151 | 553.00 | 62 | 50.00 |
| 152 | 558.00 | 74 | 59.68 |
| 153 | 561.00 | 57 | 45.97 |
| 154 | 564.00 | 55 | 44.35 |
| 155 | 566.00 | 87 | 70.16 |
| 156 | 568.00 | 52 | 41.94 |
| 157 | 569.00 | 54 | 43.55 |
| 158 | 570.00 | 68 | 54.84 |
| 159 | 577.00 | 58 | 46.77 |
| 160 | 578.00 | 63 | 50.81 |
| 161 | 582.00 | 122 | 98.39 |
| 162 | 591.00 | 70 | 56.45 |
| 163 | 598.00 | 78 | 62.90 |
| 164 | 601.00 | 58 | 46.77 |
| 165 | 602.00 | 68 | 54.84 |
| 166 | 604.00 | 58 | 46.77 |
| 167 | 607.00 | 70 | 56.45 |
| 168 | 611.00 | 68 | 54.84 |
| 169 | 618.00 | 66 | 53.23 |
| 170 | 625.00 | 52 | 41.94 |
| 171 | 632.00 | 70 | 56.45 |
| 172 | 637.00 | 70 | 56.45 |
| 173 | 638.00 | 54 | 43.55 |
| 174 | 641.00 | 63 | 50.81 |
| 175 | 649.00 | 71 | 57.26 |
| 176 | 656.00 | 62 | 50.00 |
| 177 | 661.00 | 57 | 45.97 |
| 178 | 665.00 | 81 | 65.32 |
| 179 | 673.00 | 60 | 48.39 |
| 180 | 676.00 | 70 | 56.45 |
| 181 | 684.00 | 68 | 54.84 |
| 182 | 686.00 | 86 | 69.35 |
| 183 | 690.00 | 79 | 63.71 |
| 184 | 698.00 | 102 | 82.26 |
| 185 | 699.00 | 95 | 76.61 |

# Peak Find - Memory-155

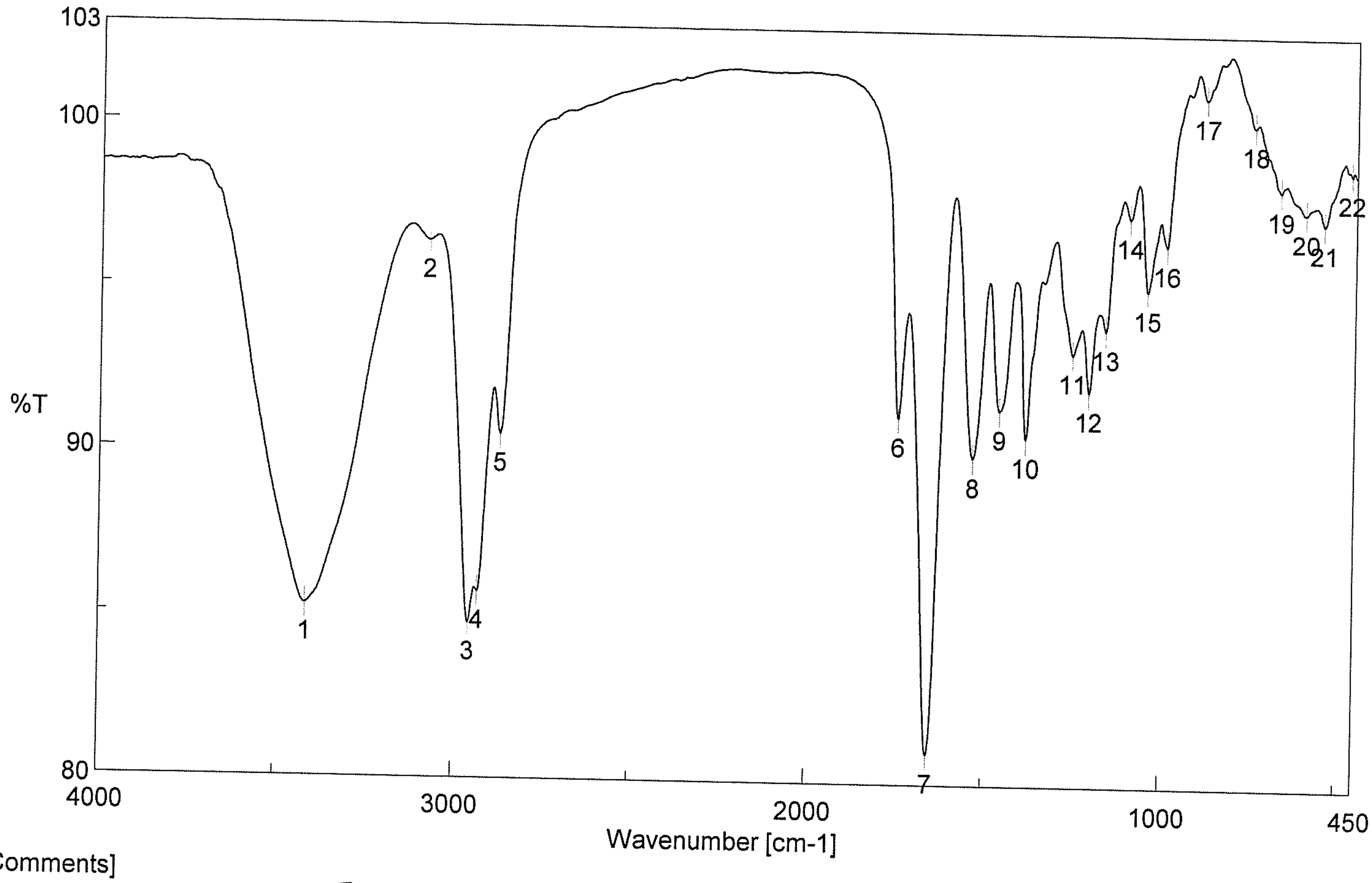


[Comments]

| | |
|---|---|
| Sample name | SH-10 = 7 |
| Comment | 24/1/2017 |
| User | IR |
| Division | IR |
| Company | MAC |

[ Result of Peak Picking ]

| No. | Position | Intensity | No. | Position | Intensity | No. | Position | Intensity |
|---|---|---|---|---|---|---|---|---|
| 1 | 3414.35 | 85.2733 | 2 | 3072.05 | 96.3645 | 3 | 2956.34 | 84.737 |
| 4 | 2931.27 | 85.6993 | 5 | 2869.56 | 90.4874 | 6 | 1744.3 | 91.1526 |
| 7 | 1656.55 | 80.9428 | 8 | 1532.17 | 89.9617 | 9 | 1458.89 | 91.4227 |
| 10 | 1384.64 | 90.5868 | 11 | 1253.5 | 93.1838 | 12 | 1207.22 | 92.0273 |
| 13 | 1159.97 | 93.9263 | 14 | 1094.4 | 97.3515 | 15 | 1043.3 | 95.1443 |
| 16 | 990.268 | 96.5226 | 17 | 880.345 | 101.067 | 18 | 742.46 | 100.231 |
| 19 | 668.214 | 98.2486 | 20 | 593.968 | 97.5846 | 21 | 542.863 | 97.255 |
| 22 | 465.725 | 98.7938 | | | | | | |

## Peak Find - Memory-174

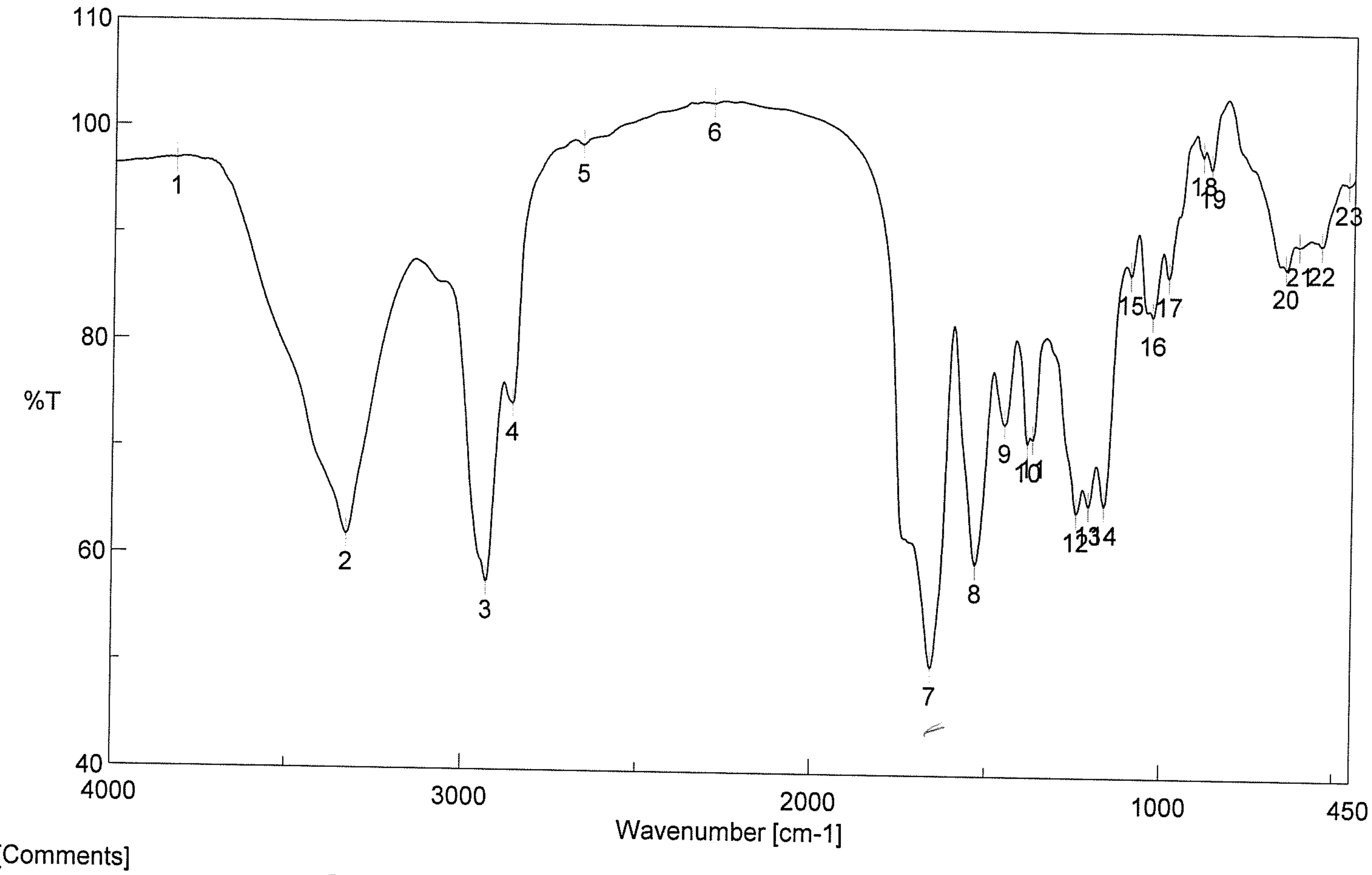


[Comments]

| | |
|---|---|
| Sample name | SH-2 = 8 |
| Comment | 24/1/2017 |
| User | IR |
| Division | IR |
| Company | MAC |

[ Result of Peak Picking ]

| No. | Position | Intensity | No. | Position | Intensity | No. | Position | Intensity |
|---|---|---|---|---|---|---|---|---|
| 1 | 3824.15 | 96.8742 | 2 | 3329.5 | 61.9202 | 3 | 2931.27 | 57.5931 |
| 4 | 2858.95 | 74.2732 | 5 | 2663.21 | 98.6153 | 6 | 2290.05 | 102.679 |
| 7 | 1658.48 | 50.1426 | 8 | 1532.17 | 59.7663 | 9 | 1452.14 | 72.9331 |
| 10 | 1386.57 | 71.1753 | 11 | 1372.1 | 71.5332 | 12 | 1246.75 | 64.7238 |
| 13 | 1212.04 | 65.377 | 14 | 1166.72 | 65.4258 | 15 | 1093.44 | 87.0638 |
| 16 | 1027.87 | 83.2525 | 17 | 983.518 | 86.9155 | 18 | 887.095 | 98.3064 |
| 19 | 862.989 | 97.1688 | 20 | 646.036 | 87.8817 | 21 | 606.503 | 89.9889 |
| 22 | 542.863 | 90.097 | 23 | 467.653 | 95.781 | | | |

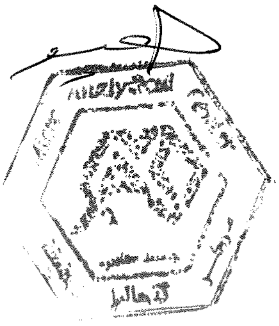

# Cairo University
# Micro Analytical Center

## *DI Analysis*
## *Shimadzu Qp-2010 Plus*

Sample Information

| | |
|---|---|
| Analyzed by | : Dr. Mai Younis |
| Analyzed | : 29/01/2017 10:34:40 ص |
| Sample Name | : Sh-2 = 8 |
| Sample ID | : |
| Customer Name | : Dr. Gaber Osman - NRC |
| Data File | : C:\GCMSsolution\Data\Project1\Sh-2.QGD |
| Org Data File | : C:\GCMSsolution\Data\Project1\Sh-2.QGD |
| Method File | : C:\GCMSsolution\Data\Project1\High Temperature Op |
| Org Method File | : C:\GCMSsolution\Data\Project1\High Temperature Op |
| Report File | : |
| Tuning File | : C:\GCMSsolution\System\Tune1_default.qgt |
| $EndIf$Modified by | : Dr. Mai Younis |
| Modified | : 29/01/2017 10:41:41 ص |

Method

==== Analytical Line 1 ====

| | |
|---|---|
| IonSourceTemp | :250.00 °C |
| [MS Table] | |
| --Group 1 - Event 1-- | |
| Start Time | :0.00min |
| End Time | :10.00min |
| ACQ Mode | :Scan |
| Event Time | :0.50sec |
| Scan Speed | :2500 |
| Start m/z | :50.00 |
| End m/z | :1090.00 |
| Electron Voltage | : 70 eV |
| Ionization Mode | : EI |

C:\GCMSsolution\Data\Project1\Sh-2.QGD

Chromatogram Sh-2 C:\GCMSsolution\Data\Project1\Sh-2.QGD

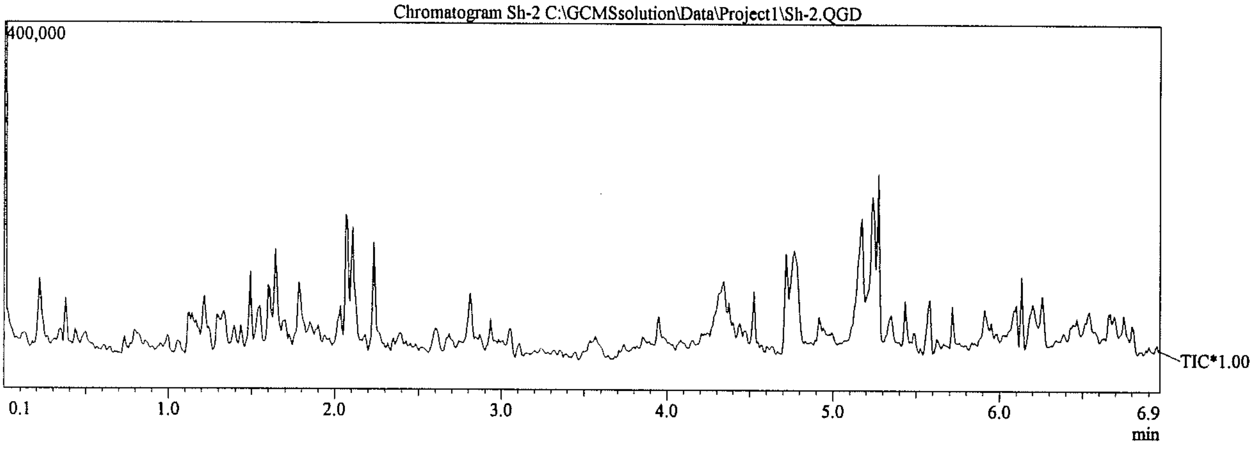


Spectrum

Line#:1 R.Time:5.2(Scan#:626)
MassPeaks:450
RawMode:Single 5.2(626) BasePeak:265(1392)
BG Mode:None Group 1 - Event 1

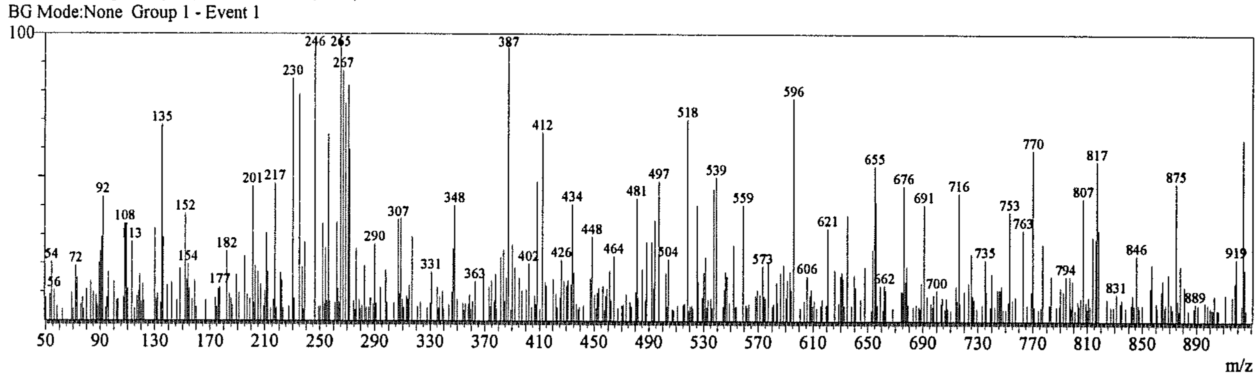


Mass Table
Line#:1 R.Time:5.2(Scan#:626)
MassPeaks:450
RawMode:Single 5.2(626) BasePeak:265(1392)
BG Mode:None Group 1 - Event 1

| # | m/z | Abs. In | Rel. Int. | # | m/z | Abs. In | Rel. Int. | # | m/z | Abs. In | Rel. Int. |
|---|---|---|---|---|---|---|---|---|---|---|---|
| 1 | 50.00 | 111 | 7.97 | 4 | 56.00 | 146 | 10.49 | 7 | 69.00 | 134 | 9.63 |
| 2 | 53.00 | 130 | 9.34 | 5 | 58.00 | 68 | 4.89 | 8 | 70.00 | 58 | 4.17 |
| 3 | 54.00 | 282 | 20.26 | 6 | 62.00 | 55 | 3.95 | 9 | 72.00 | 266 | 19.11 |

| # | m/z | Abs. In | Rel. Int. |
|---|---|---|---|
| 10 | 73.00 | 209 | 15.01 |
| 11 | 76.00 | 79 | 5.68 |
| 12 | 77.00 | 111 | 7.97 |
| 13 | 78.00 | 55 | 3.95 |
| 14 | 80.00 | 151 | 10.85 |
| 15 | 83.00 | 190 | 13.65 |
| 16 | 85.00 | 137 | 9.84 |
| 17 | 87.00 | 122 | 8.76 |
| 18 | 88.00 | 78 | 5.60 |
| 19 | 89.00 | 281 | 20.19 |
| 20 | 90.00 | 338 | 24.28 |
| 21 | 91.00 | 407 | 29.24 |
| 22 | 92.00 | 601 | 43.18 |
| 23 | 94.00 | 63 | 4.53 |
| 24 | 95.00 | 111 | 7.97 |
| 25 | 96.00 | 236 | 16.95 |
| 26 | 100.00 | 190 | 13.65 |
| 27 | 102.00 | 98 | 7.04 |
| 28 | 103.00 | 103 | 7.40 |
| 29 | 107.00 | 114 | 8.19 |
| 30 | 108.00 | 470 | 33.76 |
| 31 | 109.00 | 470 | 33.76 |
| 32 | 110.00 | 154 | 11.06 |
| 33 | 113.00 | 383 | 27.51 |
| 34 | 115.00 | 60 | 4.31 |
| 35 | 117.00 | 119 | 8.55 |
| 36 | 118.00 | 140 | 10.06 |
| 37 | 119.00 | 225 | 16.16 |
| 38 | 120.00 | 92 | 6.61 |
| 39 | 121.00 | 177 | 12.72 |
| 40 | 122.00 | 95 | 6.82 |
| 41 | 130.00 | 449 | 32.26 |
| 42 | 131.00 | 106 | 7.61 |
| 43 | 132.00 | 130 | 9.34 |
| 44 | 134.00 | 86 | 6.18 |
| 45 | 135.00 | 948 | 68.10 |
| 46 | 136.00 | 404 | 29.02 |
| 47 | 139.00 | 170 | 12.21 |
| 48 | 142.00 | 185 | 13.29 |
| 49 | 146.00 | 100 | 7.18 |
| 50 | 148.00 | 255 | 18.32 |
| 51 | 152.00 | 519 | 37.28 |
| 52 | 153.00 | 199 | 14.30 |
| 53 | 154.00 | 273 | 19.61 |
| 54 | 155.00 | 156 | 11.21 |
| 55 | 157.00 | 105 | 7.54 |
| 56 | 159.00 | 196 | 14.08 |
| 57 | 160.00 | 65 | 4.67 |
| 58 | 167.00 | 100 | 7.18 |
| 59 | 174.00 | 111 | 7.97 |
| 60 | 175.00 | 68 | 4.89 |
| 61 | 176.00 | 153 | 10.99 |
| 62 | 177.00 | 162 | 11.64 |
| 63 | 182.00 | 338 | 24.28 |
| 64 | 184.00 | 132 | 9.48 |
| 65 | 185.00 | 108 | 7.76 |
| 66 | 186.00 | 76 | 5.46 |
| 67 | 189.00 | 222 | 15.95 |
| 68 | 191.00 | 135 | 9.70 |
| 69 | 195.00 | 314 | 22.56 |
| 70 | 197.00 | 127 | 9.12 |
| 71 | 199.00 | 108 | 7.76 |
| 72 | 201.00 | 652 | 46.84 |
| 73 | 202.00 | 87 | 6.25 |
| 74 | 203.00 | 265 | 19.04 |
| 75 | 205.00 | 236 | 16.95 |
| 76 | 207.00 | 175 | 12.57 |
| 77 | 209.00 | 71 | 5.10 |
| 78 | 210.00 | 143 | 10.27 |
| 79 | 211.00 | 428 | 30.75 |
| 80 | 212.00 | 239 | 17.17 |
| 81 | 214.00 | 58 | 4.17 |
| 82 | 216.00 | 102 | 7.33 |
| 83 | 217.00 | 666 | 47.84 |
| 84 | 218.00 | 62 | 4.45 |
| 85 | 221.00 | 231 | 16.59 |
| 86 | 222.00 | 196 | 14.08 |
| 87 | 223.00 | 60 | 4.31 |
| 88 | 227.00 | 71 | 5.10 |
| 89 | 230.00 | 1175 | 84.41 |
| 90 | 231.00 | 110 | 7.90 |
| 91 | 235.00 | 1098 | 78.88 |
| 92 | 237.00 | 260 | 18.68 |
| 93 | 238.00 | 57 | 4.09 |
| 94 | 239.00 | 380 | 27.30 |
| 95 | 246.25 | 1367 | 98.20 |
| 96 | 249.20 | 65 | 4.67 |
| 97 | 250.20 | 71 | 5.10 |
| 98 | 252.20 | 473 | 33.98 |
| 99 | 253.20 | 81 | 5.82 |
| 100 | 254.20 | 354 | 25.43 |
| 101 | 255.20 | 97 | 6.97 |
| 102 | 256.20 | 905 | 65.01 |
| 103 | 257.20 | 95 | 6.82 |
| 104 | 261.20 | 86 | 6.18 |
| 105 | 262.20 | 478 | 34.34 |
| 106 | 263.20 | 154 | 11.06 |
| 107 | 264.20 | 90 | 6.47 |
| 108 | 265.15 | 1392 | 100.00 |
| 109 | 266.85 | 1212 | 87.07 |
| 110 | 268.80 | 1054 | 75.72 |
| 111 | 270.80 | 1142 | 82.04 |
| 112 | 271.80 | 830 | 59.63 |
| 113 | 274.80 | 97 | 6.97 |
| 114 | 275.80 | 54 | 3.88 |
| 115 | 276.80 | 351 | 25.22 |
| 116 | 278.80 | 102 | 7.33 |
| 117 | 279.80 | 58 | 4.17 |
| 118 | 280.80 | 73 | 5.24 |
| 119 | 282.80 | 265 | 19.04 |
| 120 | 283.80 | 66 | 4.74 |
| 121 | 285.80 | 66 | 4.74 |
| 122 | 286.80 | 110 | 7.90 |
| 123 | 288.80 | 82 | 5.89 |
| 124 | 289.80 | 372 | 26.72 |
| 125 | 290.80 | 86 | 6.18 |
| 126 | 293.80 | 161 | 11.57 |
| 127 | 297.80 | 246 | 17.67 |
| 128 | 298.80 | 87 | 6.25 |
| 129 | 303.80 | 86 | 6.18 |
| 130 | 306.80 | 490 | 35.20 |
| 131 | 307.80 | 130 | 9.34 |
| 132 | 308.80 | 497 | 35.70 |
| 133 | 309.80 | 103 | 7.40 |
| 134 | 310.80 | 66 | 4.74 |
| 135 | 312.80 | 119 | 8.55 |
| 136 | 313.80 | 105 | 7.54 |
| 137 | 314.80 | 170 | 12.21 |
| 138 | 316.80 | 407 | 29.24 |
| 139 | 320.80 | 68 | 4.89 |
| 140 | 322.80 | 89 | 6.39 |
| 141 | 327.80 | 63 | 4.53 |
| 142 | 329.80 | 86 | 6.18 |
| 143 | 330.80 | 236 | 16.95 |
| 144 | 334.80 | 161 | 11.57 |
| 145 | 335.80 | 62 | 4.45 |
| 146 | 336.80 | 114 | 8.19 |
| 147 | 338.80 | 142 | 10.20 |
| 148 | 339.80 | 58 | 4.17 |
| 149 | 343.80 | 78 | 5.60 |
| 150 | 345.80 | 137 | 9.84 |
| 151 | 346.80 | 350 | 25.14 |
| 152 | 347.80 | 559 | 40.16 |
| 153 | 349.80 | 102 | 7.33 |
| 154 | 353.80 | 84 | 6.03 |
| 155 | 354.80 | 52 | 3.74 |
| 156 | 355.80 | 82 | 5.89 |
| 157 | 357.80 | 78 | 5.60 |
| 158 | 358.80 | 124 | 8.91 |
| 159 | 359.80 | 57 | 4.09 |
| 160 | 360.80 | 94 | 6.75 |
| 161 | 362.80 | 190 | 13.65 |
| 162 | 368.80 | 217 | 15.59 |
| 163 | 372.80 | 162 | 11.64 |
| 164 | 373.80 | 70 | 5.03 |
| 165 | 374.80 | 194 | 13.94 |
| 166 | 376.80 | 90 | 6.47 |
| 167 | 377.80 | 226 | 16.24 |
| 168 | 378.80 | 58 | 4.17 |
| 169 | 381.80 | 310 | 22.27 |
| 170 | 383.80 | 343 | 24.64 |
| 171 | 384.80 | 97 | 6.97 |
| 172 | 385.80 | 207 | 14.87 |
| 173 | 387.20 | 1343 | 96.48 |
| 174 | 389.20 | 52 | 3.74 |
| 175 | 390.20 | 369 | 26.51 |
| 176 | 392.20 | 257 | 18.46 |
| 177 | 395.20 | 206 | 14.80 |
| 178 | 397.20 | 145 | 10.42 |
| 179 | 400.20 | 150 | 10.78 |
| 180 | 402.20 | 276 | 19.83 |
| 181 | 404.20 | 65 | 4.67 |
| 182 | 406.20 | 122 | 8.76 |
| 183 | 407.20 | 81 | 5.82 |
| 184 | 408.20 | 674 | 48.42 |
| 185 | 410.20 | 55 | 3.95 |
| 186 | 412.20 | 913 | 65.59 |
| 187 | 414.20 | 186 | 13.36 |
| 188 | 415.20 | 167 | 12.00 |
| 189 | 420.20 | 201 | 14.44 |
| 190 | 422.20 | 129 | 9.27 |
| 191 | 423.20 | 66 | 4.74 |
| 192 | 425.20 | 111 | 7.97 |
| 193 | 426.20 | 295 | 21.19 |
| 194 | 428.20 | 191 | 13.72 |
| 195 | 430.20 | 170 | 12.21 |
| 196 | 431.20 | 196 | 14.08 |
| 197 | 433.20 | 175 | 12.57 |
| 198 | 434.20 | 566 | 40.66 |
| 199 | 435.20 | 234 | 16.81 |
| 200 | 437.20 | 81 | 5.82 |
| 201 | 438.20 | 54 | 3.88 |
| 202 | 439.20 | 66 | 4.74 |
| 203 | 442.20 | 73 | 5.24 |
| 204 | 447.20 | 204 | 14.66 |
| 205 | 448.20 | 407 | 29.24 |
| 206 | 450.20 | 127 | 9.12 |
| 207 | 451.20 | 63 | 4.53 |
| 208 | 452.20 | 135 | 9.70 |
| 209 | 453.20 | 158 | 11.35 |
| 210 | 456.20 | 167 | 12.00 |
| 211 | 457.20 | 52 | 3.74 |
| 212 | 458.20 | 118 | 8.48 |
| 213 | 459.20 | 154 | 11.06 |
| 214 | 461.20 | 242 | 17.39 |
| 215 | 462.20 | 100 | 7.18 |
| 216 | 464.20 | 314 | 22.56 |

| # | m/z | Abs. In | Rel. Int. | # | m/z | Abs. In | Rel. Int. | # | m/z | Abs. In | Rel. Int. |
|---|---|---|---|---|---|---|---|---|---|---|---|
| 217 | 467.20 | 62 | 4.45 | 286 | 595.75 | 1079 | 77.51 | 355 | 735.00 | 298 | 21.41 |
| 218 | 470.20 | 74 | 5.32 | 287 | 600.70 | 62 | 4.45 | 356 | 736.00 | 142 | 10.20 |
| 219 | 471.20 | 76 | 5.46 | 288 | 603.70 | 95 | 6.82 | 357 | 739.00 | 70 | 5.03 |
| 220 | 473.20 | 129 | 9.27 | 289 | 605.70 | 218 | 15.66 | 358 | 740.00 | 230 | 16.52 |
| 221 | 476.20 | 90 | 6.47 | 290 | 607.70 | 122 | 8.76 | 359 | 742.00 | 71 | 5.10 |
| 222 | 477.20 | 71 | 5.10 | 291 | 608.70 | 151 | 10.85 | 360 | 744.00 | 150 | 10.78 |
| 223 | 480.20 | 138 | 9.91 | 292 | 609.70 | 74 | 5.32 | 361 | 745.00 | 151 | 10.85 |
| 224 | 481.20 | 593 | 42.60 | 293 | 610.70 | 97 | 6.97 | 362 | 746.00 | 150 | 10.78 |
| 225 | 482.20 | 113 | 8.12 | 294 | 612.70 | 63 | 4.53 | 363 | 747.00 | 170 | 12.21 |
| 226 | 485.20 | 250 | 17.96 | 295 | 615.70 | 74 | 5.32 | 364 | 748.00 | 57 | 4.09 |
| 227 | 487.20 | 97 | 6.97 | 296 | 616.70 | 105 | 7.54 | 365 | 750.00 | 54 | 3.88 |
| 228 | 488.20 | 382 | 27.44 | 297 | 619.70 | 76 | 5.46 | 366 | 752.00 | 94 | 6.75 |
| 229 | 492.20 | 382 | 27.44 | 298 | 620.70 | 450 | 32.33 | 367 | 753.00 | 529 | 38.00 |
| 230 | 493.20 | 158 | 11.35 | 299 | 625.70 | 249 | 17.89 | 368 | 755.00 | 100 | 7.18 |
| 231 | 494.20 | 489 | 35.13 | 300 | 628.70 | 87 | 6.25 | 369 | 757.00 | 118 | 8.48 |
| 232 | 497.20 | 676 | 48.56 | 301 | 629.70 | 218 | 15.66 | 370 | 763.00 | 442 | 31.75 |
| 233 | 502.20 | 233 | 16.74 | 302 | 630.70 | 236 | 16.95 | 371 | 766.00 | 78 | 5.60 |
| 234 | 503.20 | 71 | 5.10 | 303 | 631.70 | 204 | 14.66 | 372 | 769.00 | 142 | 10.20 |
| 235 | 504.20 | 300 | 21.55 | 304 | 632.70 | 76 | 5.46 | 373 | 770.00 | 828 | 59.48 |
| 236 | 507.20 | 55 | 3.95 | 305 | 634.70 | 513 | 36.85 | 374 | 771.00 | 68 | 4.89 |
| 237 | 508.20 | 52 | 3.74 | 306 | 637.70 | 74 | 5.32 | 375 | 774.00 | 54 | 3.88 |
| 238 | 511.20 | 74 | 5.32 | 307 | 639.70 | 223 | 16.02 | 376 | 776.00 | 65 | 4.67 |
| 239 | 515.20 | 78 | 5.60 | 308 | 640.70 | 162 | 11.64 | 377 | 777.00 | 374 | 26.87 |
| 240 | 516.20 | 84 | 6.03 | 309 | 644.70 | 106 | 7.61 | 378 | 782.00 | 78 | 5.60 |
| 241 | 518.20 | 977 | 70.19 | 310 | 647.70 | 263 | 18.89 | 379 | 783.00 | 218 | 15.66 |
| 242 | 519.20 | 52 | 3.74 | 311 | 652.70 | 52 | 3.74 | 380 | 787.00 | 70 | 5.03 |
| 243 | 520.20 | 71 | 5.10 | 312 | 653.70 | 346 | 24.86 | 381 | 789.00 | 74 | 5.32 |
| 244 | 521.20 | 74 | 5.32 | 313 | 655.00 | 750 | 53.88 | 382 | 790.00 | 162 | 11.64 |
| 245 | 522.20 | 62 | 4.45 | 314 | 656.00 | 577 | 41.45 | 383 | 792.00 | 142 | 10.20 |
| 246 | 525.20 | 561 | 40.30 | 315 | 657.00 | 78 | 5.60 | 384 | 794.00 | 217 | 15.59 |
| 247 | 526.20 | 191 | 13.72 | 316 | 659.00 | 170 | 12.21 | 385 | 797.00 | 217 | 15.59 |
| 248 | 529.20 | 113 | 8.12 | 317 | 661.00 | 55 | 3.95 | 386 | 798.00 | 68 | 4.89 |
| 249 | 530.20 | 234 | 16.81 | 318 | 662.00 | 172 | 12.36 | 387 | 799.00 | 196 | 14.08 |
| 250 | 531.20 | 311 | 22.34 | 319 | 663.00 | 148 | 10.63 | 388 | 801.00 | 52 | 3.74 |
| 251 | 532.20 | 66 | 4.74 | 320 | 668.00 | 62 | 4.45 | 389 | 803.00 | 95 | 6.82 |
| 252 | 533.20 | 102 | 7.33 | 321 | 674.00 | 143 | 10.27 | 390 | 804.00 | 73 | 5.24 |
| 253 | 535.20 | 110 | 7.90 | 322 | 675.00 | 142 | 10.20 | 391 | 805.00 | 111 | 7.97 |
| 254 | 536.20 | 65 | 4.67 | 323 | 676.00 | 654 | 46.98 | 392 | 807.00 | 596 | 42.82 |
| 255 | 537.20 | 639 | 45.91 | 324 | 677.00 | 191 | 13.72 | 393 | 809.00 | 94 | 6.75 |
| 256 | 539.20 | 695 | 49.93 | 325 | 678.00 | 266 | 19.11 | 394 | 810.00 | 60 | 4.31 |
| 257 | 544.20 | 68 | 4.89 | 326 | 679.00 | 79 | 5.68 | 395 | 811.00 | 116 | 8.33 |
| 258 | 545.20 | 150 | 10.78 | 327 | 683.00 | 70 | 5.03 | 396 | 812.00 | 73 | 5.24 |
| 259 | 546.20 | 238 | 17.10 | 328 | 685.00 | 79 | 5.68 | 397 | 814.00 | 410 | 29.45 |
| 260 | 547.20 | 214 | 15.37 | 329 | 687.00 | 71 | 5.10 | 398 | 816.00 | 398 | 28.59 |
| 261 | 549.20 | 86 | 6.18 | 330 | 688.00 | 60 | 4.31 | 399 | 817.00 | 775 | 55.68 |
| 262 | 552.20 | 370 | 26.58 | 331 | 689.00 | 185 | 13.29 | 400 | 818.00 | 442 | 31.75 |
| 263 | 553.20 | 68 | 4.89 | 332 | 691.00 | 564 | 40.52 | 401 | 824.00 | 108 | 7.76 |
| 264 | 554.20 | 71 | 5.10 | 333 | 693.00 | 119 | 8.55 | 402 | 827.00 | 68 | 4.89 |
| 265 | 559.20 | 562 | 40.37 | 334 | 696.00 | 86 | 6.18 | 403 | 829.00 | 71 | 5.10 |
| 266 | 561.20 | 76 | 5.46 | 335 | 697.00 | 68 | 4.89 | 404 | 831.00 | 132 | 9.48 |
| 267 | 562.20 | 66 | 4.74 | 336 | 698.00 | 127 | 9.12 | 405 | 834.00 | 89 | 6.39 |
| 268 | 563.20 | 82 | 5.89 | 337 | 700.00 | 148 | 10.63 | 406 | 835.00 | 105 | 7.54 |
| 269 | 568.20 | 122 | 8.76 | 338 | 703.00 | 86 | 6.18 | 407 | 838.00 | 65 | 4.67 |
| 270 | 569.20 | 150 | 10.78 | 339 | 704.00 | 113 | 8.12 | 408 | 842.00 | 79 | 5.68 |
| 271 | 571.20 | 126 | 9.05 | 340 | 706.00 | 58 | 4.17 | 409 | 843.00 | 126 | 9.05 |
| 272 | 573.20 | 265 | 19.04 | 341 | 707.00 | 113 | 8.12 | 410 | 844.00 | 76 | 5.46 |
| 273 | 574.20 | 118 | 8.48 | 342 | 708.00 | 63 | 4.53 | 411 | 846.00 | 319 | 22.92 |
| 274 | 575.20 | 111 | 7.97 | 343 | 710.00 | 52 | 3.74 | 412 | 847.00 | 78 | 5.60 |
| 275 | 577.20 | 282 | 20.26 | 344 | 714.00 | 169 | 12.14 | 413 | 848.00 | 63 | 4.53 |
| 276 | 580.20 | 71 | 5.10 | 345 | 715.00 | 100 | 7.18 | 414 | 850.00 | 78 | 5.60 |
| 277 | 581.20 | 82 | 5.89 | 346 | 716.00 | 620 | 44.54 | 415 | 856.00 | 161 | 11.57 |
| 278 | 583.20 | 186 | 13.36 | 347 | 717.00 | 87 | 6.25 | 416 | 857.00 | 276 | 19.83 |
| 279 | 584.20 | 89 | 6.39 | 348 | 720.00 | 145 | 10.42 | 417 | 860.00 | 87 | 6.25 |
| 280 | 586.20 | 233 | 16.74 | 349 | 723.00 | 186 | 13.36 | 418 | 861.00 | 65 | 4.67 |
| 281 | 588.20 | 271 | 19.47 | 350 | 725.00 | 327 | 23.49 | 419 | 864.00 | 146 | 10.49 |
| 282 | 590.20 | 113 | 8.12 | 351 | 726.00 | 122 | 8.76 | 420 | 865.00 | 198 | 14.22 |
| 283 | 591.20 | 198 | 14.22 | 352 | 727.00 | 106 | 7.61 | 421 | 866.00 | 68 | 4.89 |
| 284 | 593.20 | 239 | 17.17 | 353 | 729.00 | 60 | 4.31 | 422 | 867.00 | 79 | 5.68 |
| 285 | 594.20 | 148 | 10.63 | 354 | 733.00 | 78 | 5.60 | 423 | 869.00 | 228 | 16.38 |

| # | m/z | Abs. In | Rel. Int. |
|---|---|---|---|
| 424 | 871.00 | 84 | 6.03 |
| 425 | 872.00 | 60 | 4.31 |
| 426 | 875.00 | 668 | 47.99 |
| 427 | 876.00 | 102 | 7.33 |
| 428 | 877.00 | 52 | 3.74 |
| 429 | 878.00 | 268 | 19.25 |
| 430 | 881.00 | 167 | 12.00 |
| 431 | 884.00 | 54 | 3.88 |
| 432 | 888.00 | 66 | 4.74 |
| 433 | 889.00 | 86 | 6.18 |
| 434 | 891.00 | 79 | 5.68 |
| 435 | 896.00 | 92 | 6.61 |
| 436 | 898.00 | 82 | 5.89 |
| 437 | 900.00 | 65 | 4.67 |
| 438 | 901.00 | 58 | 4.17 |
| 439 | 902.00 | 57 | 4.09 |
| 440 | 903.00 | 127 | 9.12 |
| 441 | 905.00 | 57 | 4.09 |
| 442 | 906.00 | 55 | 3.95 |
| 443 | 911.00 | 129 | 9.27 |
| 444 | 916.00 | 121 | 8.69 |
| 445 | 918.00 | 188 | 13.51 |
| 446 | 919.00 | 305 | 21.91 |
| 447 | 923.00 | 76 | 5.46 |
| 448 | 924.00 | 881 | 63.29 |
| 449 | 925.00 | 252 | 18.10 |
| 450 | 926.00 | 54 | 3.88 |
